# Identification of a Common Pharmacophore for Binding to MMP2 and RGD Integrin: Towards a Multitarget Approach to Inhibit Cancer Angiogenesis and Metastasis

**DOI:** 10.3390/molecules27041249

**Published:** 2022-02-12

**Authors:** Lorenzo Baldini, Elena Lenci, Francesca Bianchini, Andrea Trabocchi

**Affiliations:** 1 Department of Chemistry “Ugo Schiff”, University of Florence, 50019 Sesto Fiorentino, Italy; lorenzo.baldini@unifi.it (L.B.); elena.lenci@unifi.it (E.L.); 2 Department of Biomedical, Experimental and Clinical Sciences “Mario Serio”, University of Florence, 50134 Florence, Italy; francesca.bianchini@unifi.it

**Keywords:** matrix metalloproteinase, multitarget, peptidomimetics, drug discovery, cancer, organic synthesis, molecular docking

## Abstract

During tumor angiogenesis different growth factors, cytokines and other molecules interact closely with each other to facilitate tumor cell invasion and metastatic diffusion. The most intensively studied as molecular targets in anti-angiogenic therapies are vascular endothelial growth factor (VEGF) and related receptors, integrin receptors and matrix metalloproteinases (MMPs). Considering the poor efficacy of cancer angiogenesis monotherapies, we reasoned combining the inhibition of α_v_β_3_ and MMP2 as a multitarget approach to deliver a synergistic blockade of tumor cell migration, invasion and metastasis. Accordingly, we identified a common pharmacophore in the binding cavity of MMP2 and α_v_β_3_, demonstrating such approach with the design, synthesis and bioassays of tyrosine-derived peptidomimetics carrying the necessary functional groups to bind to key pharmacophoric elements of MMP2 and α_v_β_3_ RGD integrin.

## 1. Introduction

Among cancer hallmarks [1], the deadliest component is invasion and metastasis, which refers to the ability of cancer cells to propagate from the original site to other tissues and produce secondary lesions. Tumor angiogenesis is a key step of this process and refers to the development of cancer blood vessel deriving from existing vasculature. Newly formed blood cancer vessels allow both primary cancer lesion to grow and cancer cells to invade adjacent tissues, disseminate in the blood stream and spread throughout the body.

During tumor angiogenesis different growth factors, cytokines and other molecules interact closely with each other to facilitate tumor cell invasion and metastatic diffusion. Among many different pro-angiogenic factors, the most intensively studied as molecular targets in anti-angiogenic therapies are vascular endothelial growth factor (VEGF) and related receptors, integrin receptors and matrix metalloproteinases (MMPs) [2]. Nevertheless, VEGF- and VEGFR-targeted monotherapies have shown only a reduced overall survival and progression-free survival [3]. 

**Figure 1:**
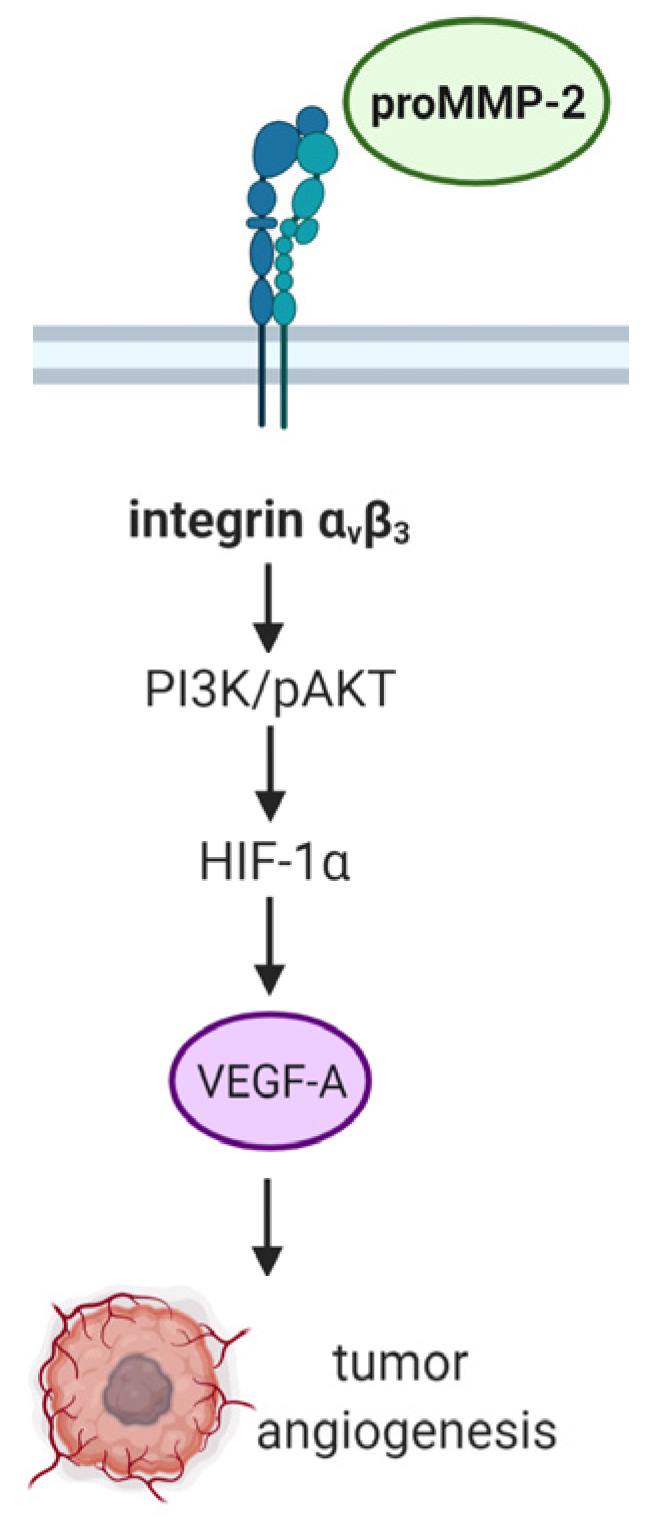
Schematic representation of the signalling cascade, initiated by the interaction between proMMP2 and integrin α_v_β_3_ on the cell surface of A549 epithelial cells (lung cancer tumour cells), leading to the expression of the major pro-angiogenic growth factor of vascular endothelium VEGF-A, which drives cellular processes such as vascularization, endothelial cell differentiation and microtubule formation.

MMP2, also known as gelatinase A, digests collagen IV of the basal lamina and is upregulated in many tumor cells and in tumor-associated endothelial cells. High levels of gelatinases are found in several human malignancies, including those of breast, brain, pancreas, colon-rectum, lung, bladder, skin, prostate [4], and are often associated with tumour aggressiveness and poor prognosis [5]. Gelatinases interact with numerous macromolecules using their different domains. With their collagen-binding domain, they bind collagen molecule and fibronectin present on the tumour cell surface [6]. With their catalytic and PEX domains, they instead bind integral membrane proteins such as the Ku protein and integrins. For example, it has been shown that in A549 lung cancer cells, MMP2 interacts with integrin α_v_β_3_ inducing the PI3/AKT signalling pathway, that activates hypoxia-induced transcription factor-1a (HIF-1a) and expression of the vascular endothelial growth factor VEGF (Figure 1) [7]. Thus, MMP2 plays an important role in tumor cells invasion and metastatic diffusion. Interestingly, α_v_β_3_ integrin co-localizes with Membrane Type 1-MMP (MT1-MMP) in correspondence with the invasive front of the tumor. Moreover, integrin α_v_β_3_ has been shown to bind the hemopexin domain of MMP2 and is reported to be involved in the activation of MMP2. Thus, the cooperation of these two macromolecules directs the localized degradation of the extracellular matrix favoring cancer cells invasiveness and metastatic diffusion [8].

Great efforts have been focused on integrin receptors and matrix metalloproteinases, in particular integrin α_v_β_3_ [9,10] and matrix metalloproteinase-2 (MMP2) [11]. It is known that proliferating endothelial cells express high levels of α_v_β_3_ integrin receptors, and their important role in angiogenesis has been elucidated. Moreover, α_v_β_3_ receptors overexpression correlates with highly metastatic phenotype of tumor cells. 

The inhibition of α_v_β_3_ integrin has been under clinical evaluation in targeted and combined anticancer therapies [12,13]. In addition, during last decades, more than fifty MMP inhibitors have been investigated in clinical trials, although all these ones failed. Reasons for failure included the lack of inhibitor specificity and insufficient knowledge about the complexity of the disease biology [14]. In this view, the successful combination of selected inhibitors of integrins and new gelatinases inhibitors may give new insight for improved strategies against tumor angiogenesis. The α_v_β_3_-MMP2 complex localized on the surface of tumor cells may play an important role in reducing the invasive activity of highly metastatic malignant cells.

Moreover, as a rising demand for more tolerable therapies drives the development of novel combination strategies, several regimens that include two or more molecularly targeted agents have recently been approved, and several combinations are in late-phase clinical development [15]. 

During the last years we developed new peptidomimetics [16] as integrin ligands, [17,18,19] and successively we moved our attention to the inhibition of MMP2/9 within the context of cancer angiogenesis and metastasis [20,21,22]. Starting from such results, we got interested in the study of the combined effect of both RGD integrin ligands and gelatinase inhibitors towards cell migration and invasion in vitro. Initial evidence supporting this idea resulted from the combined administration of a RGD peptidomimetic and a MMP2/9 inhibitor proved beneficial in improving the blockade of migration in a Boyden chamber assay (unpublished results). Thus, we reasoned developing dual inhibitors capable of simultaneously inhibiting both gelatinases and RGD integrins, since all these proteins are involved in the process of angiogenesis and tumour metastasis.

## 2. Results and Discussion

### 2.1. Rational Design of Dual MMP2 and α_v_β_3_ Integrin Ligands 

**Figure 2:**
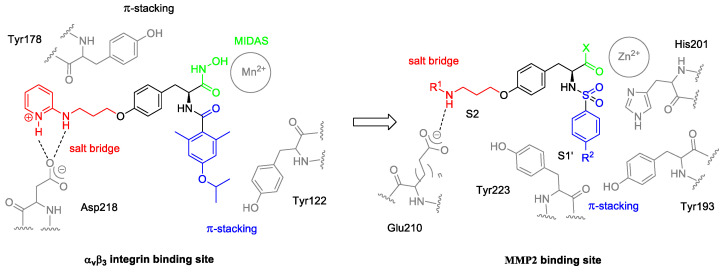
Design of multitarget α_v_β_3_-MMP2 tyrosine-derived peptidomimetics; (**left**) binding mode of Kessler’s peptidomimetic within α_v_β_3_; (**right**) hypothesized binding mode of designed sulfonamido tyrosine peptidomimetics within MMP2 catalytic site; R^1^ = H, C(NH)NH_2_, R^2^ = H, Ph, NO_2_, X = OH, NHOH.

The application of dual α_v_β_3_-MMP2 peptidomimetic inhibitors drew inspiration from a work reported by Kessler and collaborators [23], who developed ligands based on the tyrosine scaffold and studied the effect of the hydroxamic acid group as compared to the carboxylic acid group for the interaction with RGD integrins. Specifically, starting from the knowledge of the binding mode towards α_v_β_3_ integrin we reasoned such pharmacophore could be found in the MMP2 catalytic site, too. The tyrosine-based RGD peptidomimetic developed by Kessler consists of a carboxylic or hydroxamic acid moiety chelating the metal ion, two aryl rings promoting π-stacking interactions and a basic group at the opposite site of the molecule interacting with aspartic acids with salt bridge interactions (Figure 2, left). Based on previous research from our group on gelatinase inhibitors [20,21,22], and on a work on the preference for Arg-containing ligands for salt bridge interactions at gelatinase S2 pocket [24,25], similar sulfonamido-peptidomimetics could adapt the MMP2 catalytic site, too, taking advantage of similar binding to the metal ion, hydrophobic contacts at S1′ subsite and acid-base interactions at S2 subsite (Figure 2, right). Such design allowed for conceiving a chemotype capable of displaying dual binding capacity based on a common pharmacophore between gelatinases and RGD integrins.

In this study we set out to exploit tyrosine peptidomimetics bearing sulfonamido, amino and guanidino groups as basic handles and carboxylic and hydroxamic acid groups as metal ion chelators. Therefore, we synthesized a series of molecules and assessed their binding to MMP2 and α_v_β_3_ integrin.

### 2.2. Synthesis

**Scheme 1:**
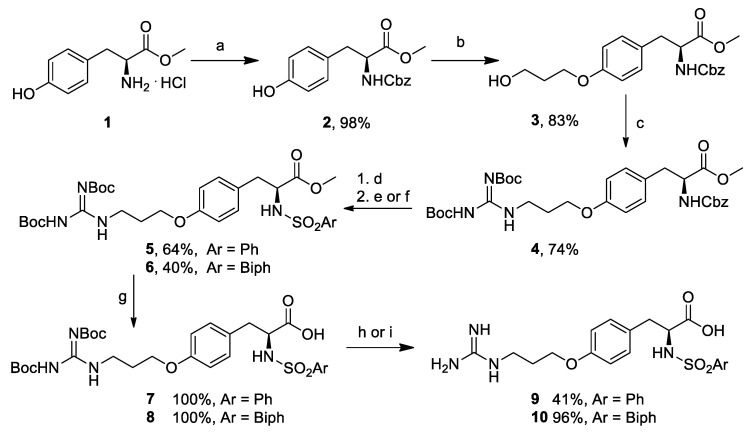
Synthesis of peptidomimetics **9** and **10.** *Reagents and conditions*: (a) CbzCl (1.5 eq.), NaHCO_3_ (2.5 eq.), H_2_O/EtOAc, 0 °C to 25 °C, 16 h; (b) PPh_3_ (1.2 eq.), DIAD (1.2 eq.), propanediol (1.2 eq.), an. THF, μW, −10 °C to 65 °C, 1 h; (c) PPh_3_ (1.1 eq.), DIAD (1.1 eq.), 1,3-bis(*tert*-butoxycarbonyl)guanidine (1.1 eq.), an. THF, μW, −10 °C to 110 °C, 30 min; (d) H_2_, Pd/C (50 mg/mmol), AcOH (cat.), MeOH, 25 °C, 16 h; (e) PhSO_2_Cl (1 eq.), DMAP (0.2 eq.), TEA (2 eq.), an. DCM, 0 °C to 25 °C, 16 h; (f) BiphSO_2_Cl (1.9 eq.), DMAP (0.2 eq.), TEA (2.9 eq.), an. DCM, 0 °C to 25 °C, 16 h; (g) 1M LiOH (2.5 mL/mmol), MeOH, 25 °C, 16 h; (h) 3M HCl (5 mL/mmol), 25 °C, 16 h; (i) 95% TFA, 5% water, 5% TES, 25 °C, 2 h.

The set of carboxylic compounds containing the guanidine moiety was synthesized as reported in Scheme 1. Boc and Cbz were selected as orthogonal protecting groups for their stability to the reaction conditions of all the steps before their removal, especially those employing strong nucleophiles as in the Mitsunobu reaction. Briefly, compound **2** was obtained quantitatively from tyrosine methyl ester (**1**) using benzyl chloroformate and then subjected to a Mitsunobu reaction to install propanediol at the tyrosine side chain, resulting in the formation of product **3** in 83% yield. Following a second Mitsunobu reaction, the side chain was further elaborated by inserting 2,3-bis-*tert*-butoxycarbonyl guanidine, to give compound **4** in 74% yield. Then, the Cbz protecting group was removed through Pd/C catalytic hydrogenation, and the free amino group was derivatized with two different arylsulfonyl chlorides, in the presence of TEA as a weak base and a catalytic quantity of DMAP, to give compounds **5** and **6** with phenyl- and biphenylsulfonyl moieties in 64 and 40% yield, respectively. Further synthetic elaboration of the two sulfonamides consisted on a basic ester hydrolysis reaction using 1M LiOH to quantitatively supplying the products **7** and **8**, followed by the removal of the Boc protective groups on the guanidine with 3M HCl or 95% TFA to furnish final peptidomimetics **9** and **10**, respectively.

**Scheme 2:**
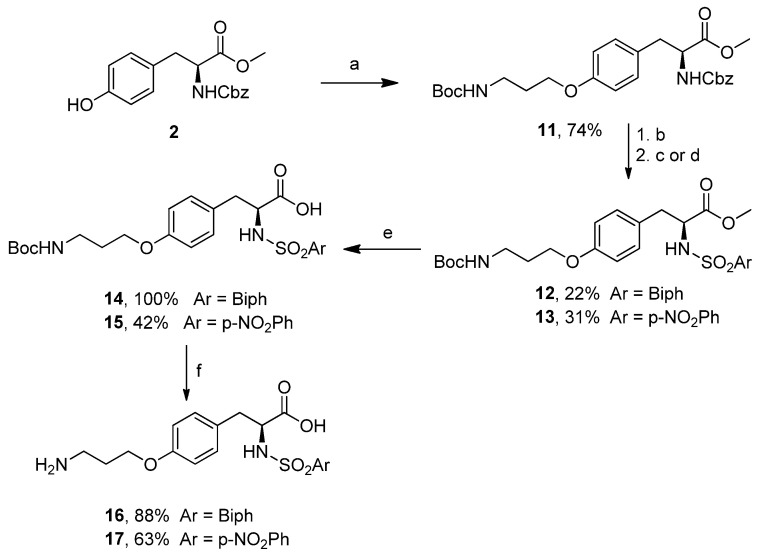
Synthesis of carboxylic acids **16** and **17.** *Reagents and conditions*: (a) PPh_3_ (1.1 eq.), DIAD (1.1 eq.), *tert*-butyl (3-hydroxypropyl)carbamate (1.1 eq.), an. THF, μW, −10 °C to 110 °C, 30 min; (b) H_2_, Pd/C (50 mg/mmol), AcOH (cat.), MeOH, 25 °C, 16 h; (c) BiphSO_2_Cl (1.1 eq.), DMAP (0.2 eq.), TEA (2.9 eq.), an. DCM, 0 °C to 25 °C, 16 h; (d) *p*NO_2_PhSO_2_Cl (1.1 eq.), DMAP (0.2 eq.), TEA (3 eq.), an. DCM, 0 °C to 25 °C, 16 h; (e) 1M LiOH (4.5 mL/mmol), MeOH, 25 °C, 16 h; (f) 3M HCl (5 mL/mmol), 25 °C, 16 h.

A subset of compounds containing the amino group in place of guanidine was synthesized as reported in Scheme 2, following a similar synthetic strategy as above. Cbz-protected tyrosine methyl ester **2** was alkylated at the side chain by means of a Mitsunobu reaction using *tert*-butyl (3-hydroxypropyl) carbamate, to give **11** in 74% yield. Subsequent Cbz deprotection by hydrogenolysis and treatment with two different arylsulfonyl chlorides, resulted in the biphenylsulfonyl derivative **12** and in the *p*-nitrosulfonyl compound **13** in 22 and 31% yield, respectively. Final removal of the ester and Boc groups under reaction conditions as above furnished the corresponding carboxylic acids **16** and **17** in quantitative yields.

**Scheme 3:**
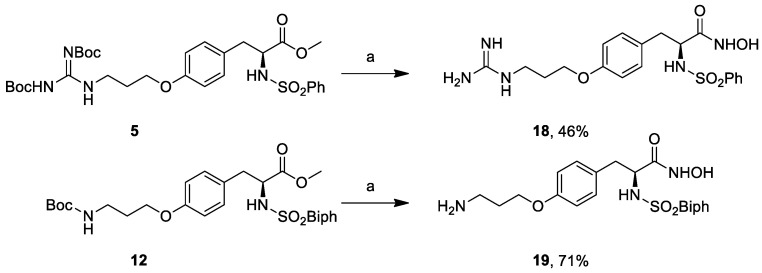
Synthesis of hydroxamic acids **18** and **19.** *Reagents and conditions*: (a) NH_2_OH^.^HCl (10 eq.) in MeOH, KOH (15 eq.) in MeOH, 25 °C, 16 h; column chromatography, then, 3M HCl, 25 °C, 16 h.

In order to achieve the two hydroxamic acids of this series, esters **5** and **12** were treated with a methanolic solution of hydroxylamine hydrochloride and KOH in MeOH at room temperature overnight (Scheme 3). Following work-up and chromatographic purification, the Boc-protected hydroxamic acids were treated with 3M HCl to furnish the final hydroxamic acids **18** and **19** in 46 and 71% yield, respectively. 

**Scheme 4:**
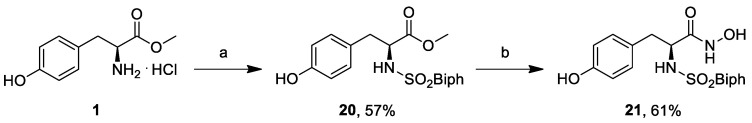
Synthesis of hydroxamic acid **21.** *Reagents and conditions*: (a) BiphSO_2_Cl (1 eq.), Na_2_CO_3_ (1 eq.), an. THF-DMF, 0 °C to 25 °C, 1 h; (b) NH_2_OH^.^HCl (10 eq.) in MeOH, KOH (15 eq.) in MeOH, 25 °C, 16 h; column chromatography, then, 3M HCl, 25 °C, 16 h.

Also, the biphenylsulfonyl-tyrosine hydroxamic acid was prepared as a control to verify the requirement of the basic handle for bioactivity towards the α_v_β_3_ integrin and MMP2. Thus, the two compounds were synthesized from Tyr-OMe (1) as shown in Scheme 4. Tyrosine ester **1** was treated with biphenylsulfonyl chloride in the presence of sodium carbonate as a base for 1h. Following work-up and chromatographic purification, a methanolic solution of hydroxylamine hydrochloride and KOH in MeOH was added to the sulfonyl derivative **20** and left reacting at room temperature overnight to achieve pure hydroxamic acid **21** after chromatographic purification. 

### 2.3. Biological Assays

The inhibition potency of the seven sulfonamido-tyrosine peptidomimetics bearing either carboxylic or hydroxamic acid and either amino or guanidino group were evaluated towards MMP2 and MMP9 through a fluorometric assay using 96-well plates and the fluorogenic substrate Mca-Lys-Pro-Leu-Gly-Leu-Dpa-Ala-Arg-NH_2_, at excitation and emission wavelengths of 320 and 420 nm, respectively. The percentage of inhibition of these compounds at 10 and 50 μM concentration is shown in Table 1. For all the compounds showing >50% inhibition activity the IC_50_ values were obtained by dose-response measurements using an inhibitor range of concentrations (0.01 nM–20 μM) and enzyme concentration equal to 1 nM.

The compounds possessing the carboxylic acid group showed poor or no inhibition towards gelatinases, as expected. Interestingly, compound **16** was proved to inhibit MMP2 with an IC_50_ in the micromolar range, demonstrating that the combination of the amino group at S2 and the biphenyl at S1′ as beneficial for inhibition for this carboxylic acid derivative. When moving to hydroxamic acids, compound **18** possessing phenylsulfonyl and guanidine groups showed reduced potency as compared to reference hydroxamic acid **21** lacking the basic appendage at Tyr side chain. On the contrary, compound **19** containing the biphenylsulfonyl moiety and an amino group instead of guanidine proved to inhibit gelatinases in the nanomolar range, possibly displaying the correct size for the S2 with respect to guanidine and showed potency similarly to reference compound **21** and with improved potency towards MMP9.

As regarding to the binding affinity towards α_v_β_3_, the library was screened using an in vitro solid-phase assays were performed at 10, 1.0 and 0.1 μM concentration of the test compounds for their ability to compete with biotinylated vitronectin for binding to pure α_v_β_3_ integrin isolated from human placenta, and % binding activity is reported in Table 2. 

**Figure 3:**
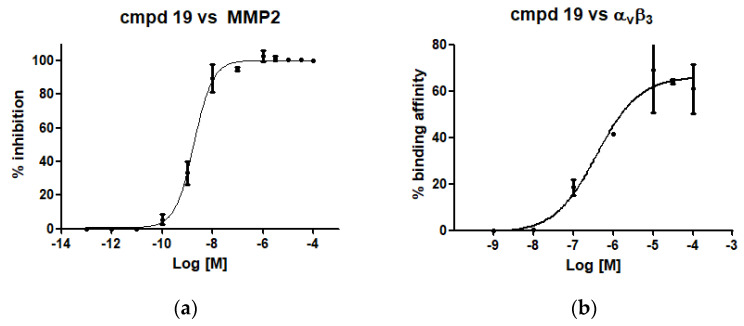
Dose-response assay curves for compound **19** vs. MMP2 ((**a**), IC_50_ = 55 nM) and α_v_β_3_ ((**b**) IC_50_ = 370 nM). IC_50_ values were calculated as the concentration of compound required for 50% inhibition of biotinylated vitronectin binding, as estimated by the GraphPad Prism software.

Preliminary structure-activity-relationship considerations were formulated starting from the binding assay results. The application of amino and guanidino groups as arginine isosteres, together with the presence of sulfonamido groups proved to impair the affinity towards the integrin, with respect to Kessler’s lead. Interestingly, the amino group maintained a binding affinity superior to guanidine. These results allowed to identify compound **19** possessing best binding potency towards α_v_β_3_, which confirmed the hydroxamic acid moiety as a good binding group for the metal ion. For this molecule a dose-response assay was carried out, allowing to determine an IC_50_ value of 370 nM (Figure 3). Taken all data together, it was possible to select compound 19 as a dual ligand candidate for both α_v_β_3_ integrin and MMP2 enzyme.

All the compounds but **17** were also tested for their integrin binding affinity towards M21 human melanoma cells that expresses high levels of α_v_β_3_ heterodimer and using cilengitide as a control [26]. Tests were performed in the presence of 2 mM MnCl_2_ to switch integrins of tumour cells into an activated form, and at final concentration of the compounds ranging from 30 μM to 0.3 μM (Table 3). All the compounds but control compound **21**, lacking the Arg isostere, showed moderate to good inhibition potency at 30 and 3 μM potency, although no dose-response profile was assessed for all the tested compounds, including cilengitide. We were pleased to find that compound **19** maintained a good inhibition potency at 0.3 μM as the lowest concentration used, thus confirming the capacity of this compound to inhibit the binding of M21 melanoma cells to vitronectin as an RGD-containing substrate.

### 2.4. Molecular Docking

**Figure 4:**
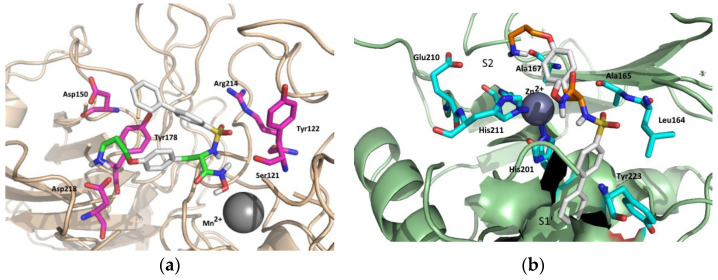
Lowest energy conformation resulting from molecular docking of compound 19 within α_v_β_3_ integrin (**a**) and MMP2 (right) binding site. Key residues of the integrin binding site are in magenta (**a**), those of MMP2 in cyan (**b**). Non polar hydrogen atoms are omitted for clarity.

Molecular docking was performed to attain an insight on the binding mode of MMP2 and α_v_β_3_ active sites. Automated docking studies were carried out using the Lamarckian Genetic Algorithm (LGA) as a search engine implemented in the Autodock 4.0.1 program. Coordinates of compound **19** were generated using Spartan (version 5.147), and then energy-minimized through the AM1 semi-empirical method to calculate the minimum-energy conformer. The coordinates of α_v_β_3_ and MMP2 were retrieved from the Protein Data Bank (PDB codes: 1L5G and 1QIB for α_v_β_3_ and MMP2, respectively), and protein-ligand complexes were unmerged for achieving the free protein structures. For α_v_β_3_, best docking pose of compound **19** showed the canonical binding mode consisting of key polar and hydrophobic interactions (Figure 4a). Specifically, the hydroxamic acid moiety was found interacting with Mn^2+^ ion and the amino group exhibiting hydrogen-bonding interactions with Asp218, as expected. The two aryl moieties of **19** established π-stacking interactions with Tyr178 and Ty122, and additional polar contacts were observed between the sulfonamide and Arg214 side chain. When considering the interactions within MMP2 active site, the hydroxamic acid group was found interacting with Zn^2+^ ion and the sulfonamide displaying hydrogen-bonds with Ala165 and Leu164, as expected (Figure 4b). The biphenyl group was found within S1′ as a major driver for molecular recognition, whereas the aryl ring of tyrosine side chain was found interacting with His211. Interestingly, the amino group was found establishing hydrogen-bonding interactions with Ala167 and close to Glu210 in the S2 region, as hypothesized. Thus, the positioning of 19 within the binding regions of the two proteins confirmed the hypothesized existence of a similar pharmacophore. 

## 3. Materials and Methods

### 3.1. General

Analytical grade solvents and commercially available reagents were used without further purification. Reactions requiring an inert atmosphere were carried out under a nitrogen atmosphere. ^1^H-NMR and ^13^C-NMR spectra were recorded on a Mercury 400 (^1^H: 400 MHz, ^13^C: 100 MHz), or a Mercury 200 (^1^H: 200 MHz, ^13^C: 50 MHz) spectrometer (Varian, Palo Alto, CA, USA). The chemical shifts (*δ*) and coupling constants (*J*) are expressed in parts per million (ppm) and Hertz (Hz), respectively. Copies of ^1^H- and ^13^C-NMR spectra of compounds **3–21** are provided as Supplementary Material. Flash column chromatography (FCC) purifications were performed manually using glass columns filled with silica gel (40–63 μm, Merck KGaA, Darmstadt, Germany) or using an Isolera automatic system (Biotage Sweden AB, Uppsala, Sweden) and SNAP silica cartridges. TLC analyses were performed on Merck silica gel 60 F254 plates. Optical rotations were measured with DIP-360 digital polarimeter (JASCO, Easton, MD, USA). ESI-MS spectra were recorded on a LCQ Fleet ion-trap double quadrupole mass spectrometer (Thermo Scientific, Waltham, MA, USA) using electrospray (ES^+^) ionization techniques. Dichloromethane (DCM) was dried by distillation over CaH2 and THF was dried by distillation over Na/benzophenone. Reactions conducted with microwave irradiation were performed with an automatic single-mode Biotage Initiator Sixty microwave synthesizer (Biotage Sweden AB, Uppsala, Sweden) equipped with temperature and pressure monitoring sensors, using sealed reaction tubes.

### 3.2. (S)-Methyl 2-(((Benzyloxy)carbonyl)amino)-3-(4-hydroxyphenyl)propanoate *(**2**)*

To a 250 mL round bottom flask containing a solution of L-Tyr-OMe^.^HCl (1) (3.0 g, 12.9 mmol) in EtOAc (28.0 mL), a solution of NaHCO_3_ (2.7 g, 32.0 mmol) in H_2_O (30.0 mL) was added. Benzyl chloroformate (3.30 g, 19.4 mmol) was then added dropwise at 0 °C, and the resulting reaction mixture was warmed to room temperature and stirred overnight. After a TLC check (PE/EtOAc 2.5:1, R_f_ = 0.53), the organic phase was diluted with 80 mL of a mixture of EtOAc/H_2_O (1:1). The resulting organic phase was separated, washed with H_2_O and brine, dried over Na_2_SO_4_, and concentrated in vacuo. The title compound was obtained as a grainy white solid (4.20 g, 98% yield), that was used without further purification. Spectroscopic data were in agreement with those reported in the literature [27].

### 3.3. (S)-Methyl 2-(((Benzyloxy)carbonyl)amino)-3-(4-(3-hydroxypropoxy)phenyl)propanoate *(**3**)*

Under an atmosphere of N_2_, a 250 mL round bottom flask was charged with PPh_3_ (954 mg, 3.64 mmol) followed by compound **2** (1.0 g, 3.04 mmol) in dry THF (30 mL). DIAD (716 μL, 3.64 mmol) was added dropwise at −10 °C, and the reaction mixture was stirred for 10 min. Propanediol (263 μL, 3.64 mmol) was added dropwise at −10 °C. The reaction solution was warmed to room temperature and reacted for 1 h at 65 °C under microwave irradiation. The solvent was removed under reduced pressure, and the residue was purified by Biotage Isolera system to provide the title compound as a colorless oil (960 mg, 2.48 mmol, 83% yield). ${\left[\alpha \right]}_{D}^{22}$ = 27.2 (c 1.0, CHCl_3_). ^1^H-NMR (400 MHz, CDCl_3_) δ (ppm) = 7.35–7.31 (m, 5H), 6.99 (d, *J* = 8.5 Hz, 2H), 6.80 (d, *J* = 8.5 Hz, 2H), 5.23 (d, *J* = 7.9 Hz, 1H), 5.09 (s, 2H), 4.61 (dd, *J* = 13.3, 5.8 Hz, 1H), 4.08 (t, *J* = 5.9 Hz, 2H), 3.85 (t, *J* = 5.4 Hz, 2H), 3.71 (s, 3H), 3.04 (dd, *J* = 8.6, 6.2 Hz, 2H), 2.08–1.98 (m, 2H). ^13^C-NMR (100 MHz, CDCl_3_) δ 172.0, 157.9, 155.6, 136.2, 131.9, 130.2, 128.5, 128.1, 128.0, 127.8, 114.6, 66.9, 65.6, 60.4, 54.9, 52.3, 37.3, 32.0. MS (ESI) *m*/*z* (%) = 410.25 (100, [M + Na]^+^).

### 3.4. (S)-Methyl 2-(((Benzyloxy)carbonyl)amino)-3-(4-(3-(2,3-bis(tert-butoxycarbonyl)guanidino)propoxy)phenyl)propanoate *(**4**)*

Under an atmosphere of N_2_, a 100 mL round bottom flask was charged with PPh_3_ (776 mg, 2.96 mmol) and compound **3** (1.04 g, 2.69 mmol) in dry THF (27 mL). DIAD (582 μL, 2.96 mmol) was added dropwise at −10 °C, and the reaction mixture was stirred for 10 min. A solution of bis-*N*-Boc guanidine (766 mg, 2.96 mmol) in dry THF (2 mL) was added dropwise at −10 °C. The reaction solution was warmed to room temperature, divided between two 20 mL microwave vials, which were reacted for 30 min at 110 °C under microwave irradiation. After TLC check (PE/EtOAc 2:1, R_f_ = 0.44), the solvent was removed under reduced pressure. The reaction was purified by Biotage Isolera system to provide the title compound as a colorless oil (1.25 g, 2.69 mmol, 74% yield). ${\left[\alpha \right]}_{D}^{23}$ = 27.9 (c 1.0, CHCl_3_). ^1^H- NMR (400 MHz, CDCl_3_) δ 9.30 (br s, 2H), 7.42–7.26 (m, 5H), 6.97 (d, *J* = 8.5 Hz, 2H), 6.76 (d, *J* = 8.5 Hz, 2H), 5.21 (d, *J* = 7.9 Hz, 1H), 5.09 (s, 2H), 4.60 (dd, *J* = 13.3, 5.7 Hz, 1H), 4.10 (t, *J* = 6.9 Hz, 2H), 3.97 (t, *J* = 6.1 Hz, 2H), 3.70 (s, 3H), 3.10–2.98 (m, 2H), 2.06 (dd, *J* = 14.2, 7.6 Hz, 2H), 1.48 (s, 18H). ^13^C-NMR (100 MHz, CDCl_3_) δ 172.0, 163.8, 160.6, 158.1, 155.6, 155.0, 136.2, 132.1, 130.2, 128.5, 128.2, 128.1, 127.5, 114.4, 83.8, 78.7, 66.9, 65.6, 54.9, 52.3, 42.3, 37.3, 28.7, 28.3, 28.0. MS (ESI) *m*/*z* (%) = 629.01 (100, [M + H]^+^). 

### 3.5. (S)-Methyl 3-(4-(3-(2,3-bis(tert-Butoxycarbonyl)guanidino)propoxy)phenyl)-2-(phenylsulfonamido)propanoate *(**5**)*

To a two neck 25 mL round bottom flask a solution of compound **4** (950 mg, 1.5 mmol) in MeOH (7.5 mL), Pd/C (75 mg), and catalytic AcOH were added. H_2_ gas was let to stream inside the flask for 15 min, and the reaction solution was overnight stirred at room temperature under a H_2_ atmosphere. After TLC check (EtOAc/PE 1:3, R_f_ = 0.0), the reaction solution was filtered over Celite, and concentrated in vacuo. The so obtained free amine (720 mg, 1.5 mmol, quantitative yield) was used in the next synthetic step without further purification. ^1^H-NMR (200 MHz, CDCl_3_) δ (ppm) = 9.40 (br s, 2H), 7.13 (d, *J* = 10 Hz, 2H), 6.87 (d, *J* = 8 Hz, 2H), 4.17 (t, *J* = 6 Hz, 2H), 4.05 (t, *J* = 6 Hz, 1H), 3.78 (s, 3H), 3.13 (d, *J* = 6 Hz, 2H), 2.14 (t, *J* = 6 Hz, 2H), 1.56 (s, 18H). In a 10 mL round bottom flask containing a solution of the free amine (100 mg, 0.2 mmol) and DMAP (5 mg, 0.04 mmol) in dry DCM (2 mL), TEA (56 μL, 0.4 mmol) and then phenylsulfonyl chloride (28 μL, 0.2 mmol) were added dropwise at 0 °C. The reaction solution was stirred at room temperature overnight, and after TLC check (EtOAc/PE 1:2, R_f_ = 0.23), it was washed with 1M HCl, satd. NaHCO_3_ and Brine. The organic phase was dried over Na_2_SO_4,_ and the solvent was removed under reduced pressure. The crude was purified by FCC (Et_2_O/PE 1:1) to provide the title compound (81 mg, 0.1 mmol, 64% yield). ${\left[\alpha \right]}_{D}^{23}$ = 1.4 (c 0.4, CHCl_3_). ^1^H-NMR (400 MHz, CDCl_3_) δ (ppm) = 9.26 (br s, 2H), 7.76 (d, *J* = 7.4 Hz, 2H), 7.54 (t, *J* = 7.4 Hz, 1H), 7.45 (t, *J* = 7.7 Hz, 2H), 6.95 (d, *J* = 8.5 Hz, 2H), 6.73 (d, *J* = 8.5 Hz, 2H), 5.06 (d, *J* = 8.9 Hz, 1H), 4.18 (dd, *J*_1_ = 14.5 Hz, *J*_2_ = 5.8 Hz, 1H), 4.10 (t, *J* = 7.0 Hz, 2H), 3.97 (t, *J* = 6.2 Hz, 2H), 3.46 (s, 3H), 2.97 (d, *J* = 5.8 Hz, 2H), 2.17–1.98 (m, 2H), 1.50 (s, 9H), 1.48 (s, 9H). ^13^C-NMR (100 MHz, CDCl_3_) δ (ppm) = 171.1, 163.8, 160.6, 158.2, 155.0, 139.6, 132.7, 130.3, 128.9, 127.1, 126.6, 114.4, 83.8, 78.7, 65.6, 56.7, 52.3, 42.2, 38.5, 28.7, 28.3, 28.0. MS (ESI) *m*/*z* (%) = 657.12 (100, [M + Na]^+^). 

### 3.6. (S)-Methyl 2-([1,1′-Biphenyl]-4-ylsulfonamido)-3-(4-(3-(2,3-bis(tert-butoxycarbonyl)guanidino)propoxy)phenyl)propanoate *(**6**)*

To a 50 mL round bottom flask containing a solution of the free amine obtained from **4** as in Section 3.5 (720 mg, 1.5 mmol), DMAP (40 mg, 0.3 mmol) in dry DCM (15 mL), and TEA (606 μL, 4.4 mmol), biphenylsulfonyl chloride (731 mg, 2.9 mmol) was added dropwise at 0 °C. The solution was stirred at room temperature overnight, and after TLC check (EtOAc/PE 1:3, R_f_ = 0.50), it was washed with 1M HCl, satd. NaHCO_3_ and brine. The organic phase was dried over Na_2_SO_4_ and concentrated in vacuo. The crude was purified by FCC (Et_2_O/PE 2:3) to provide the title compound (412 mg, 0.6 mmol, 40% yield). ${\left[\alpha \right]}_{D}^{24}$ = −42.2 (c 0.7, CHCl_3_). ^1^H-NMR (200 MHz, CDCl_3_) δ 9.28 (s, 2H), 7.80 (d, *J* = 8.1 Hz, 2H), 7.72–7.54 (m, 5H), 7.46 (d, *J* = 7.3 Hz, 3H), 6.96 (d, *J* = 8.0 Hz, 2H), 6.73 (d, *J* = 8.1 Hz, 2H), 5.09 (d, *J* = 8.9 Hz, 1H), 4.20 (d, *J* = 8.8 Hz, 1H), 4.08 (t, *J* = 6.5 Hz, 2H), 3.94 (d, *J* = 5.5 Hz, 2H), 3.48 (s, 3H), 2.99 (d, *J* = 5.2 Hz, 2H), 2.06 (d, *J* = 6.0 Hz, 2H), 1.50 (s, 9H), 1.49 (s, 9H). ^13^C-NMR (50 MHz, CDCl_3_) δ (ppm) = 171.3, 163.8, 160.7, 158.3, 155.0, 145.7, 139.2, 138.3, 130.4, 129.1, 128.6, 127.7, 127.6, 127.3, 114.5, 83.9, 78.7, 65.8, 56.9, 52.4, 42.3, 38.6, 28.8, 28.4, 28.1. MS (ESI) *m*/*z* (%) = 733.92 (100, [M + Na]^+^). 

### 3.7. (S)-3-(4-(3-(2,3-bis(tert-Butoxycarbonyl)guanidino)propoxy)phenyl)-2-(phenylsulfonamido)propanoic Acid *(**7**)*

In a 5 mL round bottom flask containing a solution of compound **5** (50 mg, 0.08 mmol) in MeOH (0.6 mL), 1M LiOH (0.2 mL) was added and stirred at room temperature overnight. The solution was then diluted with EtOAc, acidified to pH 1 with 1N HCl and extracted with EtOAc. The organic phase was dried over Na_2_SO_4_, and the solvent was removed under reduced pressure to provide the title compound (48 mg, 0.08 mmol, quantitative yield) which was used in the next synthetic step without further purification. ${\left[\alpha \right]}_{D}^{22}$ = 10.3 (c 0.9, CHCl_3_). ^1^H-NMR (400 MHz, CDCl_3_) δ (ppm) = 9.03 (s, 1H), 7.77 (d, *J* = 7.6 Hz, 2H), 7.57–7.38 (m, 3H), 7.05 (d, *J* = 8.0 Hz, 2H), 6.72 (d, *J* = 7.8 Hz, 2H), 5.64 (br s, 1H), 4.12 (br s, 1H), 3.94 (br s, 2H), 3.44 (d, *J* = 13.5, 2H), 3.08 (dd, *J*_1_ =19.2 Hz, *J*_2_ = 14.5 Hz, 1H), 2.99 (d, *J* = 4.3 Hz, 2H), 2.00 (br s, 2H), 1.51 (s, 18H). ^13^C-NMR (100 MHz, CDCl_3_) δ (ppm) = 173.0, 157.3, 154.3, 152.3, 132.8, 132.2, 131.0, 129.1, 127.0, 114.4, 85.3, 64.9, 56.8, 46.0, 39.3, 29.7, 27.9. MS (ESI) *m*/*z* (%) = 619.09 (100, [M − H]^−^). 

### 3.8. (S)-2-([1,1′-Biphenyl]-4-ylsulfonamido)-3-(4-(3-(2,3-bis(tert-butoxycarbonyl)guanidino)propoxy)phenyl)propanoic Acid *(**8**)*

In a 5 mL round bottom flask containing a solution of compound **6** (200 mg, 0.28 mmol) in MeOH (1.0 mL), 1M LiOH (1.0 mL) was added and stirred at room temperature overnight. The reaction solution was then diluted with EtOAc, acidified to pH 1 with 1N HCl and extracted with EtOAc. The organic phase was dried over Na_2_SO_4_ and concentrated *in vacuo* to provide the title compound (195 mg, 0.28 mmol, quantitative yield) which was used in the next step without further purification. ${\left[\alpha \right]}_{D}^{23}$ = −9.5 (c 1.0, MeOH). ^1^H-NMR (200 MHz, CD_3_OD) δ 7.79–7.44 (m, 9H), 7.11 (d, *J* = 8 Hz, 2H), 6.81 (d, *J* = 8 Hz, 2H), 4.14 (s, 1H), 3.99 (s, 2H), 3.75 (d, *J* = 4 Hz, 1H), 3.25–2.68 (m, 4H), 2.05 (s, 2H), 1.60 (s, 9H). ^13^C-NMR (100 MHz, CDCl_3_) δ 166.0, 155.3, 145.9, 136.5, 131.7, 129.3, 128.8, 128.4, 128.3, 127.5, 122.2, 87.8, 84.8, 62.1, 58.0, 57.3, 54.6, 45.2, 30.8, 21.9. MS (ESI) *m*/*z* (%) = 695.73 (100, [M − H]^−^). 

### 3.9. (S)-3-(4-(3-Guanidinopropoxy)phenyl)-2-(phenylsulfonamido)propanoic Acid *(**9**)*

In a 5 mL round bottom flask, compound **7** (19 mg, 0.03 mmol) was dissolved in 3M HCl (150 μL). The reaction solution was stirred at room temperature overnight. After TLC check (EtOAc, R_f_ = 0.06), the solvent was removed under reduced pressure to provide the title compound (56 mg, 0.13 mmol, 41% yield). ${\left[\alpha \right]}_{D}^{22}$ = 14.2 (c 0.5, H_2_O). ^1^H-NMR (400 MHz, DMSO) δ (ppm) = 8.22 (d, *J* = 9.0 Hz, 1H), 7.82 (br s, 1H), 7.64–7.34 (m, 5H), 7.01 (d, *J* = 8.6 Hz, 2H), 6.74 (dd, *J*_1_ = 12.0 Hz, *J*_2_ = 5.3 Hz, 2H), 3.97 (m, 2H), 3.80 (td, *J*_1_ = 8.9 Hz, *J*_2_ = 5.7 Hz, 1H), 3.35-3.32 (m, 1H), 3.04 (d, *J* = 3.5 Hz, 1H), 2.84 (dt, *J*_1_ = 15.8 Hz, *J*_2_ = 7.9 Hz, 1H), 2.61 (dd, *J*_1_ = 13.7 Hz, *J*_2_ = 9.1 Hz, 1H), 2.03–1.83 (m, 2H). ^13^C-NMR (100 MHz, DMSO) δ (ppm) = 172.7, 158.2, 157.5, 141.5, 132.5, 132.3, 130.6, 129.2, 126.6, 114.6, 64.7, 58.1, 38.1, 28.7, 28.0. MS (ESI) *m*/*z* (%) = 443.21 (100, [M + Na]^+^). 

### 3.10. (S)-2-([1,1′-Biphenyl]-4-ylsulfonamido)-3-(4-(3-guanidinopropoxy)phenyl)propanoic Acid *(**10**)*

In a 5 mL round bottom flask, compound **8** (33 mg, 0.05 mmol) was dissolved in 2 mL of a mixture of TFA (95%), water (2.5%) and TES (2.5%). The reaction solution was stirred at room temperature for 2 h. After TLC check (EtOAc, R_f_ = 0.0), the solvent was removed under reduced pressure to provide the title compound (24 mg, 0.05 mmol, 96% yield). ${\left[\alpha \right]}_{D}^{23}$ = −27.1 (c 1.0, MeOH). ^1^H-NMR (400 MHz, CD_3_OD) δ 7.70–7.58 (m, 6H), 7.48 (t, *J* = 7.5 Hz, 2H), 7.40 (t, *J* = 7.3 Hz, 1H), 7.02 (d, *J* = 8.5 Hz, 2H), 6.71 (d, *J* = 8.5 Hz, 2H), 4.00 (dd, *J* = 9.1, 5.0 Hz, 1H), 3.86 (tt, *J* = 9.1, 4.6 Hz, 2H), 3.28 (d, *J* = 6.9 Hz, 2H), 3.01 (dd, *J* = 13.8, 4.9 Hz, 1H), 2.75 (dd, *J* = 13.8, 9.2 Hz, 1H), 1.95–1.84 (m, 2H). ^13^C-NMR (100 MHz, CD_3_OD) δ (ppm) = 174.7, 159.0, 158.7, 146.2, 140.8, 140.7, 131.4, 130.3, 130.1, 129.4, 128.5, 128.3, 115.3, 65.8, 59.2, 39.6, 39.0, 29.6. MS (ESI) *m*/*z* (%) = 519.36 (100, [M+Na]^+^). 

### 3.11. (S)-Methyl 2-(((Benzyloxy)carbonyl)amino)-3-(4-(3-((tert-butoxycarbonyl)amino)propoxy)phenyl)propanoate *(**11**)*

Under an atmosphere of N_2_, a 250 mL round bottom flask was charged with PPh_3_ (2.8 g, 10.6 mmol) and a solution of compound **2** (3.2 g, 9.7 mmol) in dry THF (90 mL). DIAD (2.1 g, 10.6 mmol) was then added dropwise at −10 °C, and the reaction mixture was stirred for 10 min. A solution of *tert*-butyl (3-hydroxypropyl) carbamate (1.6 g, 10.6 mmol) in dry THF (4 mL) was added dropwise at −10 °C. The reaction solution was warmed to room temperature, partitioned between 5 microwave 20 mL vials, and reacted at 110 °C for 30 min under microwave irradiation. After TLC check (PE/EtOAc 2:1, R_f_ = 0.19), the solvent was removed under reduced pressure, and the residue was purified by FCC (Et_2_O/PE 2:1) to provide the title compound as a colorless oil (3.5 g, 7.1 mmol, 74% yield). ${\left[\alpha \right]}_{D}^{22}$ = 39.6 (c 1.0, CHCl_3_). ^1^H-NMR (200 MHz, CDCl_3_) δ (ppm) = 7.34 (s, 5H), 6.98 (d, *J* = 4 Hz, 2H), 6.78 (d, *J* = 4 Hz, 2H), 5.22 (d, *J* = 4 Hz, 1H), 5.09 (s, 2H), 4.79 (m, 1H), 4.66-4.56 (m, 1H), 3.97 (t, *J* = 6 Hz, 2H), 3.72 (s, 3H), 3.32 (q, *J* = 6 Hz, 2H), 3.05–3.03 (m, 2H), 1.96 (quint, *J* = 5.5 Hz, 2H), 1.43 (s, 9H). ^13^C-NMR (50 MHz, CDCl_3_) δ (ppm) = 172.1, 157.9, 156.1, 155.7, 136.3, 130.2, 128.5, 128.1, 128.0, 127.8, 115.6, 114.6, 79.2, 66.9, 65.7, 55.0, 52.3, 38.0, 37.3, 29.5, 28.4. MS (ESI) *m*/*z* (%) = 509.04 (100, [M + Na]^+^). 

### 3.12. (S)-Methyl 2-([1,1′-Biphenyl]-4-ylsulfonamido)-3-(4-(3-((tert-butoxycarbonyl)amino)propoxy)phenyl)propanoate *(**12**)*

To a 100 mL round bottom two neck flask a solution of compound **11** (3.3 g, 6.9 mmol) in MeOH (34 mL), Pd/C (340 mg), and catalytic AcOH were added. H_2_ gas was let to stream inside the flask for 15 min. The reaction solution was then stirred at room temperature, under H_2_ atmosphere, overnight. The reaction solution was filtered over Celite, and the solvent was removed under reduced pressure. The obtained free amine (2.2 g, 6.4 mmol, 93% yield) was used in the next step without further purification. ^1^H-NMR (200 MHz, CDCl_3_) δ (ppm) = 7.30 (d, *J* = 7 Hz, 2H), 6.82 (d, *J* = 5 Hz, 2H), 4.83 (m, 1H), 3.97 (t, *J* = 3 Hz, 2H), 3.71 (s, 3H), 3.30 (m, 2H), 3.10–3.00 (m, 1H), 2.90-2.80 (m, 1H), 2.59 (br s, 2H), 1.95 (m, 2H), 1.42 (s, 9H). In a 100 mL round bottom flask containing a solution of the free amine (1.1 g, 3.2 mmol), DMAP (78 mg, 0.64 mmol) in dry DCM (32 mL), and TEA (1.33 mL, 9.5 mmol) biphenylsulfonyl chloride (882 mg, 3.5 mmol) was added dropwise at 0 °C. The reaction solution was stirred at room temperature overnight, and after TLC check (Et_2_O/PE 2:1, R_f_ = 0.34), it was washed with 1M HCl, satd. NaHCO_3_ and brine. The organic phase was then dried over Na_2_SO_4_ and concentrated *in vacuo*. The crude was purified by FCC (Et_2_O/PE 1:1) to provide the title compound (320 mg, 0.56 mmol, 22% yield). ${\left[\alpha \right]}_{D}^{23}$ = 1.3 (c 2.0, CHCl_3_). ^1^H-NMR (200 MHz, CDCl_3_) δ (ppm) = 7.80–7.41 (m, 9H), 6.97 (d, *J* = 4 Hz, 2H), 6.74 (d, *J* = 4 Hz, 2H), 5.07 (d, *J* = 6 Hz, 1H), 4.72 (br, 1H), 4.21–4.17 (m, 1H), 3.91 (t, *J* = 3 Hz, 2H), 3.50 (s, 3H), 3.28 (m, 2H), 3.04-2.93 (m, 2H), 1.92 (m, 2H), 1.44 (s, 9H). ^13^C-NMR (100 MHz, CDCl_3_) δ (ppm) = 171.3, 157.9, 155.9, 145.3, 139.1, 138.2, 132.0, 130.3, 129.0, 128.4, 127.4, 127.1, 114.4, 79.0, 65.6, 56.9, 52.3, 38.3, 37.9, 29.4, 28.3. MS (ESI) *m*/*z* (%) = 591.03 (100, [M + Na]^+^). 

### 3.13. (S)-Methyl 3-(4-(3-((tert-Butoxycarbonyl)amino)propoxy)phenyl)-2-(4-nitrophenylsulfonamido)propanoate *(**13**)*

In a 50 mL round bottom flask containing a solution of free amine (1.1 g, 3.2 mmol), prepared from 11 as in (12), DMAP (78 mg, 0.64 mmol) in dry DCM (32 mL), and TEA (1.3 mL, 9.5 mmol) 4-nitrophenylsulfonyl chloride (773 mg, 3.5 mmol) was added dropwise at 0 °C. The reaction solution was stirred at room temperature overnight. After TLC check (EtOAc/PE 2:3, R_f_ = 0.75), it was washed with 1M HCl, satd. NaHCO_3_ and brine. The organic phase was dried over Na_2_SO_4_ and concentrated in vacuo. The crude was purified by FCC (Et_2_O/PE (3:2)) to provide the title compound (530 mg, 1.0 mmol, 31% yield). ${\left[\alpha \right]}_{D}^{23}$ = −6.6 (c 1.0, CHCl_3_). ^1^H-NMR (400 MHz, CDCl_3_) δ 8.22 (d, *J* = 8.8 Hz, 2H), 7.84 (d, *J* = 8.8 Hz, 2H), 6.93 (d, *J* = 8.5 Hz, 2H), 6.71 (d, *J* = 8.5 Hz, 2H), 5.27 (d, *J* = 9.3 Hz, 1H), 4.77 (br s, 1H), 4.21 (ddd, *J* = 9.3, 7.1, 5.2 Hz, 1H), 3.94 (t, *J* = 6.0 Hz, 2H), 3.62 (s, 3H), 3.32 (d, *J* = 6.3 Hz, 2H), 3.05 (dd, *J* = 14.0, 5.1 Hz, 1H), 2.90 (dd, *J* = 14.0, 7.2 Hz, 1H), 2.01–1.90 (m, 2H), 1.44 (s, 9H). ^13^C-NMR (100 MHz, CDCl_3_) δ 171.1, 158.2, 149.8, 145.6, 130.4, 128.2, 126.7, 124.1, 114.6, 110.5, 110.0, 108.5, 65.7, 57.2, 52.8, 38.4, 28.4. MS (ESI) *m*/*z* (%) = 559.99 (100, [M + Na]^+^). 

### 3.14. (S)-2-([1,1′-Biphenyl]-4-ylsulfonamido)-3-(4-(3-((tert-butoxycarbonyl)amino)propoxy)phenyl)propanoic Acid *(**14**)*

In a 5 mL round bottom flask containing the solution of compound **12** (74 mg, 0.13 mmol) in MeOH (580 μL), 1M LiOH (580 μL) was added and stirred at room temperature overnight. The reaction solution was then diluted with EtOAc, acidified to pH 1 with 1N HCl and extracted with further EtOAc. The organic phase was dried over Na_2_SO_4_ and concentrated in vacuo to provide the title compound (72 mg, 0.13 mmol, quantitative yield) which was used in the next step without further purification. ${\left[\alpha \right]}_{D}^{23}$ = −4.0 (c 1.0, CHCl_3_). ^1^H-NMR (200 MHz, CDCl_3_) δ (ppm) = 7.77–7.33 (m, 9H), 7.01 (d, *J* = 8 Hz, 2H), 6.70 (d, *J* = 8 Hz, 2H), 5.36 (d, *J* = 8 Hz, 1H), 4.24–4.10 (m, 1H), 3.85 (m, 2H), 3.22–3.02 (m, 4H), 1.85 (m, 2H), 1.42 (s, 9H). ^13^C-NMR (50 MHz, CDCl_3_) δ (ppm) = 157.9, 145.4, 139.2, 138.1, 130.6, 129.0, 128.5, 127.6, 127.5, 127.3, 114.4, 77.2, 65.6, 56.9, 38.0, 29.7, 29.4, 28.4. MS (ESI) *m*/*z* (%) = 553.04 (100, [M − H]^−^). 

### 3.15. (S)-3-(4-(3-((tert-Butoxycarbonyl)amino)propoxy)phenyl)-2-(4-nitrophenylsulfonamido)propanoic Acid *(**15**)*

In a 5 mL round bottom flask containing the solution of compound **13** (209 mg, 0.38 mmol) in MeOH (1.0 mL), an aqueous solution of 1M LiOH (1.0 mL) was added and stirred at room temperature overnight. The reaction solution was then diluted with EtOAc, acidified to pH 1 with 1N HCl and extracted with further EtOAc. The organic phase was dried over Na_2_SO_4_ and concentrated in vacuo to provide the title compound (83 mg, 0.16 mmol, 42% yield) which was used the next synthetic step without further purification. ${\left[\alpha \right]}_{D}^{23}$ = −15.8 (c 0.95, CHCl_3_). ^1^H-NMR (200 MHz, CDCl_3_) δ (ppm) = 8.14 (d, *J* = 8 Hz, 2H), 7.78 (m, 2H), 6.96 (d, *J* = 8 Hz, 2H), 6.64 (d, *J* = 10 Hz, 2H), 5.76 (m, 1H), 4.15 (m, 1H), 3.88 (m, 1H), 3.26 (m, 2H), 2.89 (m, 1H), 1.89 (m, 2H), 1.43 (s, 9H). ^13^C-NMR (50 MHz, CDCl_3_) δ (ppm) = 174.3, 158.0, 149.7, 145.6, 130.5, 128.1, 127.3, 124.0, 114.5, 65.6, 57.4, 53.4, 37.9, 29.4, 28.3. MS (ESI) *m*/*z* (%) = 545.95 (100, [M + H]^+^).

### 3.16. (S)-2-([1,1′-Biphenyl]-4-ylsulfonamido)-3-(4-(3-aminopropoxy)phenyl)propanoic Acid *(**16**)*

In a 5 mL round bottom flask, compound **14** (44 mg, 0.08 mmol) was dissolved in a solution of 3M HCl in 1,4-dioxane (400 μL). The reaction solution was stirred at room temperature overnight. After TLC check (DCM/MeOH (5:1), R_f_ = 0.0), the solvent was removed under reduced pressure to provide the title compound (34 mg, 0.07 mmol, 88% yield). ${\left[\alpha \right]}_{D}^{23}$ = −13.7 (c 1.0, MeOH). ^1^H-NMR (400 MHz, CD_3_OD) δ (ppm) = 7.60 (s, 6H), 7.47–7.36 (m, 3H), 6.98 (d, *J* = 8 Hz, 2H), 6.67 (d, *J* = 8 Hz, 2H), 3.92–3.85 (m, 3H), 3.60-3.54 (m, 1H), 2.97–2.93 (m, 3H), 2.74–2.63 (m, 1H), 1.95 (m, 2H). ^13^C-NMR (101 MHz, CDCl_3_) δ (ppm) = 164.0, 157.4, 144.8, 139.3, 139.2, 130.0, 129.0, 128.8, 128.1, 127.8, 127.1, 126.9, 113.9, 64.6, 37.6, 37.2, 29.3, 27.0. MS (ESI) *m*/*z* (%) = 553.17 (100, [M − H]^−^). 

### 3.17. (S)-3-(4-(3-Aminopropoxy)phenyl)-2-(4-nitrophenylsulfonamido)propanoic Acid *(**17**)*

In a 5 mL round bottom flask, compound 15 (80 mg, 0.15 mmol) was dissolved in 3M HCl (800 μL). The reaction solution was stirred at room temperature overnight. After TLC check (EtOAc, R_f_ = 0.0), the solvent was removed under reduced pressure to provide the title compound (43 mg, 0.09 mmol, 63% yield). ${\left[\alpha \right]}_{D}^{23}$ = −7.8 (c 1.0, MeOH). ^1^H-NMR (200 MHz, D_2_O) δ (ppm) = 7.89 (d, *J* = 10 Hz, 2H), 7.40 (d, *J* = 8 Hz, 2H), 6.70 (d, *J* = 10 Hz, 2H), 6.35 (d, *J* = 8 Hz, 2H), 3.92–3.74 (m, 4H), 3.03–2.87 (m, 4H), 2.47–2.34 (m, 1H), 1.91 (m, 2H). ^13^C-NMR (50 MHz, CD_3_OD) δ (ppm) = 171.8, 157.4, 149.3, 146.8, 130.1, 129.0, 127.7, 123.8, 114.0, 65.1, 58.2, 37.7, 37.3, 27.2. MS (ESI) *m*/*z* (%) = 424.04 (100, [M + H]^+^). 

### 3.18. (S)-3-(4-(3-Guanidinopropoxy)phenyl)-N-hydroxy-2-(phenylsulfonamido)propanamide *(**18**)*

NH_2_OH^.^HCl (70 mg, 1.01 mmol) was solubilized in MeOH (0.4 mL) by heating to reflux until most of the salt was dissolved. The solution was cooled to <40 °C, and a solution of KOH (84 mg, 1.51 mmol) in MeOH (0.2 mL) was added in one portion. The resulting suspension was cooled to room temperature and was added without prior removal of precipitated material to compound **5** (80 mg, 0.13 mmol) and stirred at room temperature for 16 h. The reaction mixture was taken up in 1M HCl, extracted with EtOAc, dried over anhydrous Na_2_SO_4_, filtered, and concentrated *in vacuo*. The residue was purified by FCC (EtOAc) to provide the pure compound that was subsequently dissolved in 3M HCl (300 μL). The reaction solution was stirred at room temperature overnight and concentrated under reduced pressure to give the title compound (26 mg, 0.06 mmol, 46% yield). ${\left[\alpha \right]}_{D}^{24}$ = 7.5 (c 1.0, H_2_O). ^1^H-NMR (400 MHz, DMSO) δ (ppm) = 7.91 (d, *J* = 7.3 Hz, 1H), 7.55–7.47 (m, 2H), 7.48–7.43 (m, 5H), 6.96 (d, *J* = 8.4 Hz, 2H), 6.66 (d, *J* = 8.4 Hz, 2H), 3.98 (d, *J* = 21.0 Hz, 2H), 3.46 (d, *J* = 21.4 Hz, 2H), 3.27-3.24 (m, 1H), 2.89 (d, *J* = 16.6 Hz, 1H), 2.73 (d, *J* = 16.6 Hz, 1H), 2.07–1.79 (m, 2H). ^13^C-NMR (100 MHz, DMSO) δ (ppm) = 167.4, 157.8, 154.0, 132.6, 130.6, 129.3, 128.8, 126.8, 114.6, 65.1, 58.2, 38.3, 28.8, 28.1. MS (ESI) *m*/*z* (%) = 436.28 (100, [M + H]^+^). 

### 3.19. (S)-2-([1,1′-Biphenyl]-4-ylsulfonamido)-3-(4-(3-aminopropoxy)phenyl)-N-hydroxypropanamide *(**19**)*

NH_2_OH^.^HCl (39 mg, 0.56 mmol) was solubilized in MeOH (220 μL) by heating to reflux until most of the salt was dissolved. The solution was cooled to <40 °C, and a solution of KOH (47 mg, 0.84 mmol) in MeOH (120 μL) was added in one portion. The resulting suspension was cooled to room temperature and was added without prior removal of precipitated material to the methyl ester **12** (39 mg, 0.07 mmol) and stirred at room temperature for 16 h. The reaction mixture was taken up in 1M HCl, extracted with EtOAc, dried over anhydrous Na_2_SO_4_, filtered, and concentrated in vacuo. The residue was purified by FCC (EtOAc) to provide the pure compound that was subsequently dissolved in 3M HCl (350 μL). The reaction solution was stirred at room temperature overnight and concentrated under reduced pressure to give the title compound (25 mg, 0.05 mmol, 71% yield). ${\left[\alpha \right]}_{D}^{22}$ = −1.8 (c 0.9, MeOH). ^1^H-NMR (400 MHz, DMSO) δ 10.69 (s, 1H), 8.86 (s, 1H), 8.08 (s, 3H), 7.74–7.34 (m, 9H), 6.96 (d, *J* = 8.4 Hz, 2H), 6.66 (d, *J* = 8.4 Hz, 2H), 3.86 (d, *J* = 6.6 Hz, 2H), 3.75 (dd, *J* = 8.7, 5.9 Hz, 1H), 2.99 (s, 1H), 2.84 (s, 2H), 2.71 (dd, *J* = 13.7, 5.5 Hz, 1H), 2.57–2.49 (m, 1H), 1.92 (s, 2H), 1.29–1.11 (m, 2H). ^13^C-NMR (50 MHz, CD_3_OD) δ 168.8, 157.4 144.8, 139.3, 139.1, 129.9, 128.8, 128.7, 128.1, 127.1, 127.0, 126.9, 114.0, 64.6, 56.5, 37.7, 37.2, 27.0. MS (ESI) *m*/*z* (%) = 467.99 (100, [M − H]^−^). 

### 3.20. (S)-Methyl 2-([1,1′-Biphenyl]-4-ylsulfonamido)-3-(4-hydroxyphenyl)propanoate *(**20**)*

In a 5 mL round bottom flask L-tyrosine methyl ester (**1**, 218 mg, 1.37 mmol) was dissolved in dry THF (1.37 mL) and dry DMF (440 μL), and a solution of biphenylsulfonyl chloride (346 mg, 1.37 mmol) in dry THF (850 μL) was added dropwise at 0 °C. The solution was warmed to room temperature and stirred for 1 h, and dry Na_2_CO_3_ (145 mg, 1.37 mmol) was then added. The consumption of biphenylsulfonyl chloride was monitored by TLC (EtOAc/PE 1:2), and the solvent was removed under reduced pressure. The solid was dissolved in 2M HCl (2 mL) and brine (2 mL), and extracted three times with EtOAc. The organic phase was washed with brine. The crude was purified by FCC (EtOAc/PE 1:2) to provide the title compound (320 mg, 0.78 mmol, 57% yield). ${\left[\alpha \right]}_{D}^{23}$ = −13.3 (c 1.0, MeOH). ^1^H-NMR (400 MHz, CD_3_OD) δ 7.70–7.62 (m, 6H), 7.46 (t, *J* = 8 Hz, 2H), 7.39 (d, *J* = 8 Hz, 1H), 6.90 (d, *J* = 8 Hz, 2H), 6.61 (d, *J* = 8 Hz, 2H), 4.00 (dd, *J* = 8, 6 Hz, 1H), 3.43 (s, 3H), 3.92–3.87 (m, 1H), 2.90 (A part of a second order ABX system, 1H), 2.75 (B part of a second order ABX system, 1H). ^13^C-NMR (50 MHz, CDCl_3_) δ 172.0, 156.0, 145.0, 139.2, 139.0, 130.0, 128.7, 128.0, 127.2, 127.0, 126.7, 114.8, 57.9, 51.1, 37.6. MS (ESI) *m*/*z* (%) = 434.18 (100, [M + Na]^+^). 

### 3.21. (S)-2-([1,1′-Biphenyl]-4-ylsulfonamido)-N-hydroxy-3-(4-hydroxyphenyl)propanamide *(**21**)*

NH_2_OH^.^HCl (428 mg, 6.2 mmol) was solubilized in MeOH (2.5 mL) by heating to reflux until most of the salt was dissolved. The solution was cooled to <40 °C, and a solution of KOH (518 mg, 9.2 mmol) in MeOH (1.3 mL) was added in one portion. The resulting suspension was cooled to room temperature and was added without prior removal of precipitated material to the methyl ester **20** (320 mg, 0.77 mmol) and stirred at room temperature for 16 h. The reaction mixture was taken up in 1M HCl, extracted with EtOAc, dried over anhydrous Na_2_SO_4_, filtered, and concentrated in vacuo. The residue was purified by FCC (DCM/MeOH 30:1) to provide, after concentration under reduced pressure, the title compound (193 mg, 0.47 mmol, 61% yield). ${\left[\alpha \right]}_{D}^{23}$ = −3.4 (c 1.0, MeOH). ^1^H-NMR (400 MHz, DMSO) δ 10.59 (s, 1H), 8.20 (s, 1H), 7.77–7.35 (m, 9H), 6.83 (d, *J* = 8.0 Hz, 2H), 6.53 (d, *J* = 8.1 Hz, 2H), 4.23 (t, *J* = 7.0 Hz, 1H), 3.68 (s, 2H). ^13^C-NMR (50 MHz, CDCl_3_) δ 168.0, 155.8, 145.5, 139.1, 137.8, 130.1, 128.9, 128.3, 127.4, 127.2 126.3, 115.4, 56.5, 37.7. MS (ESI) *m*/*z* (%) = 410.95 (100, [M − H]^−^). 

### 3.22. Enzyme Inhibition Assays 

The inhibition potency of hydroxamic acid derivatives against MMP2 and MMP9 was assayed through a fluorometric assay using the fluorogenic substrate Mca-Lys-Pro-Leu-Gly-Leu-Dpa-Ala-Arg-NH_2_ (Enzo life science). All the measurements were performed in 96-well plates with a Fluostar Optima microplate reader (BMG Labtech, Ortenberg, Germany). Excitation and emission wavelengths were 320 and 420 nm, respectively. All incubations were performed at 28 °C in 50 mM TrisHCl, 150 mM NaCl, 10 mM CaCl_2_, 0.05% Brij 35, 1% DMSO at pH 7.5. The inhibitors were pre-incubated with enzymes (1 nM) for 5 min at room temperature before the reaction was started by the addition of the fluorogenic substrate (3 µM). The decrease of fluorescence was monitored over 30 min (λ_ex_ = 320 nm, λ_em_ = 420 nm) at 28 °C. The percentages of inhibition for the test compounds were determined through the equation (1 − V_s_/V_o_) × 100, where vs. is the initial velocity in the presence of the inhibitor and V_0_ is the initial velocity of the uninhibited reaction. The IC_50_ values were obtained by dose-response measurements using inhibitor range of concentrations 0.00001–20 μM and enzyme concentration equal to 1 nM. A detergent-based assay was used to determine the presence of promiscuous inhibitors. All the experiments were performed in triplicates and data collected were analyzed using Graphpad 5.0 Software Package (GraphPad Prism Inc., San Diego, CA, USA).

### 3.23. Solid-Phase Integrin Binding Assay

Purified α_v_β_3_ receptors (Sino Biological Europe GmbH, Eschborn, Germany) were diluted to 0.5 μg/mL in coating buffer containing Tris-HCl (20 mmol/L; pH 7.4), NaCl (150 mmol/L), MnCl_2_ (1 mmol/L), CaCl_2_ (2 mmol/L) and MgCl_2_ (1 mmol/L). An aliquot of diluted receptors (100 μL per well) was added to 96-well microtiter plates (MW 96F Medisorp Straight Nunc, Thermo Fischer Scientific, Waltham, MA, USA) and incubated, overnight, at 4 °C. The plates were then incubated with blocking solution (coating buffer plus 5% bovine serum albumin) for an additional 2 h at room temperature to block nonspecific binding followed by 3 h incubation at room temperature with various concentrations of test compounds in the presence of vitronectine (1 μg/mL, Sigma Aldrich, Merck KGaA, Darmstadt, Germany) biotinylated by using EZ-Link Sulfo-NHS-Biotinylation kit (Thermo Fisher, Waltham, MA, USA). After washing, the plates were incubated for 1 h at room temperature with streptavidin–biotinylated peroxidase complex (GE Healthcare, Chicago, IL, USA), followed by 30 min incubation with substrate reagent solution (100 μL; R&D Systems) before stopping the reaction by addition of H_2_SO_4_ (2 N, 50 μL). Absorbance at 415 nm was read with a BMG Labtech Fluostar Optima microplate reader. All the experiments were performed in triplicates and data collected were analyzed using the GraphPad 5.0 Software Package.

### 3.24. Inhibition of Cell Adhesion Assay 

The expression levels of α_V_β_3_ integrin receptors on M21 melanoma cell lines were reported previously [26]. Next, 96 wells plates were coated overnight, at 4 °C, with vitronectin (5 μg/mL in PBS) (V8379 Sigma). Plates were, then washed with phosphate buffered saline (PBS) solution and incubated at 37 °C for 1 h with PBS containing 1% bovine serum albumin (BSA). M21 cells were centrifugated (RT, at 700× *g*) in PBS, to remove serum. Cells were counted and suspended in serum-free medium at 5.0 × 10^5^ cells/mL. Melanoma cell suspensions were pre-incubated with different amounts of the compounds. The final concentration ranged from 30 uM to 0.3 uM, using cilengitide as a control. The incubation was performed at 37 °C for 30 min to allow the ligand-receptor equilibrium to be reached. Next, cells were plated on VN substrata (5–6 × 10^4^ cells/well) and incubated at 37 °C for 1 h. The assays were conducted in the presence of 2 mmol/L MnCl_2_ [26]. At the end of the incubation, plates were washed with PBS to remove the non-adherent cells, and 200 μL of 0.5% crystal violet solution in 20% methanol were added. After 2 h of static incubation at 4 °C, plates were examined at 540 nm in a counter ELX800 (Bio TEK Instruments, Winooski, VT, USA). Experiments were done in triplicate and repeated at least three times. The values are expressed as % inhibition of cell adhesion relative to cells exposed to vehicle alone (PBS).

### 3.25. Molecular Modeling

Automated docking studies were carried out using the Lamarckian Genetic Algorithm (LGA) as a search engine implemented in the Autodock 4.0.1 program [28]. The AutoDockTools 1.4.5 (ADT) graphical interface [29] was used to prepare integrin and ligands PDBQT files. Coordinates of compound 19 were generated using Spartan (version 5.147, Wavefunction, Inc. Irvine, CA, USA) using Monte Carlo method within MMFF94 force field, and then energy-minimized through the AM1 semi-empirical method [30] to calculate the equilibrium geometry. The coordinates of α_v_β_3_ receptor and MMP2 were retrieved from the Protein Data Bank (PDB code: 1L5G and 1QIB for α_v_β_3_ and MMP2, respectively), and ligand–protein complex was unmerged for achieving free protein structure. Water molecules were removed. For protein structures and compound 19, all hydrogen atoms were added, Gasteiger charges were computed, and nonpolar hydrogen atoms were merged. A charge value of +2.0 was successively added to each Mn atoms of α_v_β_3_ receptor and to Zn atom of MMP2. Three-dimensional energy scoring grids of 0.375 Å resolution and 40 × 40 × 40 Å dimensions were computed. The center of the grid was set to be coincident with mass center of ligands preliminary fitted on the X-ray structure of c[RGDf(Me)V] in the α_v_β_3_ complex (1L5G) and on Zn atom for MMP2 enzyme (1QIB). A total of 50 runs with a maximum of 2,500,000 energy evaluations were carried out for each ligand, using the default parameters for LGA. Low energy ligand-protein complexes were subjected to AMMP energy minimization using VegaZZ [31], then cluster analysis was performed on docked results using a root-mean-square (rms) tolerance of 1.5 Å. Analysis of the binding mode, calculation of the binding energy, and prediction of the binding activity of docked conformations were carried out using Autodock plugin within the PyMol software v0.99 [32].

## 4. Conclusions

Driven by the current interest in multitarget approaches in cancer treatment, in the context of angiogenesis, we reasoned that by combining the inhibition of α_v_β_3_ and MMP2 we could deliver a synergistic blockade of tumor cell migration, invasion and metastasis. Accordingly, we hypothesized the possibility of identifying a common pharmacophore in the binding cavity of MMP2 and α_v_β_3_ to be studied with tailored tyrosine-derived peptidomimetics bearing the necessary functional groups to bind to key pharmacophoric elements of MMP2 and α_v_β_3_ RGD integrin. The synthetic strategy allowed to furnish a pool of peptidomimetics varying in the three key functional groups, and subsequent bioassays towards purified proteins and on a melanoma cell line allowed to identify a hydroxamic acid derivative capable of displaying dual activity towards MMP2 and α_v_β_3_ RGD integrin. Cell based assays corroborated the bioactivity profile of the compound, and molecular docking calculations allowed to ascertain the binding mode within the two protein cavities, confirming the hypothesized common binding region for the two proteins. 

## Figures and Tables

**Table 1:** Binding activity data of peptidomimetics determined using a fluorogenic peptide substrate ^a^.

Cmpd	Structure	MMP2 Inhibition % (10; 50 μM)	MMP9 Inhibition % (10; 50 μM)	IC_50_ (MMP2)	IC_50_ (MMP9)
9	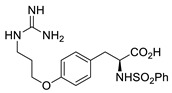	0; 21	0; 0	-	-
10	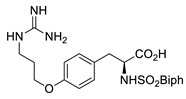	16; 41	0; 0	-	-
16	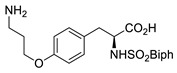	60; 79	0; 0	1.2 μM	-
17	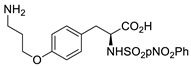	0; 0	0; 0	-	-
18	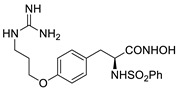	52; 75	42; 63	0.76 μM	1.3 μM
19	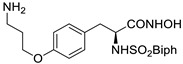	100; 100	100; 100	55 nM	38 nM
21	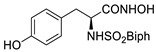	97; 100	63; 73	37 nM	361 nM

^a^ Mean from three different assays, errors were in the range of 5–10% of the reported values; IC_50_ values were calculated as the concentration of compound required for 50% inhibition, as estimated by the GraphPad Prism software.

**Table 2:** Inhibition of biotinylated vitronectin specific binding to purified human integrin proteins α_v_β_3_^a^.

Entry	Structure	Inhibition % at 10 μM	Inhibition % at 1 μM	Inhibition % at 0.1 μM
**9**	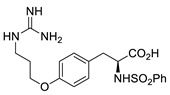	54	18	0
**10**	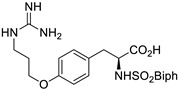	5	1	8
**16**	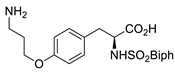	19	26	26
**17**	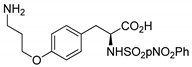	0	0	0
**18**	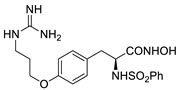	27	0	0
**19**	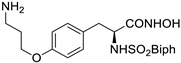	88	41	21
**21**	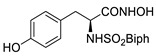	2	12	20

^a^ Mean from three different assays, errors were in the range of 5–10% of the reported values.

**Table 3:** Percentage of inhibition of integrin-mediated M21 cell adhesion to vitronectin ^a^.

Cmpd	Structure	Inhibition % at 30 μM	Inhibition % at 3 μM	Inhibition % at 0.3 μM
**Cilengitide**	**c[RGDf(Me)V]**	62	76	80
**9**	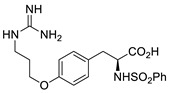	75.9	62.5	41.4
**10**	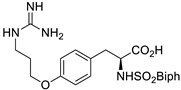	76.9	76.7	43.8
**16**	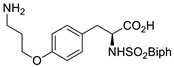	68.5	28.9	28.0
**18**	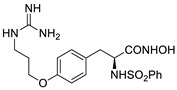	74.1	64.3	44.9
**19**	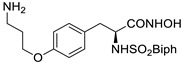	65.7	35.8	57.8
**21**	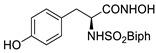	17.4	28.8	29.7

^a^ Experiments were done in triplicate and repeated at least three times. The values are expressed as % inhibition of cell adhesion relative to cells exposed to vehicle alone (PBS).

## Supplementary Materials

The following are available online, Copies of ^1^H- and ^13^C-NMR spectra of compounds **3**–**21**.
