# Supplementary material for: Identification of a Common Pharmacophore for Binding to MMP2 and RGD Integrin: Towards a Multitarget Approach to Inhibit Cancer Angiogenesis and Metastasis
Source: Molecules. 2022 Feb 12;27(4):1249. doi: 10.3390/molecules27041249 (PMC8879803; doi:10.3390/molecules27041249)
Supplement: Supplementary file 1 [file molecules-27-01249-s001.zip › molecules-1564280-supplementary.pdf]

molecules

Supplementary Materials

Identification of a common pharmacophore for binding to MMP2 and RGD integrin: towards a multitarget approach to inhibit cancer angiogenesis and metastasis

Lorenzo Baldini [1], Elena Lenci [1], Francesca Bianchini [2] and Andrea Trabocchi [1,*]

[1] Department of Chemistry 'Ugo Schiff', University of Florence, I-50019 Sesto Fiorentino, Italy
[2] Department of Biomedical, Experimental and Clinical Sciences ''Mario Serio'', University of Florence, I-50134 Florence, Italy
* Correspondence: andrea.trabocchi@unifi.it

Copies of ^{1}H and ^{13}C-NMR spectra of compounds 3–21 **S2-S20**

Molecules **2022**, *27*, 1249. https://doi.org/10.3390/molecules27041249 www.mdpi.com/journal/molecules

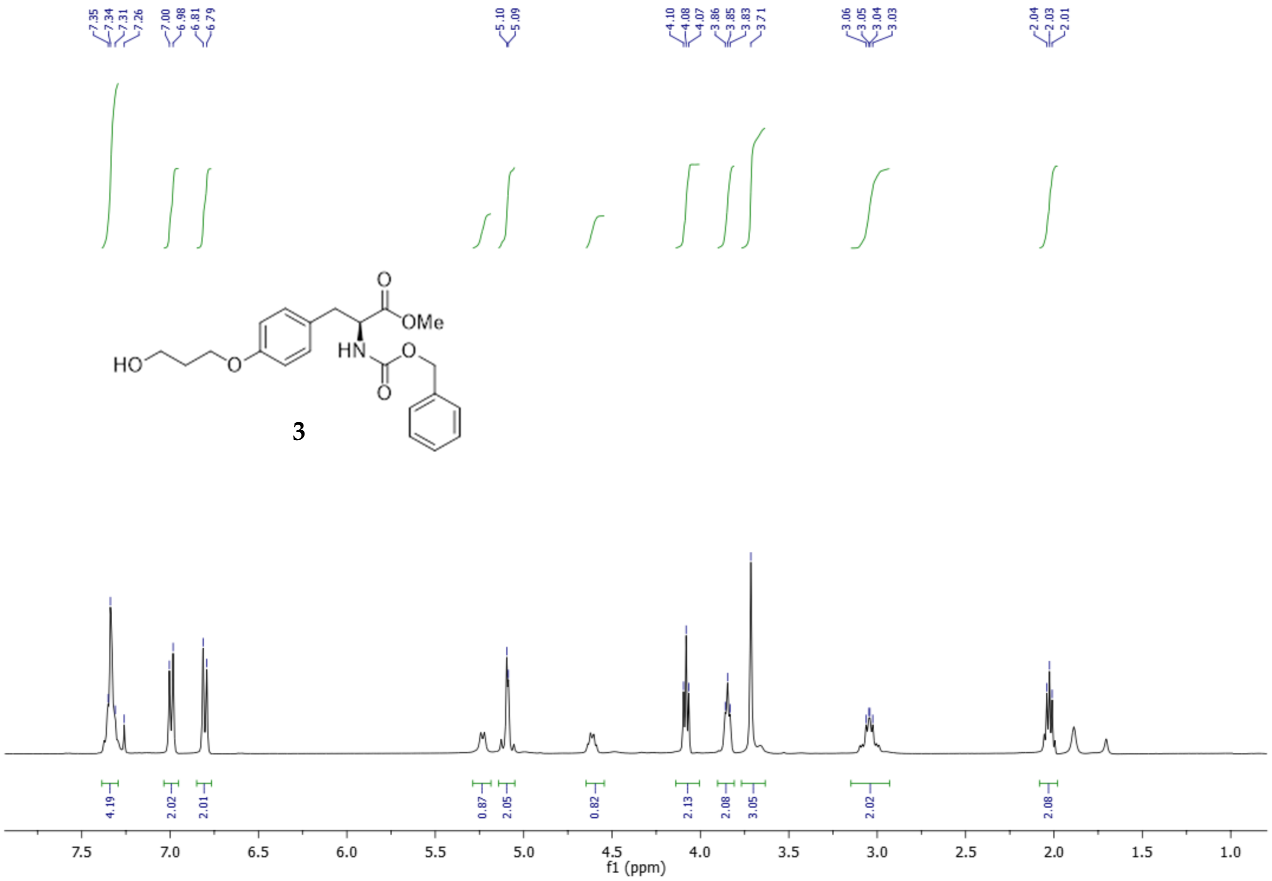
7.35
7.34
7.31
7.26
7.00
6.98
6.81
6.79
5.10
5.09
4.10
4.08
4.07
3.86
3.85
3.83
3.71
3.06
3.05
3.04
3.03
2.04
2.03
2.01
HO
O
HN
O
OMe
O
O
3
4.19
2.02
2.01
0.87
2.05
0.82
2.13
2.08
3.05
2.02
2.08
f1 (ppm)

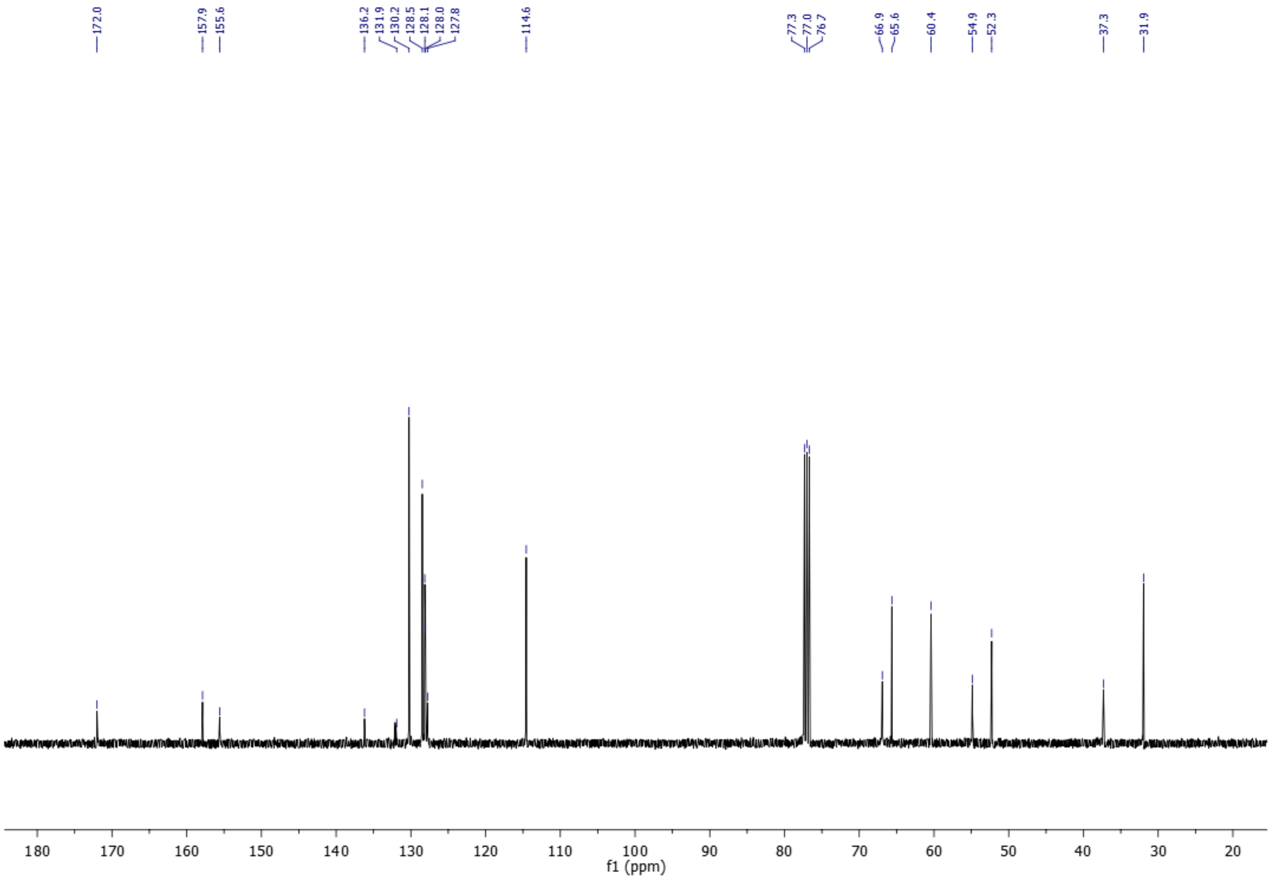
172.0
157.9
155.6
136.2
131.9
130.2
128.5
128.1
128.0
127.8
114.6
77.3
77.0
76.7
66.9
65.6
60.4
54.9
52.3
37.3
31.9
f1 (ppm)

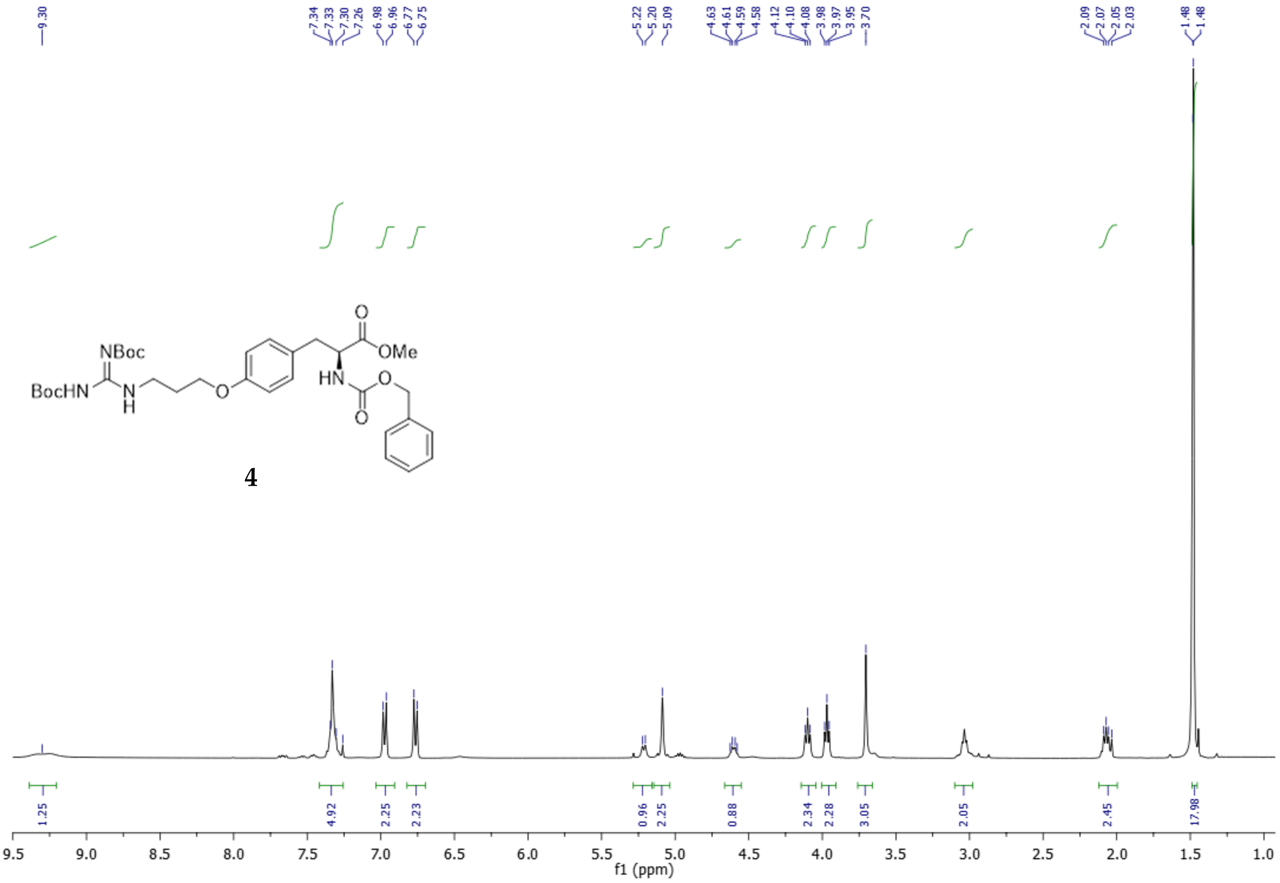
9.30
7.34 7.33 7.30 7.26 6.98 6.96 6.77 6.75
5.22 5.20 5.09
4.63 4.61 4.59 4.58 4.12 4.10 4.08 3.98 3.97 3.95 3.70
2.09 2.07 2.05 2.03
1.48 1.48
NBoc
BocHN
N
H
O
OMe
HN
O
O
4
1.25
4.92
2.25
2.23
0.96
2.25
0.88
2.34
2.28
3.05
2.05
2.45
17.98
9.5 9.0 8.5 8.0 7.5 7.0 6.5 6.0 5.5 5.0 4.5 4.0 3.5 3.0 2.5 2.0 1.5 1.0
f1 (ppm)

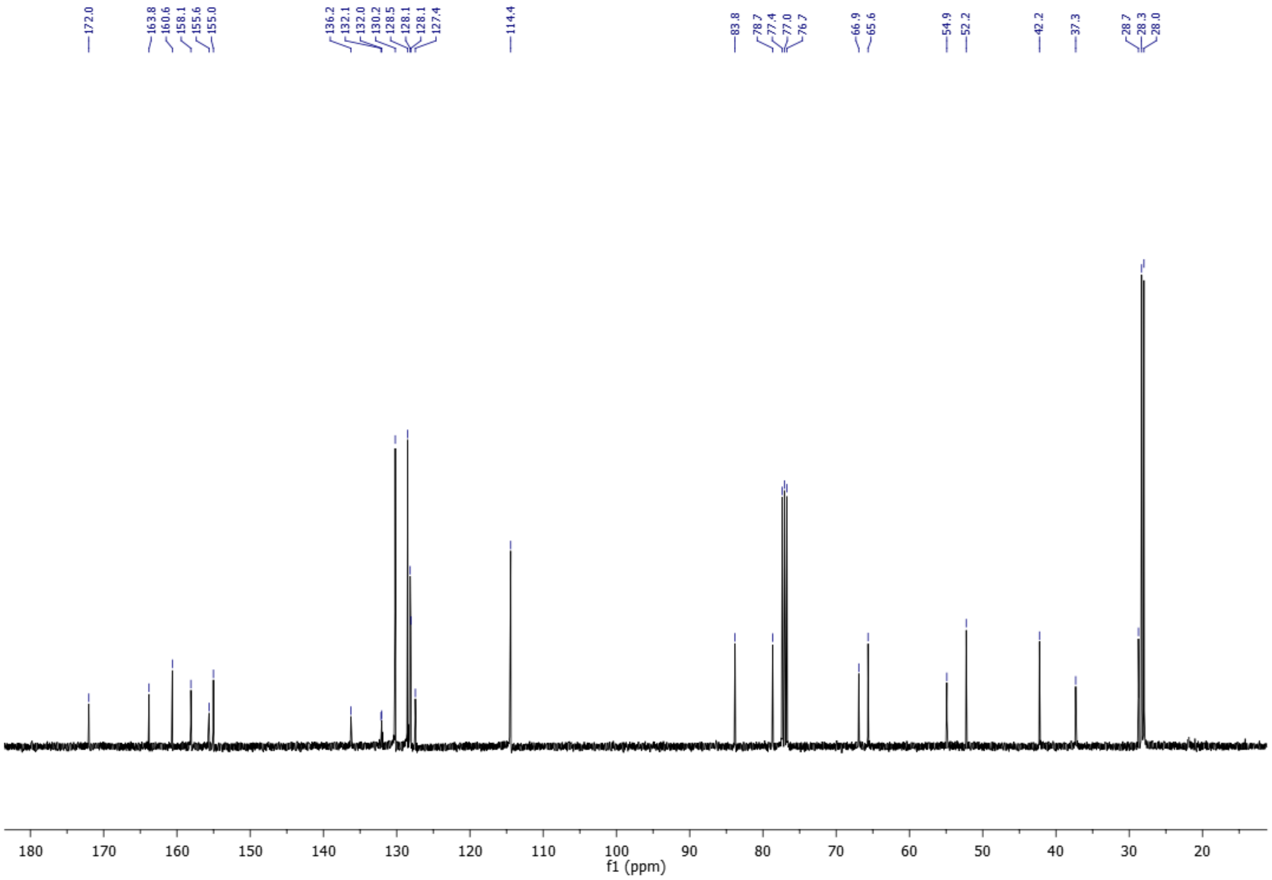
172.0
163.8 160.6 158.1 155.6 155.0
136.2 132.1 132.0 130.2 128.5 128.1 128.1 127.4
114.4
83.8 78.7 77.4 77.0 76.7
66.9 65.6
54.9 52.2
42.2
37.3
28.7 28.3 28.0
180 170 160 150 140 130 120 110 100 90 80 70 60 50 40 30 20
f1 (ppm)

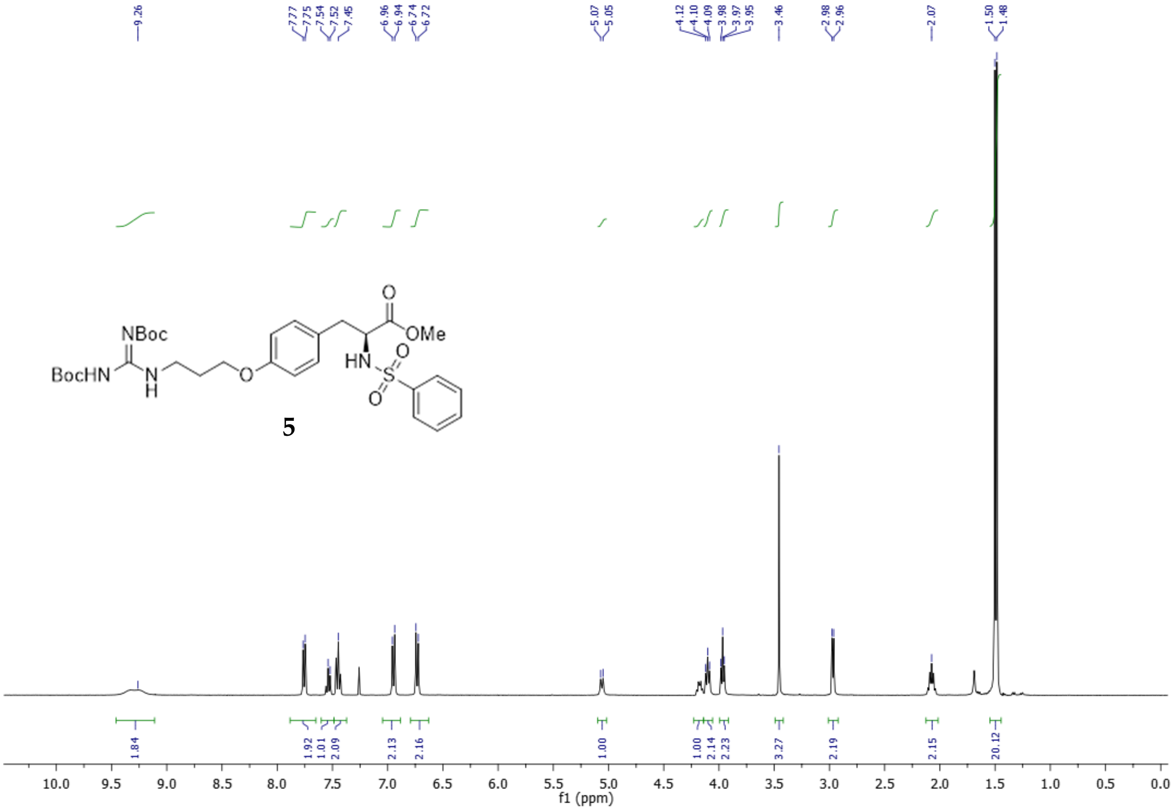
NBoc
BocHN
OMe
HN
5
f1 (ppm)

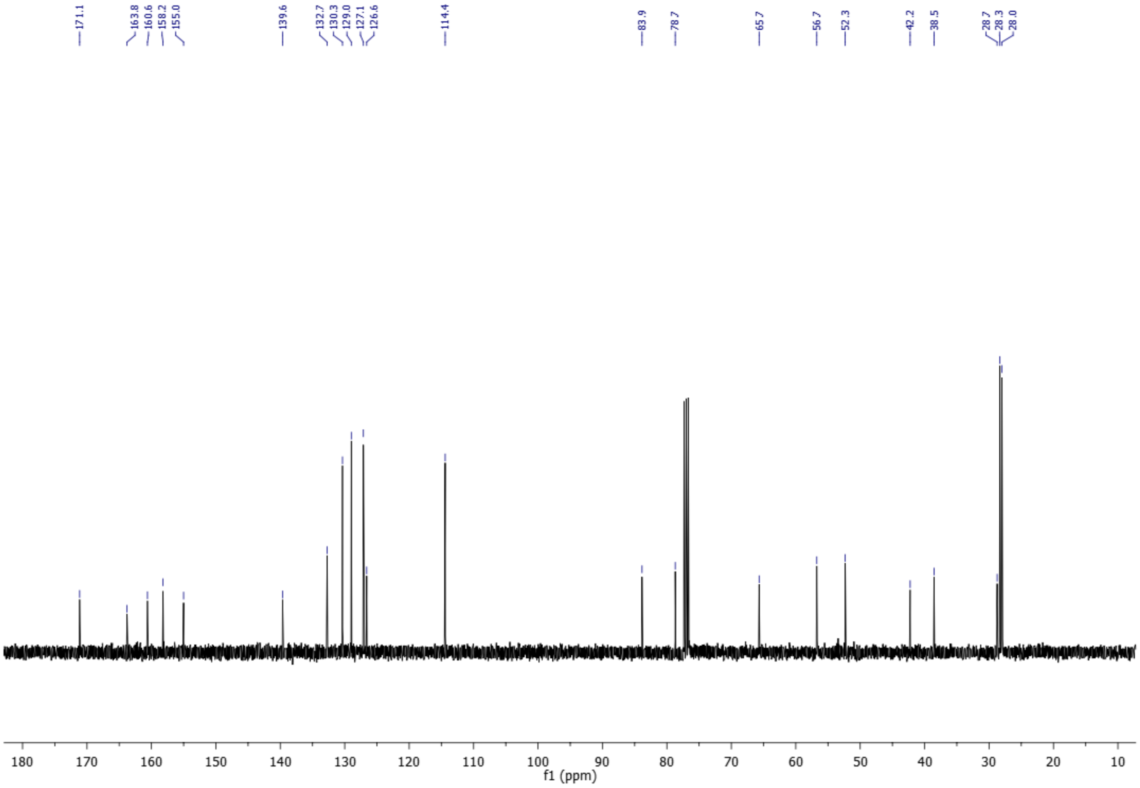
f1 (ppm)

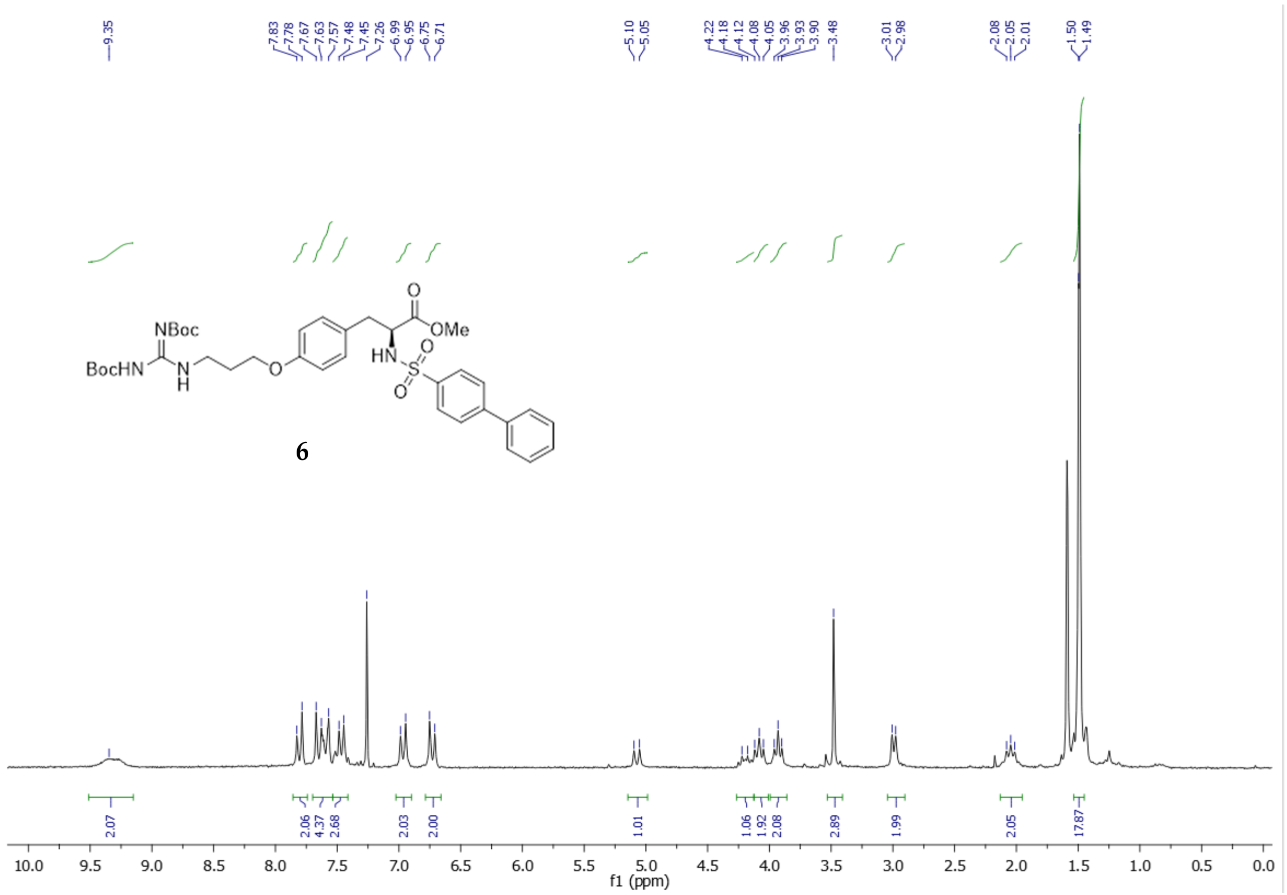
NBoc
BocHN
N
H
O
O
OMe
HN
S
O
O
6
f1 (ppm)

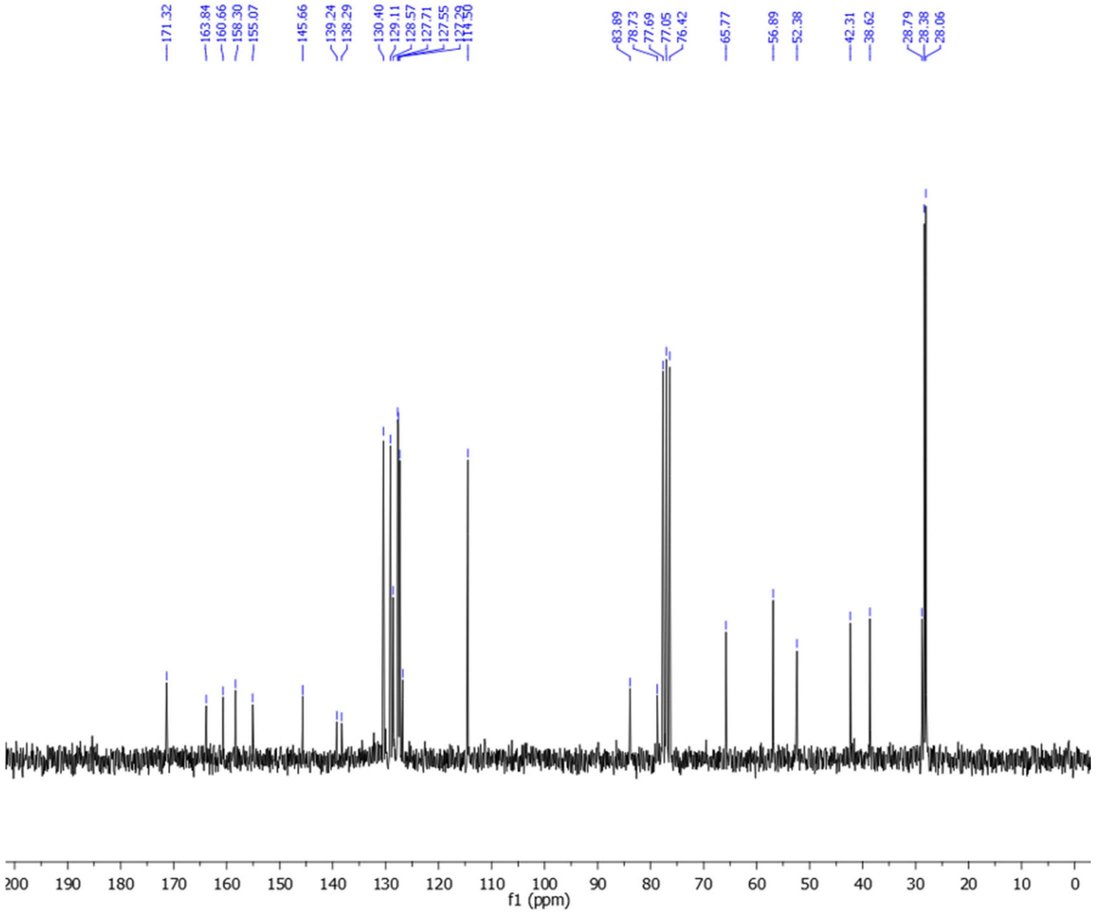
f1 (ppm)

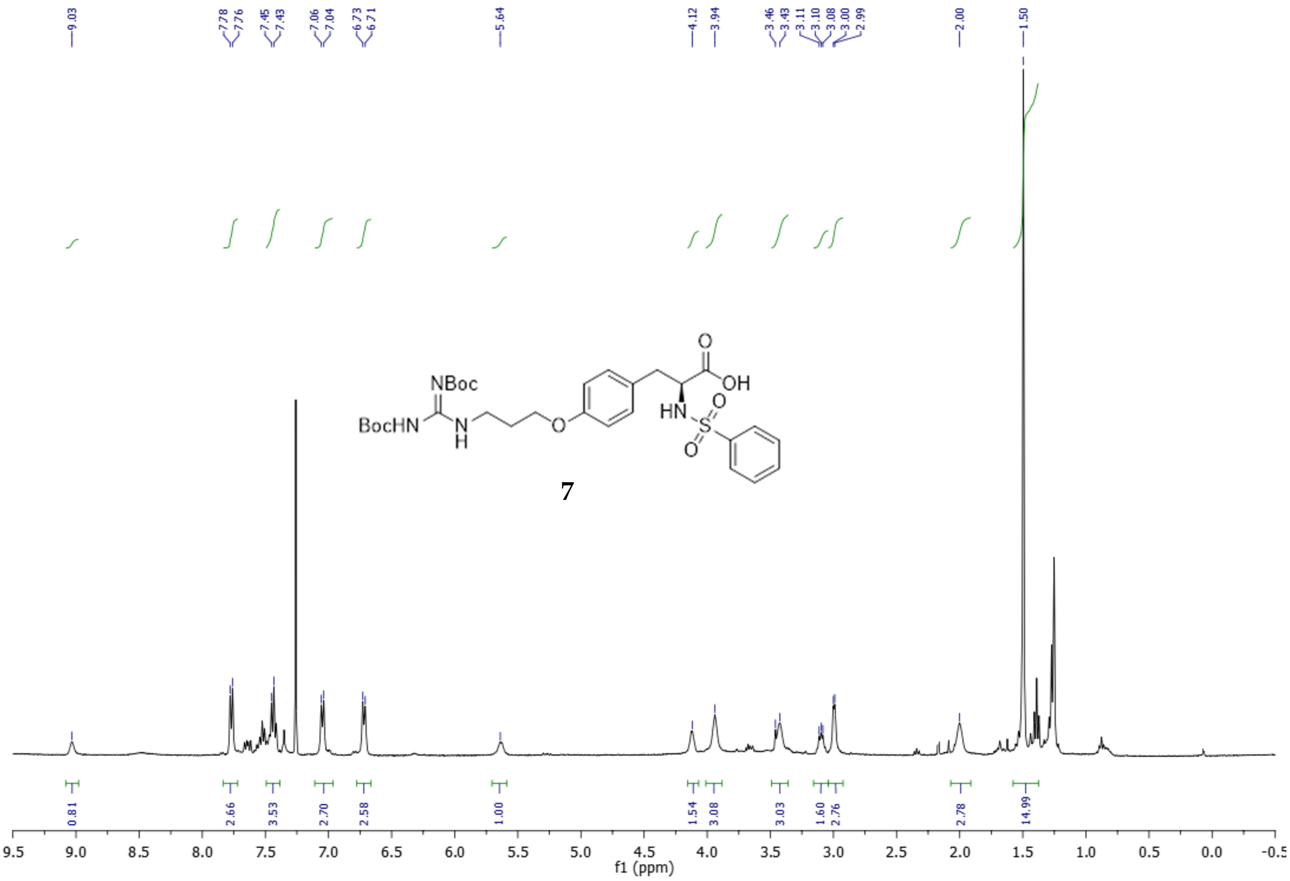
9.03
7.78
7.76
7.45
7.43
7.06
7.04
6.73
6.71
5.64
4.12
3.94
3.46
3.43
3.11
3.10
3.08
3.00
2.99
2.00
1.50
NBoc
BocHN
N
H
O
O
OH
HN
S
O
O
7
0.81
2.66
3.53
2.70
2.58
1.00
1.54
3.08
3.03
1.60
2.76
2.78
14.99
f1 (ppm)

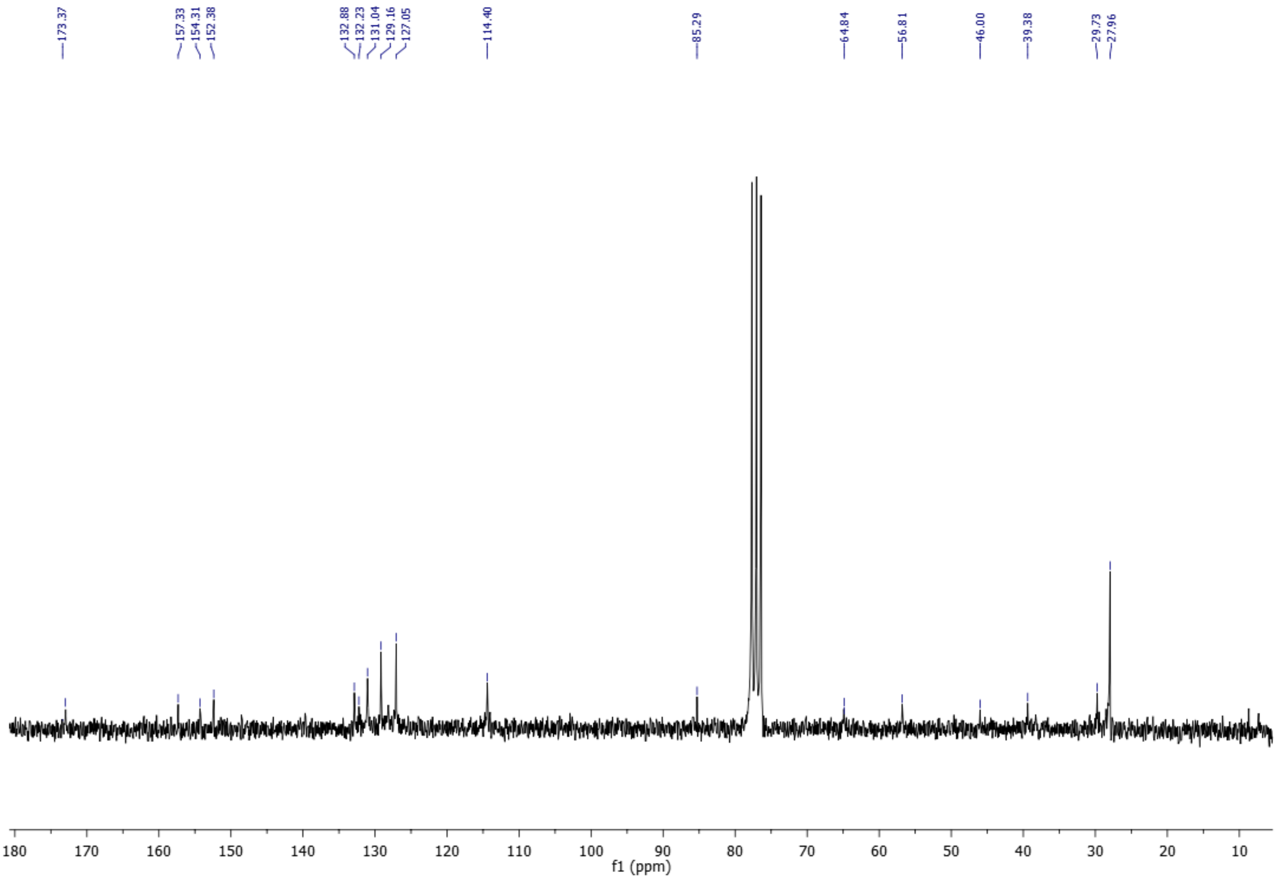
173.37
157.33
154.31
152.38
132.88
132.23
131.04
129.16
127.05
114.40
85.29
64.84
56.81
46.00
39.38
29.73
27.96
f1 (ppm)

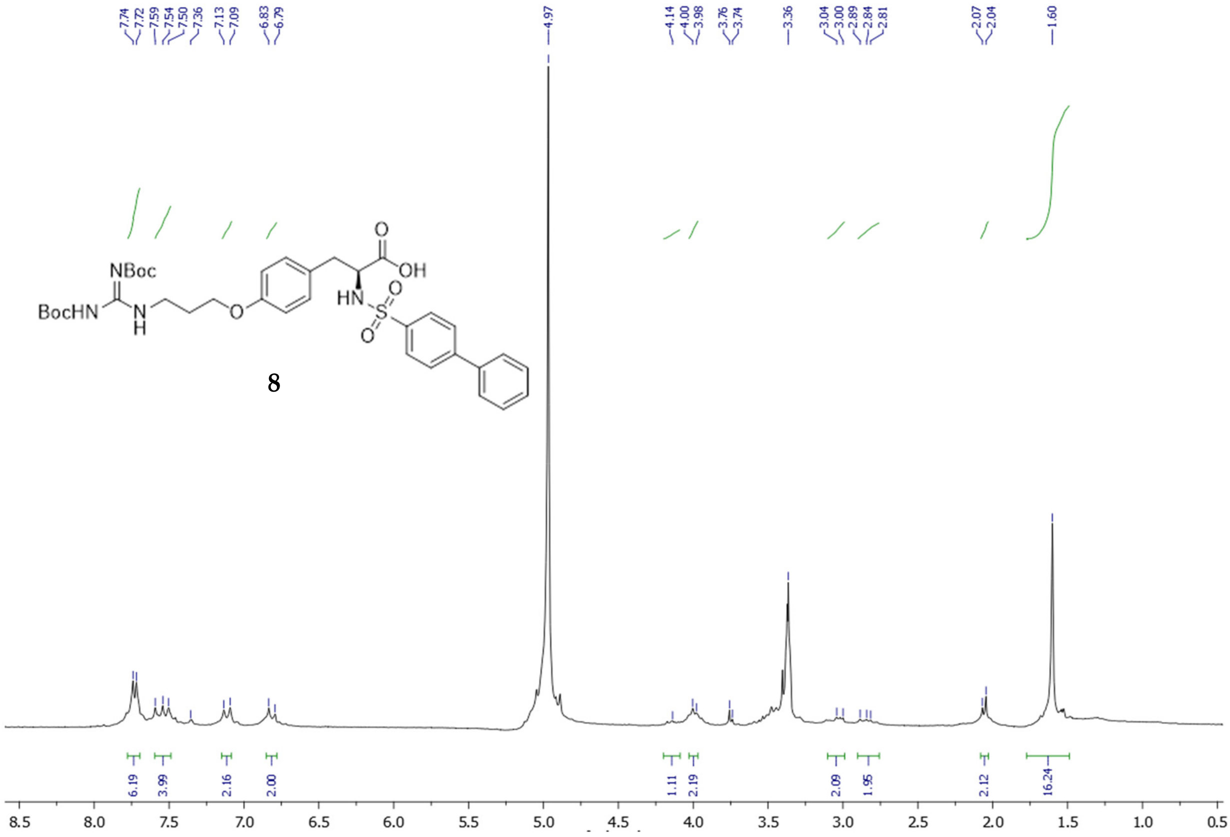
7.74
7.72
7.59
7.54
7.50
7.36
7.13
7.09
6.83
6.79
4.97
4.14
4.00
3.98
3.76
3.74
3.36
3.04
3.00
2.89
2.84
2.81
2.07
2.04
1.60
NBoc
BocHN
N
H
O
O
OH
HN
S
O
O
8
6.19
3.99
2.16
2.00
1.11
2.19
2.09
1.95
2.12
16.24
8.5 8.0 7.5 7.0 6.5 6.0 5.5 5.0 4.5 4.0 3.5 3.0 2.5 2.0 1.5 1.0 0.5

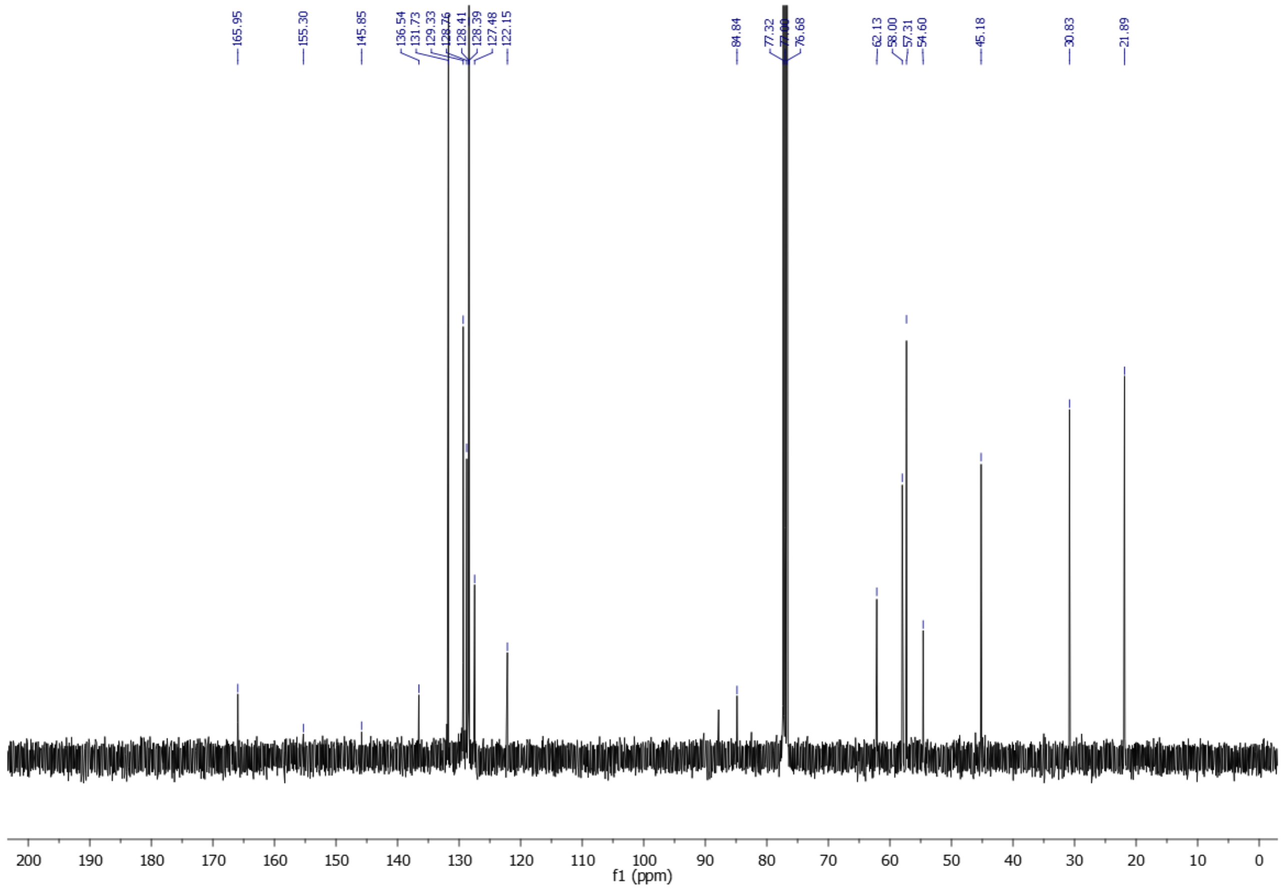
165.95
155.30
145.85
136.54
131.73
129.33
128.76
128.41
128.39
127.48
122.15
84.84
77.32
77.00
76.68
62.13
58.00
57.31
54.60
45.18
30.83
21.89
200 190 180 170 160 150 140 130 120 110 100 90 80 70 60 50 40 30 20 10 0
f1 (ppm)

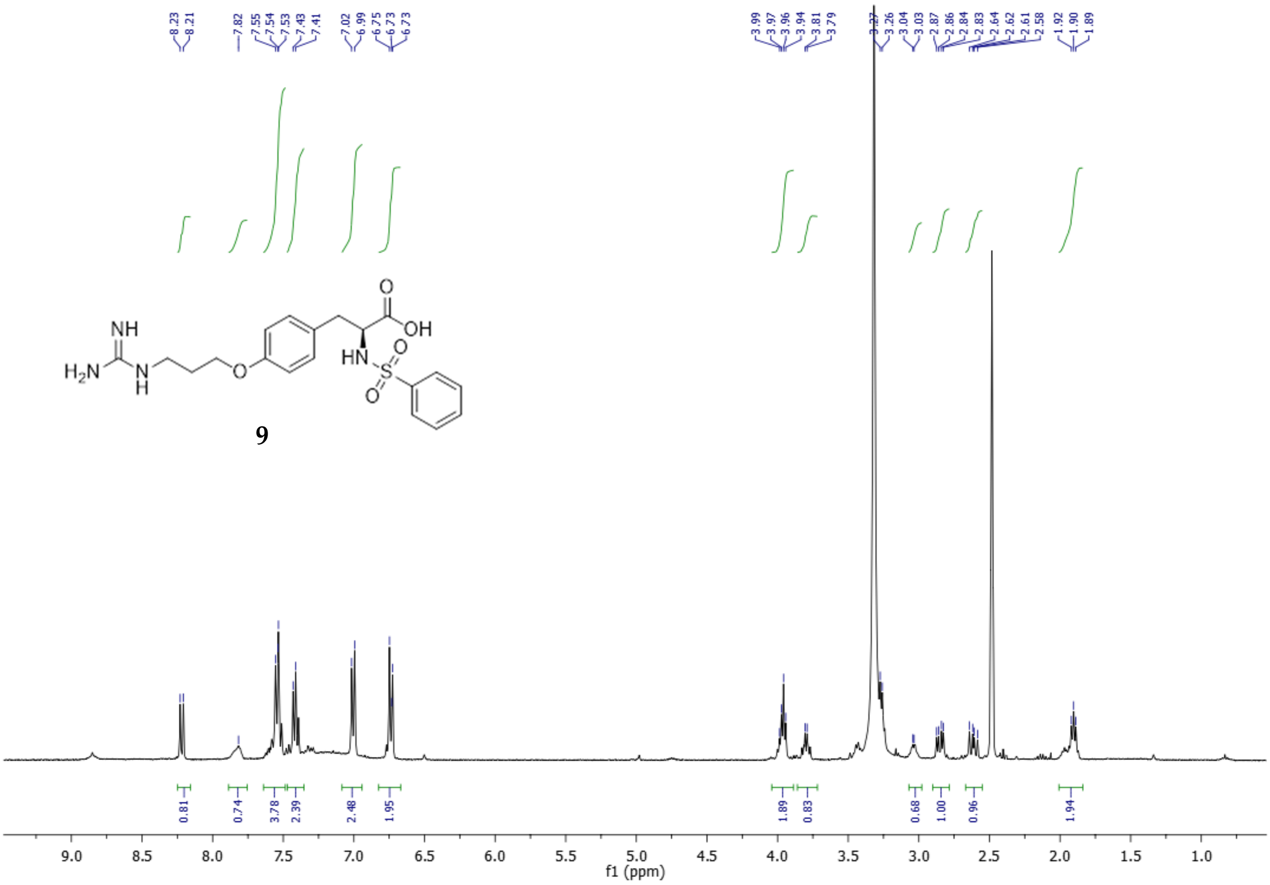
8.23
8.21
7.82
7.55
7.54
7.53
7.43
7.41
7.02
6.99
6.75
6.73
6.73
3.99
3.97
3.96
3.94
3.81
3.79
3.27
3.26
3.04
3.03
2.87
2.86
2.84
2.83
2.64
2.62
2.61
2.58
1.92
1.90
1.89
NH
H_2N
N
H
O
O
OH
HN
S
O
O
9
0.81
0.74
3.78
2.39
2.48
1.95
1.89
0.83
0.68
1.00
0.96
1.94
9.0
8.5
8.0
7.5
7.0
6.5
6.0
5.5
5.0
4.5
4.0
3.5
3.0
2.5
2.0
1.5
1.0
f1 (ppm)

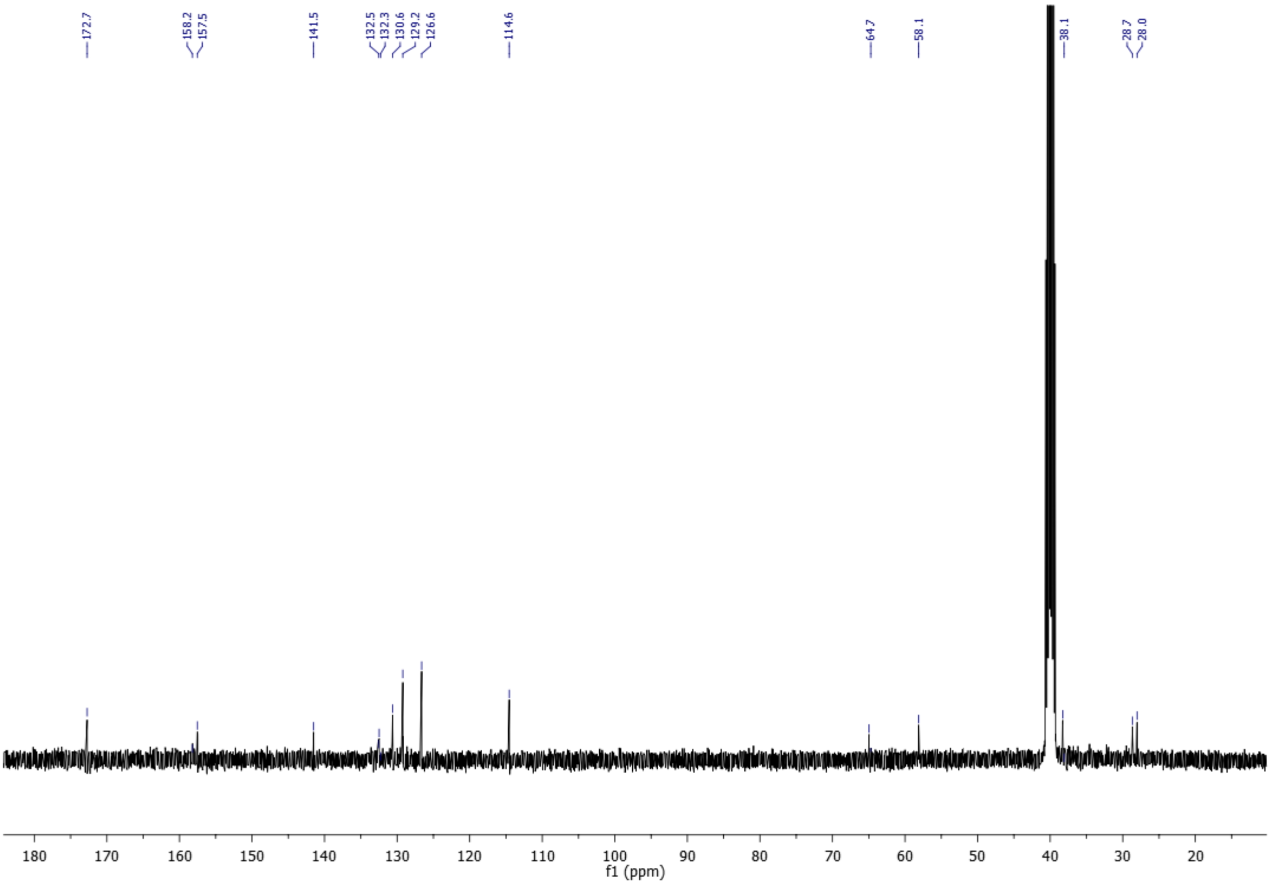
172.7
158.2
157.5
141.5
132.5
132.3
130.6
129.2
126.6
114.6
64.7
58.1
38.1
28.7
28.0
180
170
160
150
140
130
120
110
100
90
80
70
60
50
40
30
20
f1 (ppm)

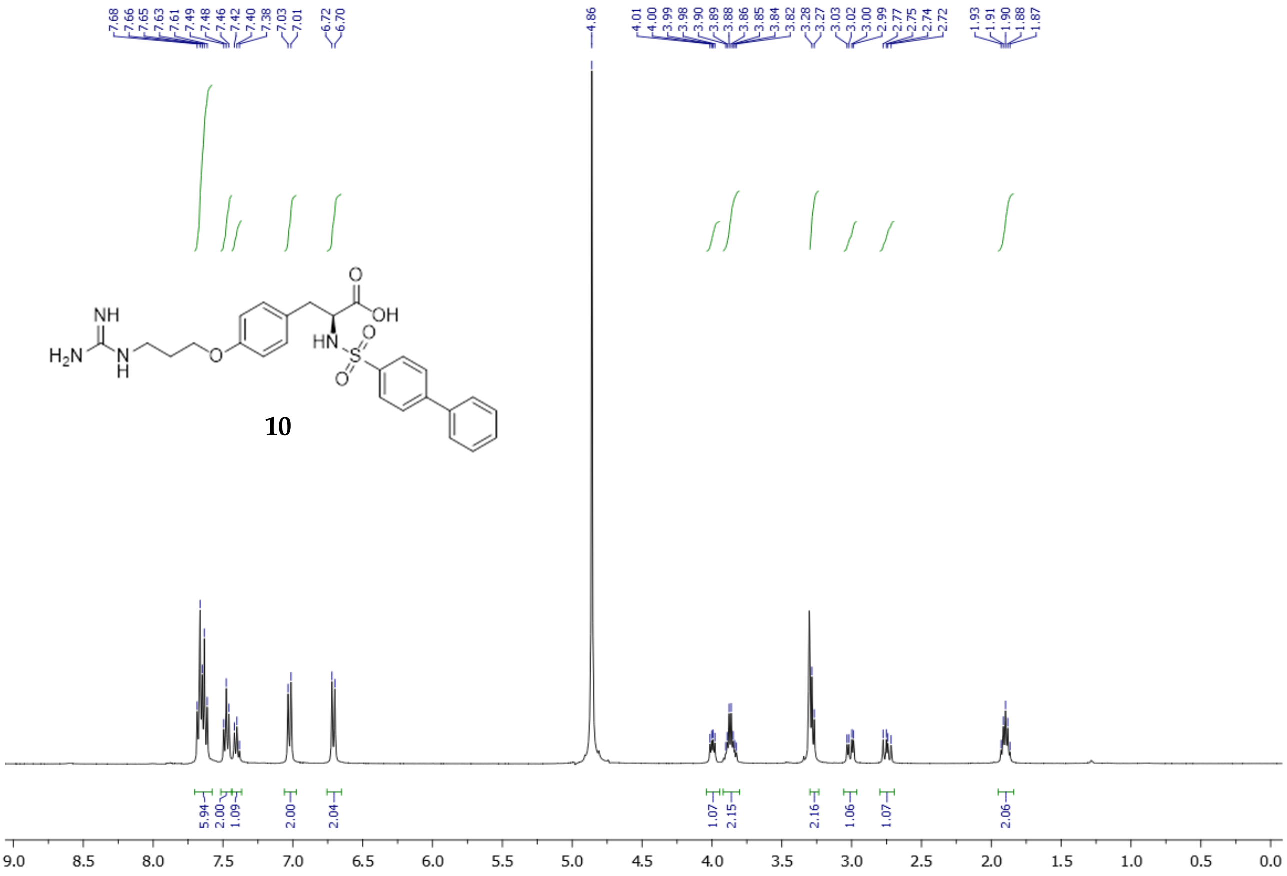
10

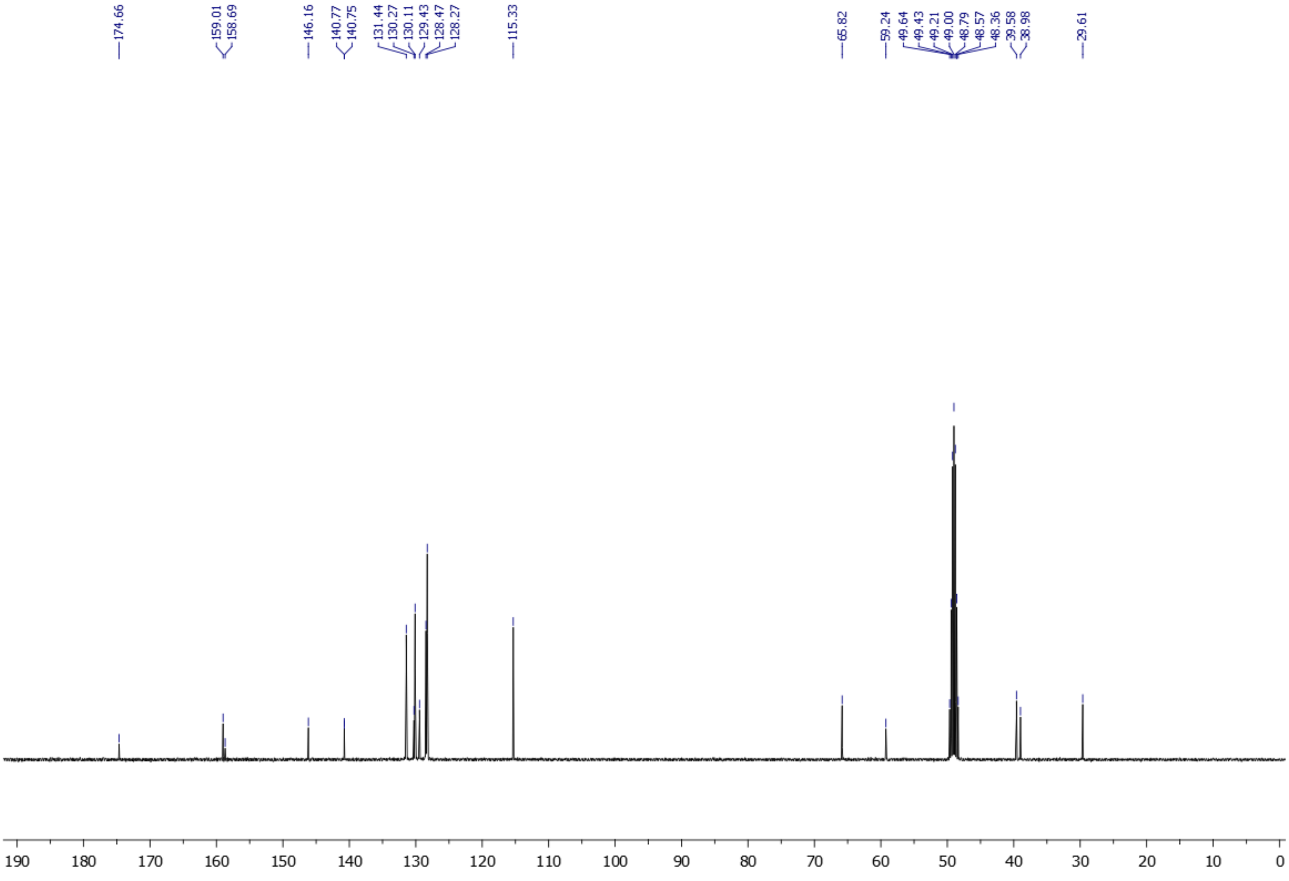

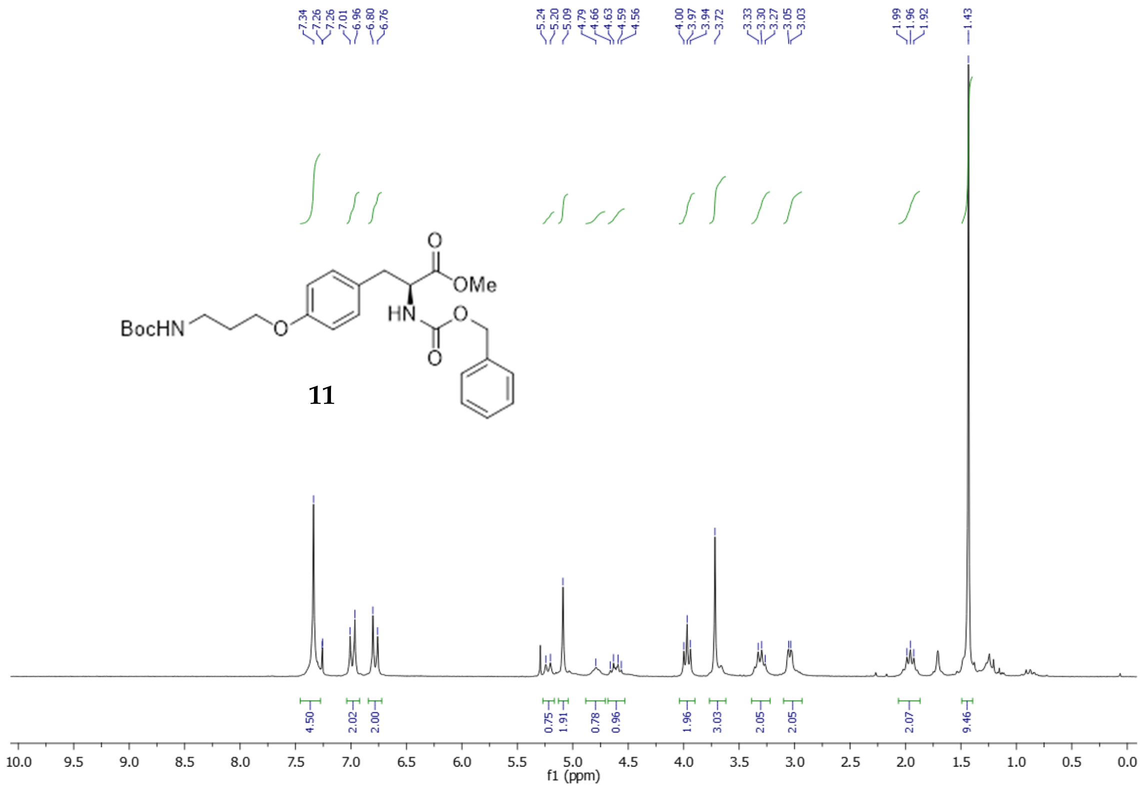
7.34
7.26
7.26
7.01
6.96
6.80
6.76
5.24
5.20
5.09
4.79
4.66
4.63
4.59
4.56
4.00
3.97
3.94
3.72
3.33
3.30
3.27
3.05
3.03
1.99
1.96
1.92
1.43
O
OMe
BocHN
O
HN
O
O
11
4.50
2.02
2.00
0.75
1.91
0.78
0.96
1.96
3.03
2.05
2.05
2.07
9.46
10.0 9.5 9.0 8.5 8.0 7.5 7.0 6.5 6.0 5.5 5.0 4.5 4.0 3.5 3.0 2.5 2.0 1.5 1.0 0.5 0.0
f1 (ppm)

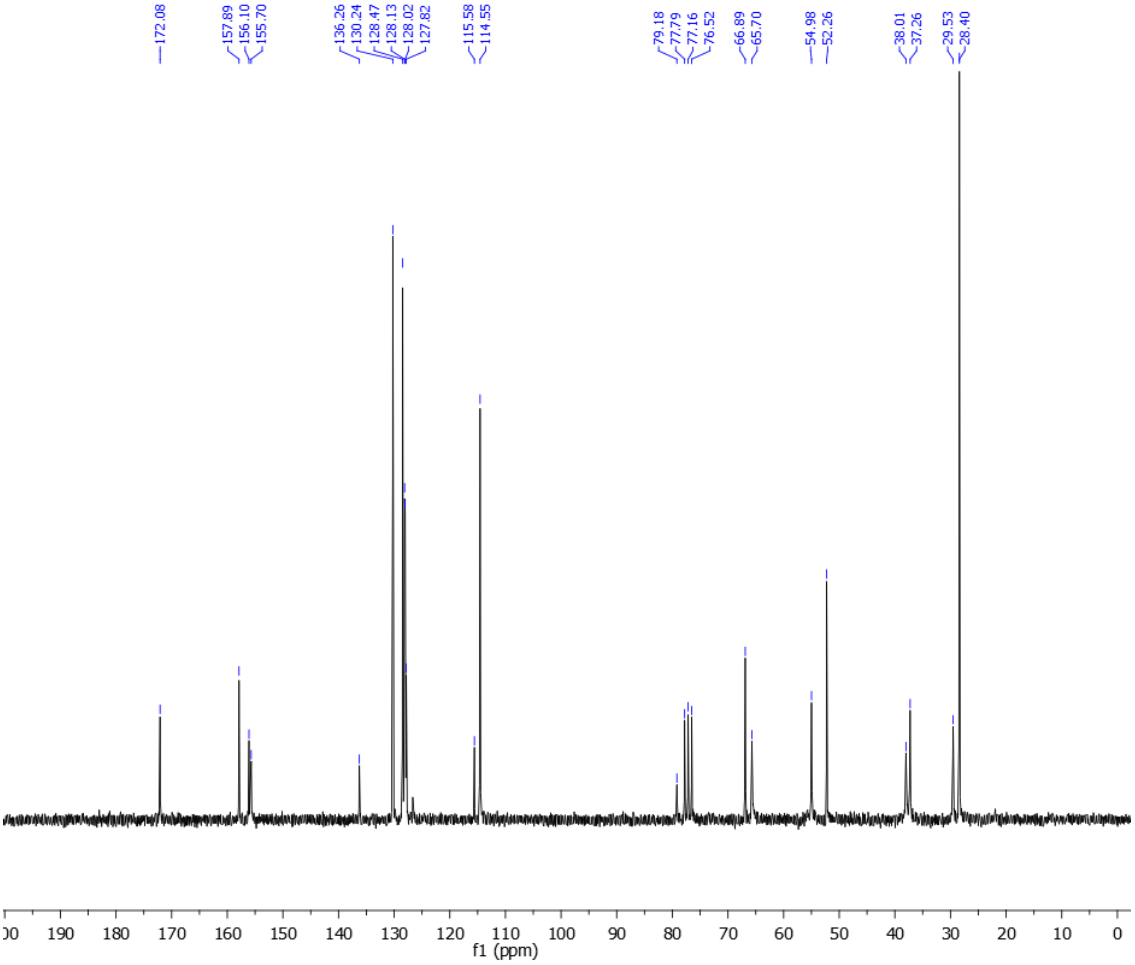
172.08
157.89
156.10
155.70
136.26
130.24
128.47
128.13
128.02
127.82
115.58
114.55
79.18
77.79
77.16
76.52
66.89
65.70
54.98
52.26
38.01
37.26
29.53
28.40
190 180 170 160 150 140 130 120 110 100 90 80 70 60 50 40 30 20 10 0
f1 (ppm)

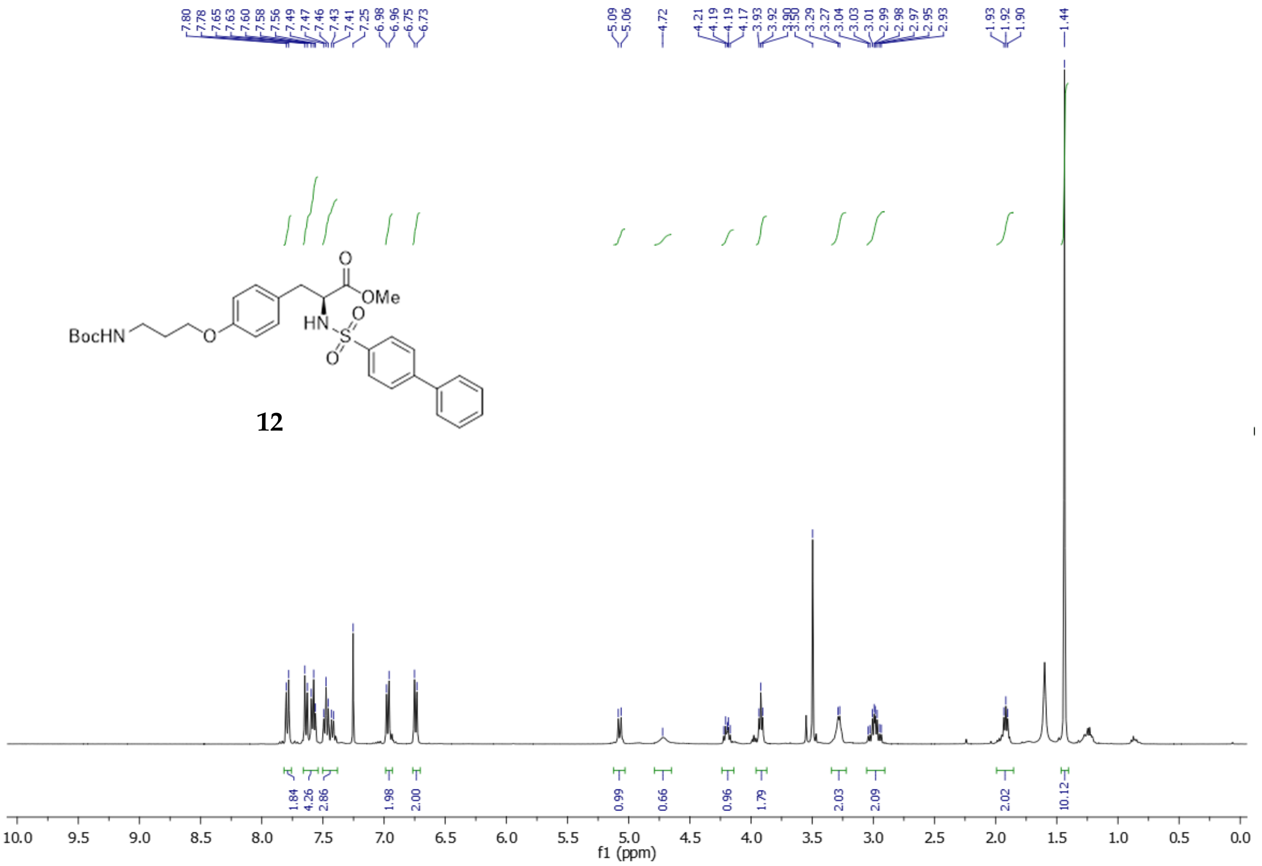

12

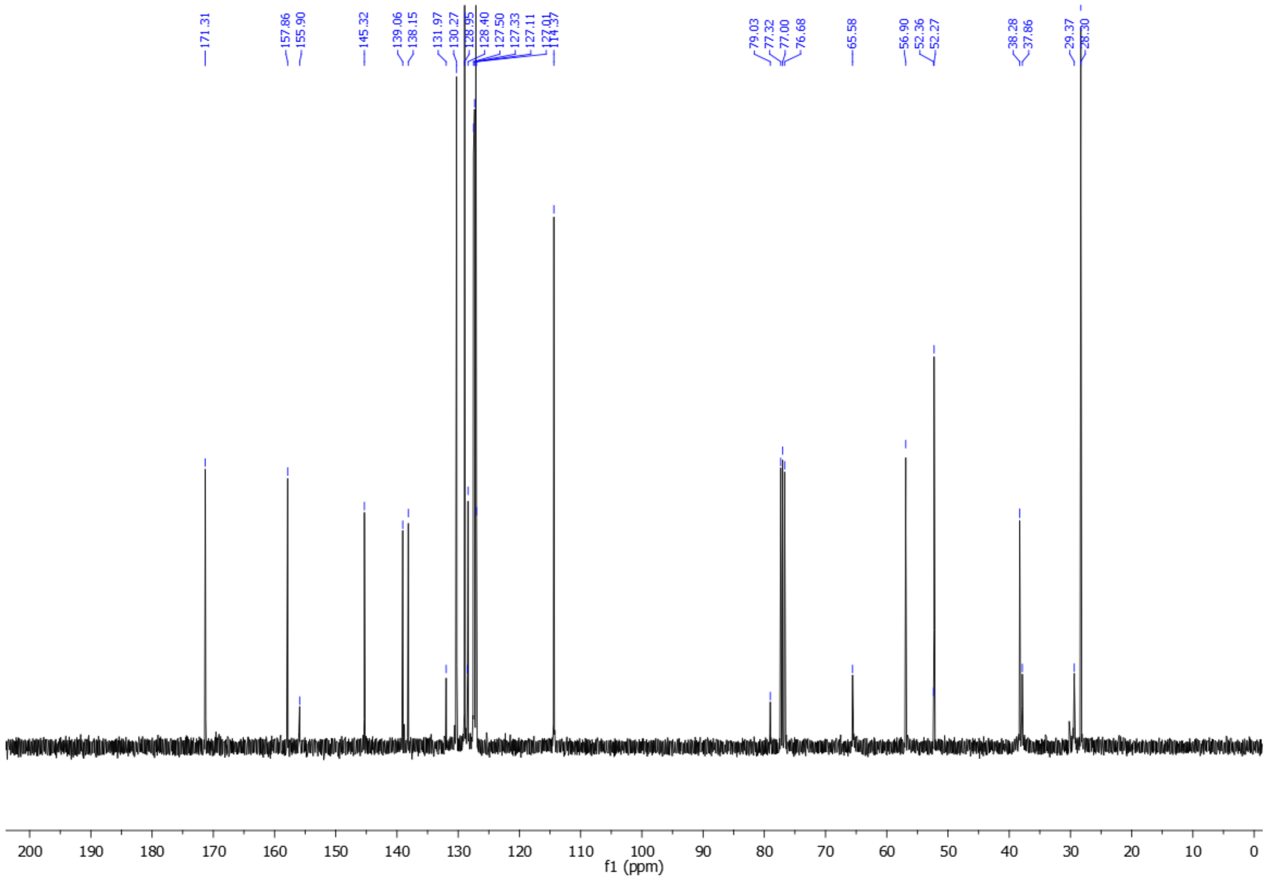

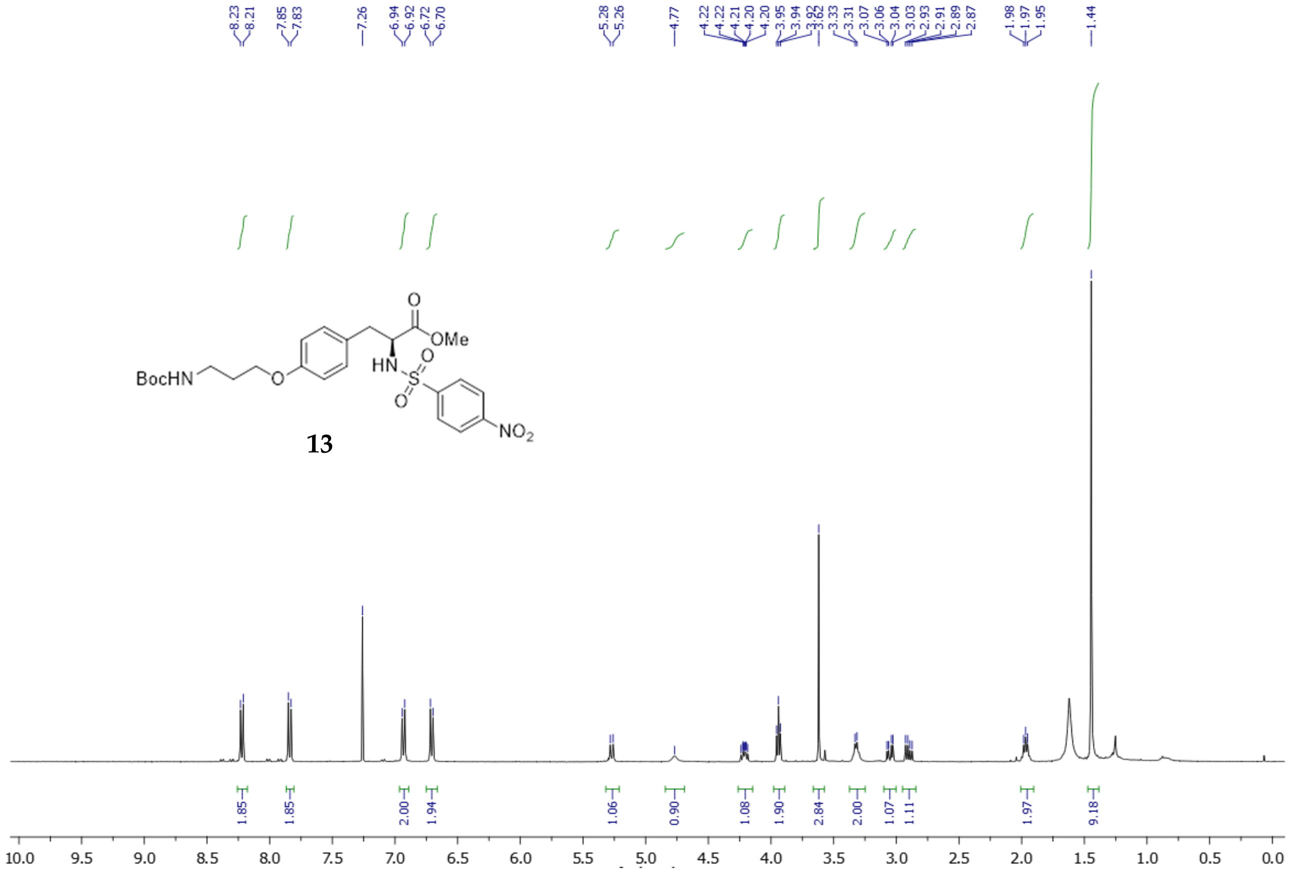
BocHN
O
OMe
HN
S
NO2
13

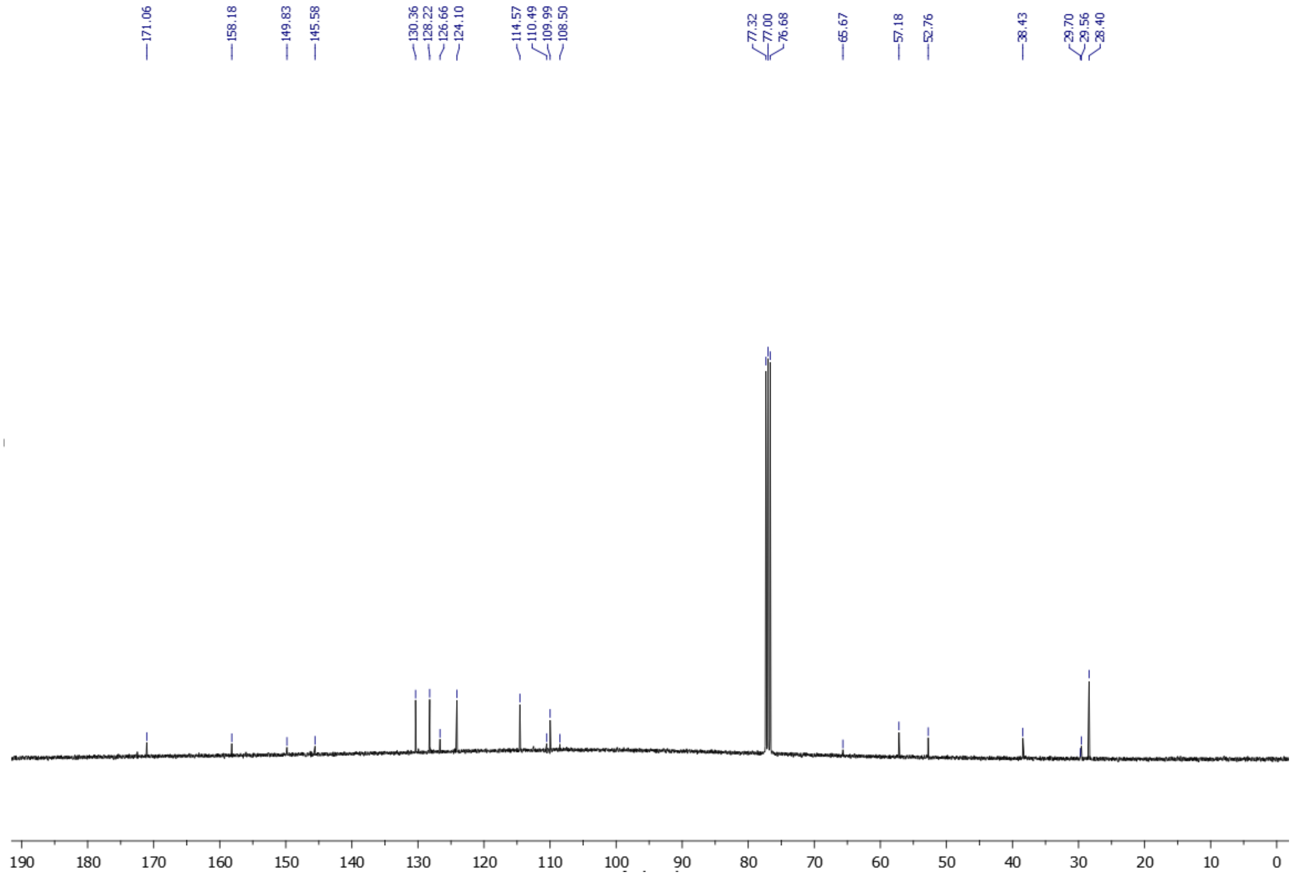

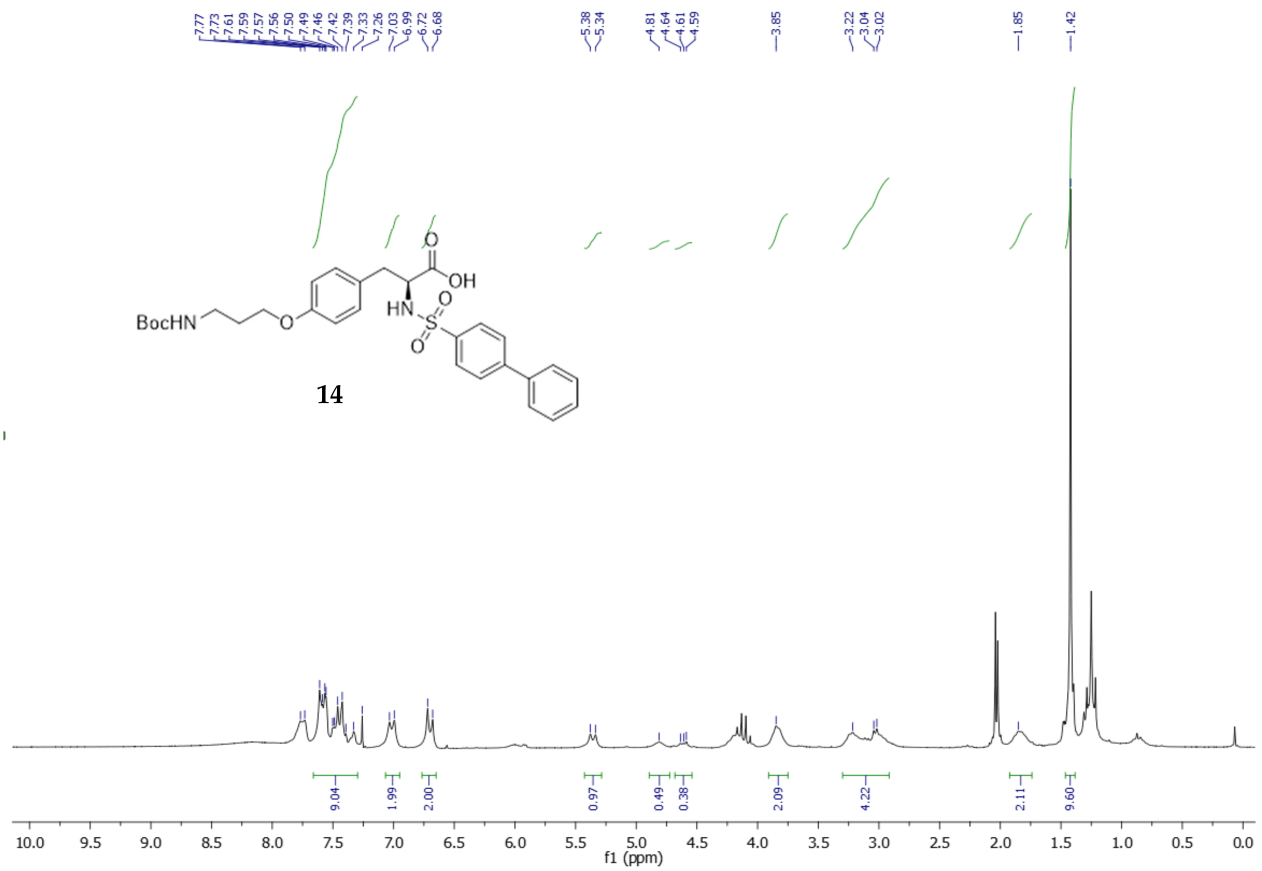
7.77 7.73 7.61 7.59 7.57 7.56 7.50 7.49 7.46 7.42 7.39 7.33 7.26 7.03 6.99 6.72 6.68
5.38 5.34
4.81 4.64 4.61 4.59
3.85
3.22 3.04 3.02
1.85
1.42
BocHN
O
OH
HN
S
14
9.04 1.99 2.00 0.97 0.49 0.38 2.09 4.22 2.11 9.60
10.0 9.5 9.0 8.5 8.0 7.5 7.0 6.5 6.0 5.5 5.0 4.5 4.0 3.5 3.0 2.5 2.0 1.5 1.0 0.5 0.0
f1 (ppm)

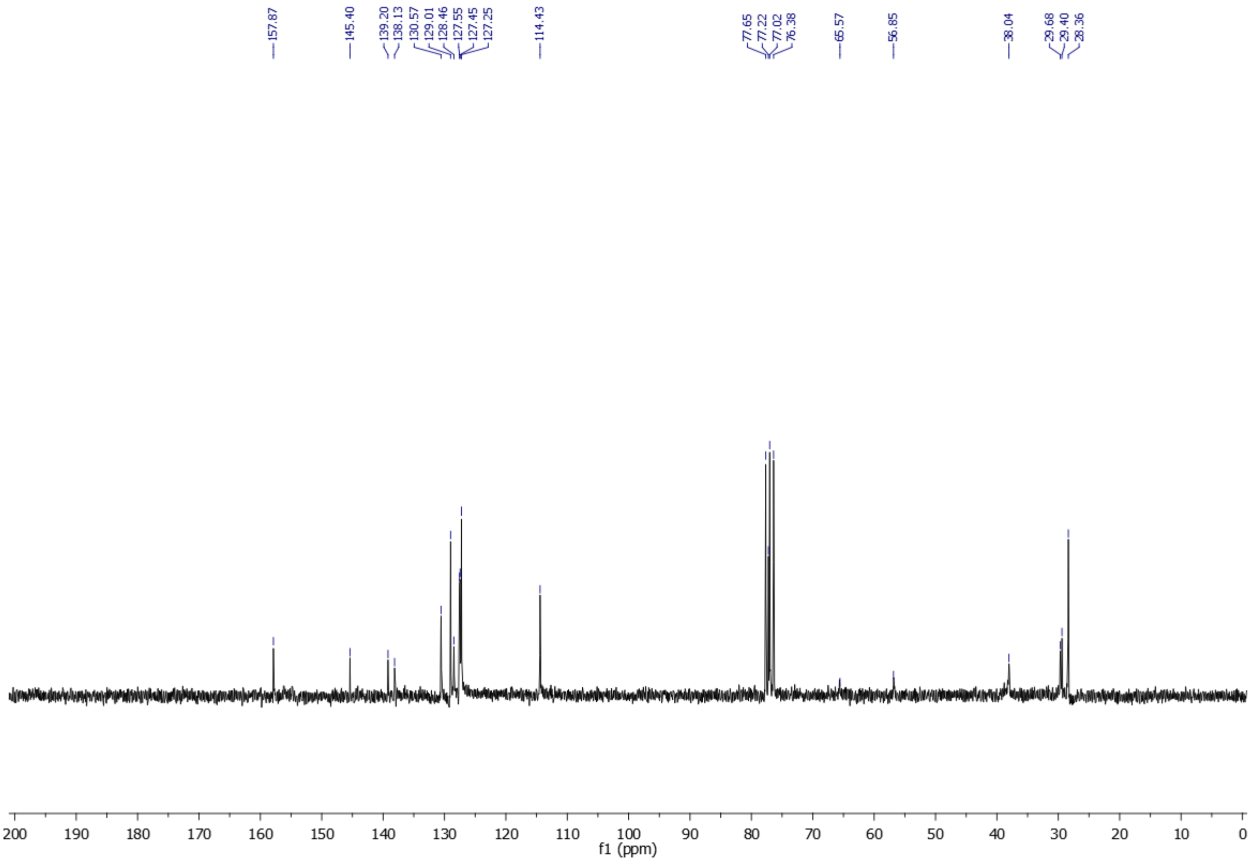
157.87
145.40
139.20 138.13 130.57 129.01 128.46 127.55 127.45 127.25
114.43
77.65 77.22 77.02 76.38
65.57
56.85
38.04
29.68 29.40 28.36
200 190 180 170 160 150 140 130 120 110 100 90 80 70 60 50 40 30 20 10 0
f1 (ppm)

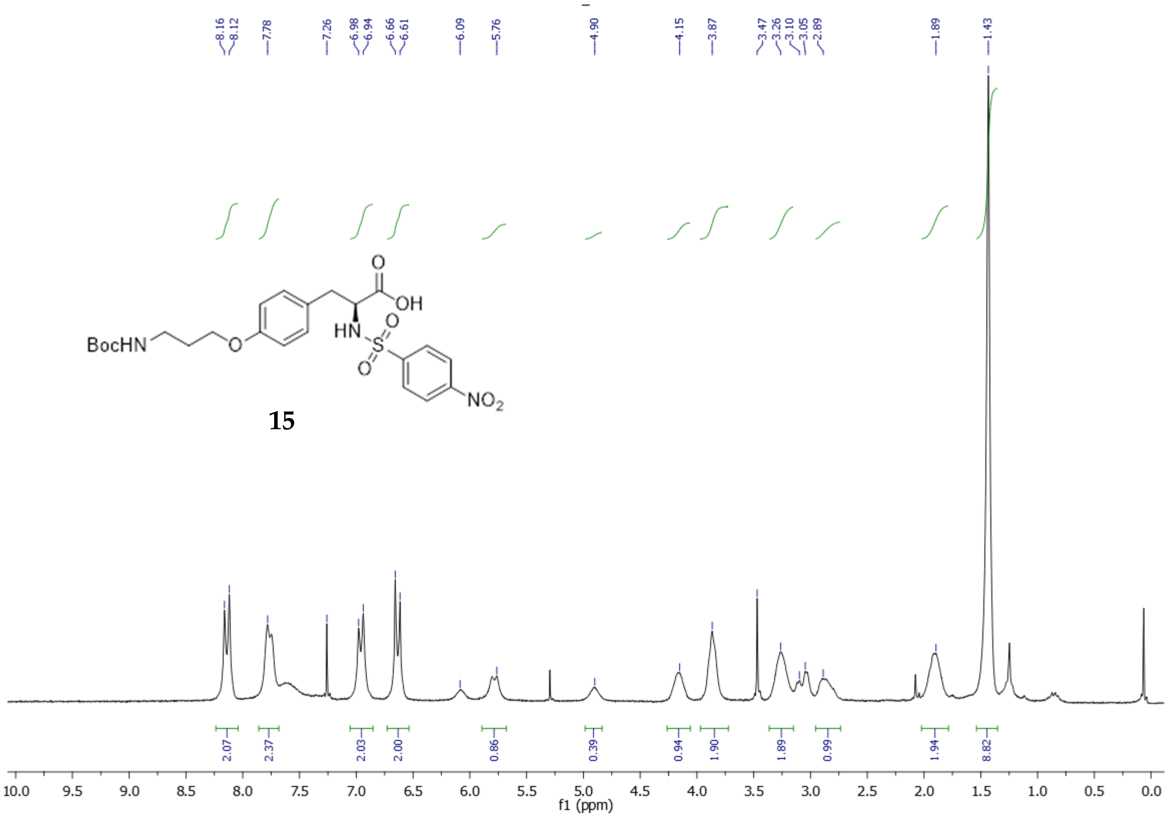
8.16
8.12
7.78
7.26
6.98
6.94
6.66
6.61
6.09
5.76
4.90
4.15
3.87
3.47
3.26
3.10
3.05
2.89
1.89
1.43
BocHN
O
OH
HN
S
NO2
15
2.07
2.37
2.03
2.00
0.86
0.39
0.94
1.90
1.89
0.99
1.94
8.82
10.0 9.5 9.0 8.5 8.0 7.5 7.0 6.5 6.0 5.5 5.0 4.5 4.0 3.5 3.0 2.5 2.0 1.5 1.0 0.5 0.0
f1 (ppm)

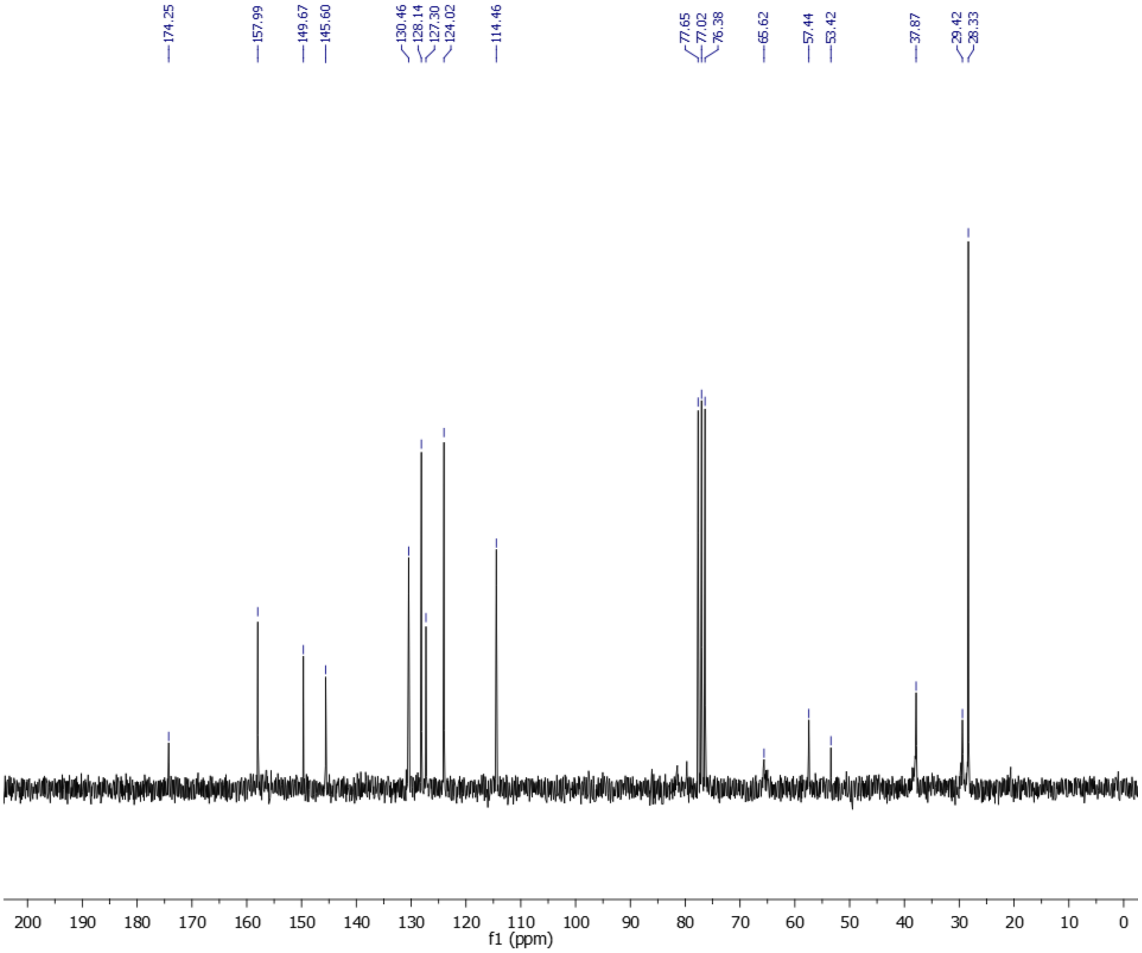
174.25
157.99
149.67
145.60
130.46
128.14
127.30
124.02
114.46
77.65
77.02
76.38
65.62
57.44
53.42
37.87
29.42
28.33
200 190 180 170 160 150 140 130 120 110 100 90 80 70 60 50 40 30 20 10 0
f1 (ppm)

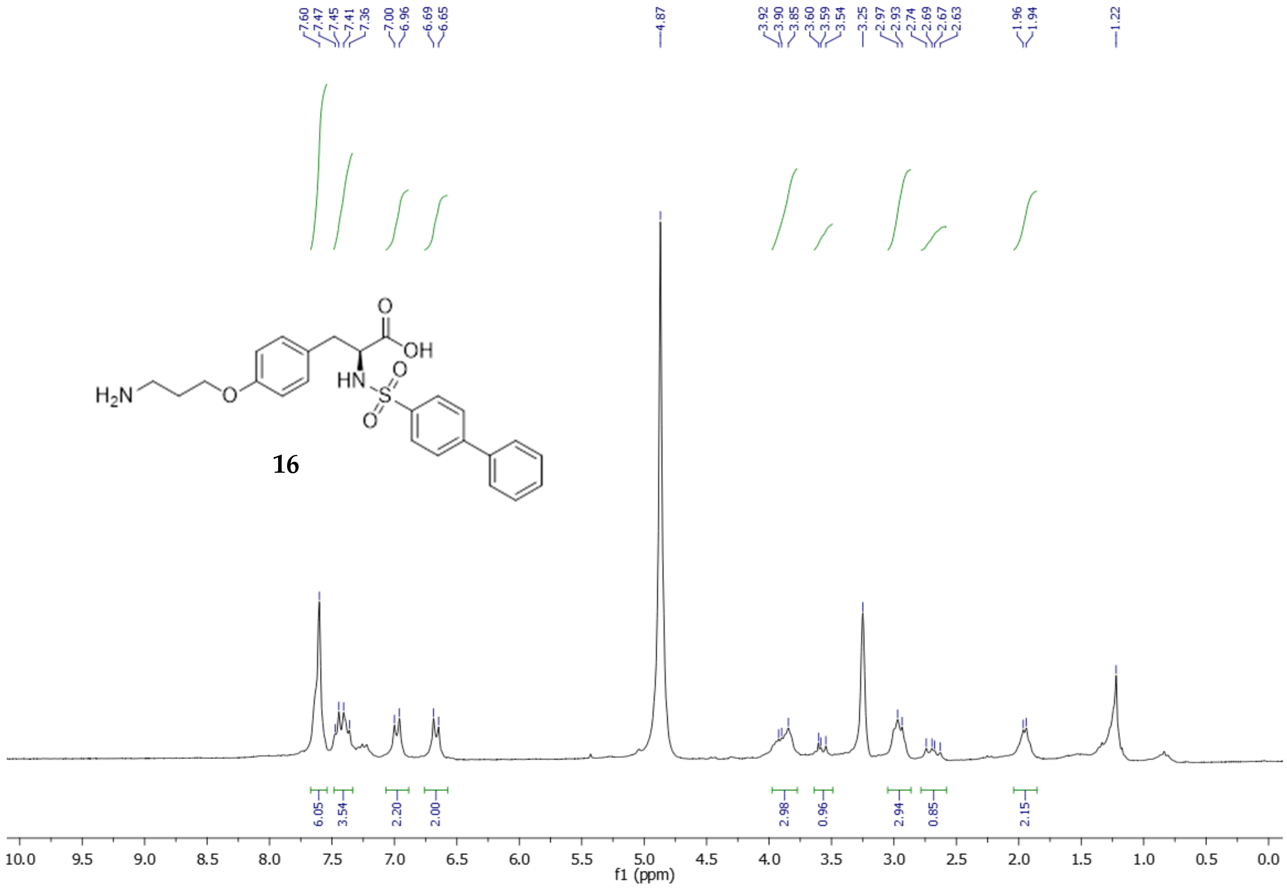
H2N
O
OH
HN
S
16
f1 (ppm)

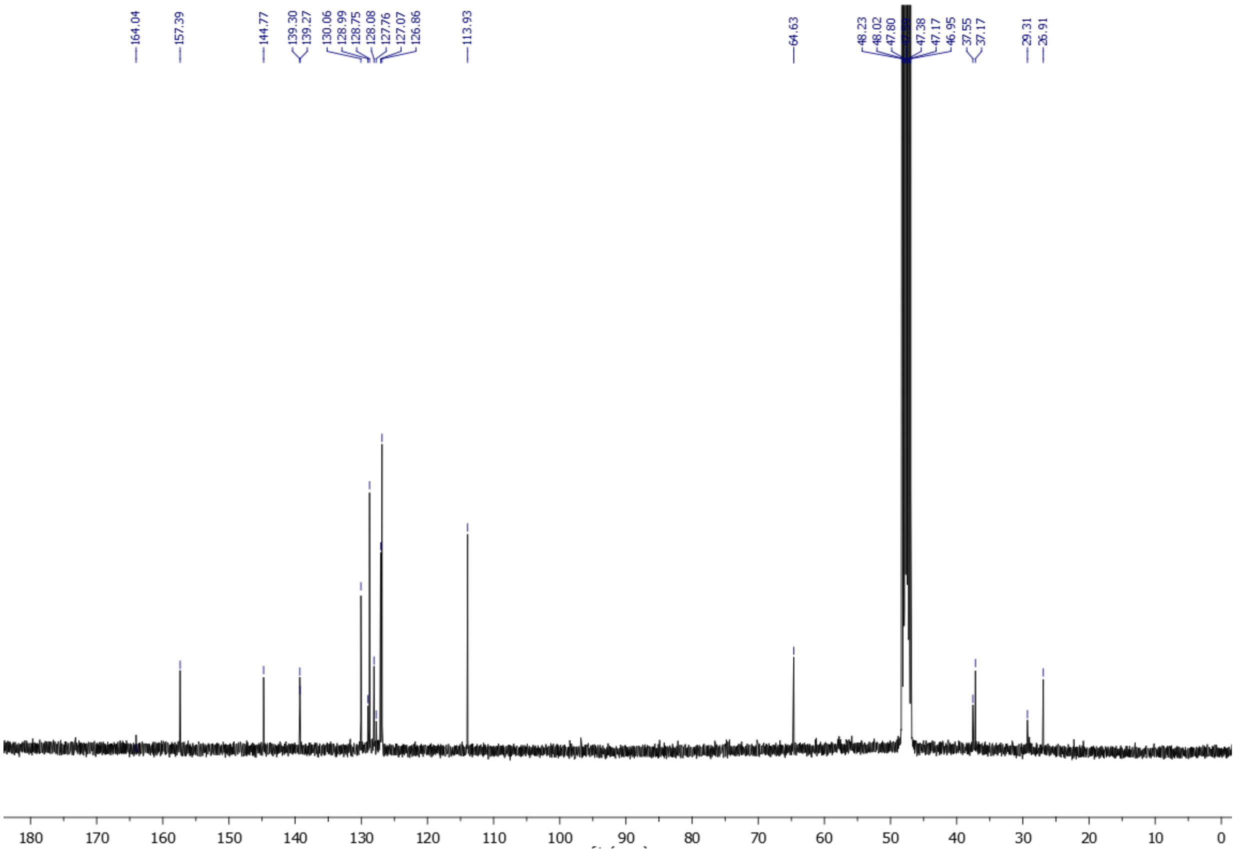

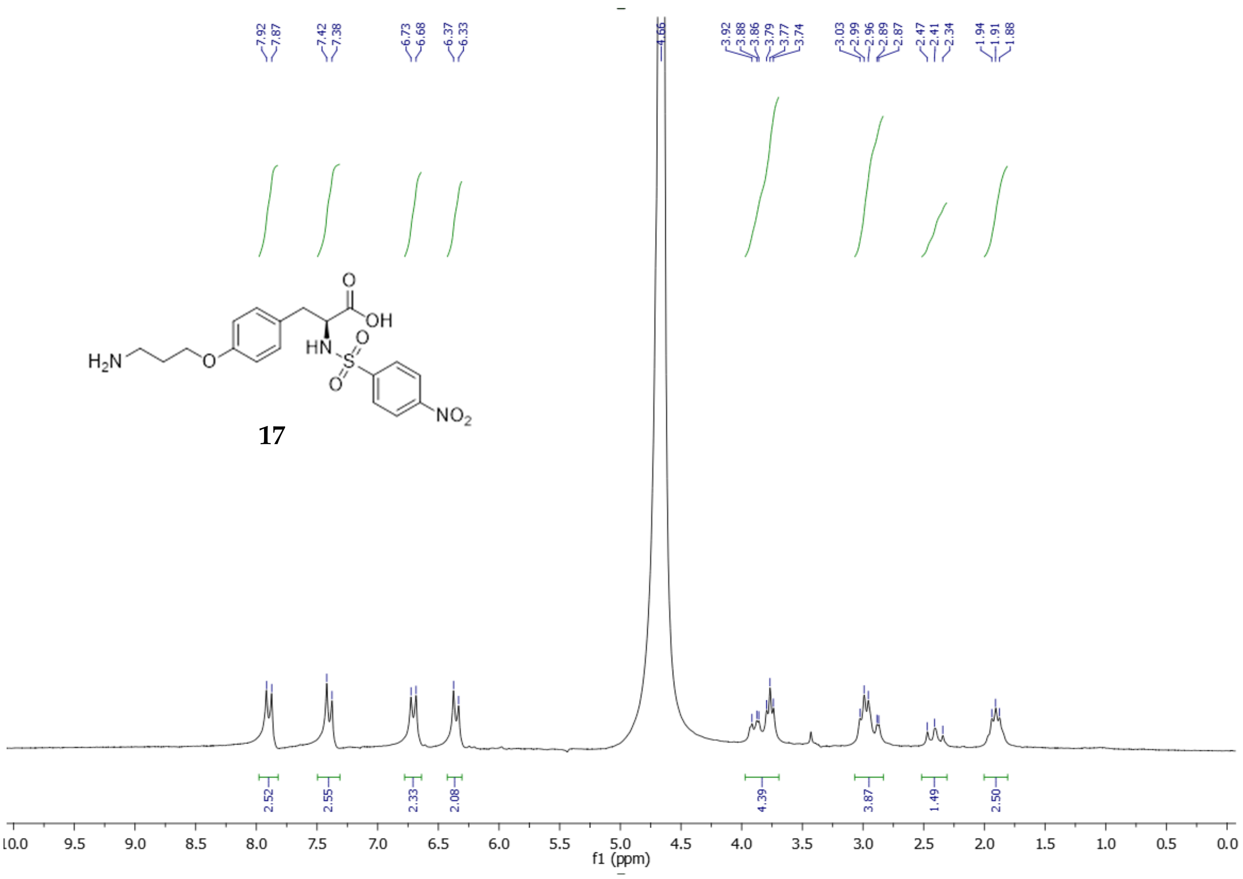
H2N
O
HN
S
OH
NO2
17
f1 (ppm)

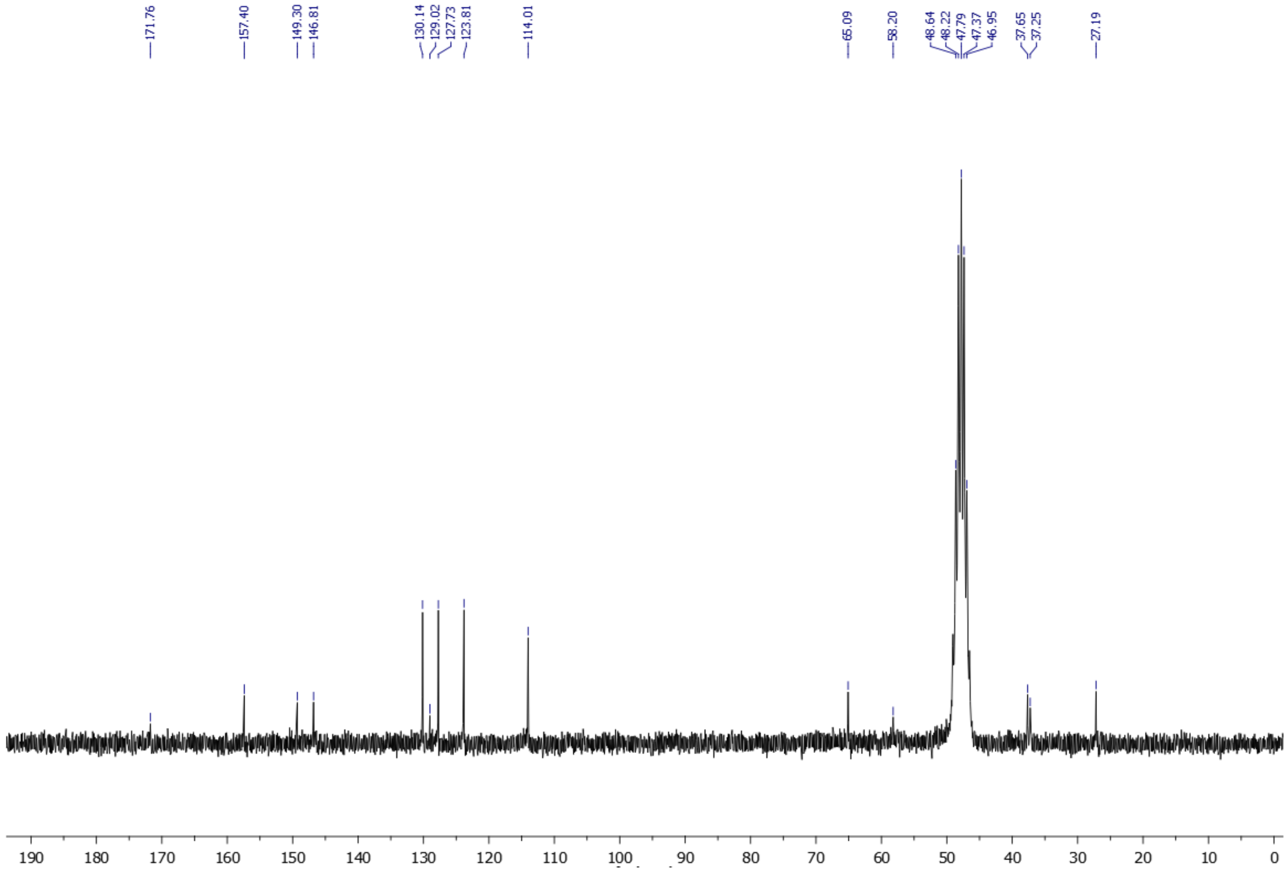

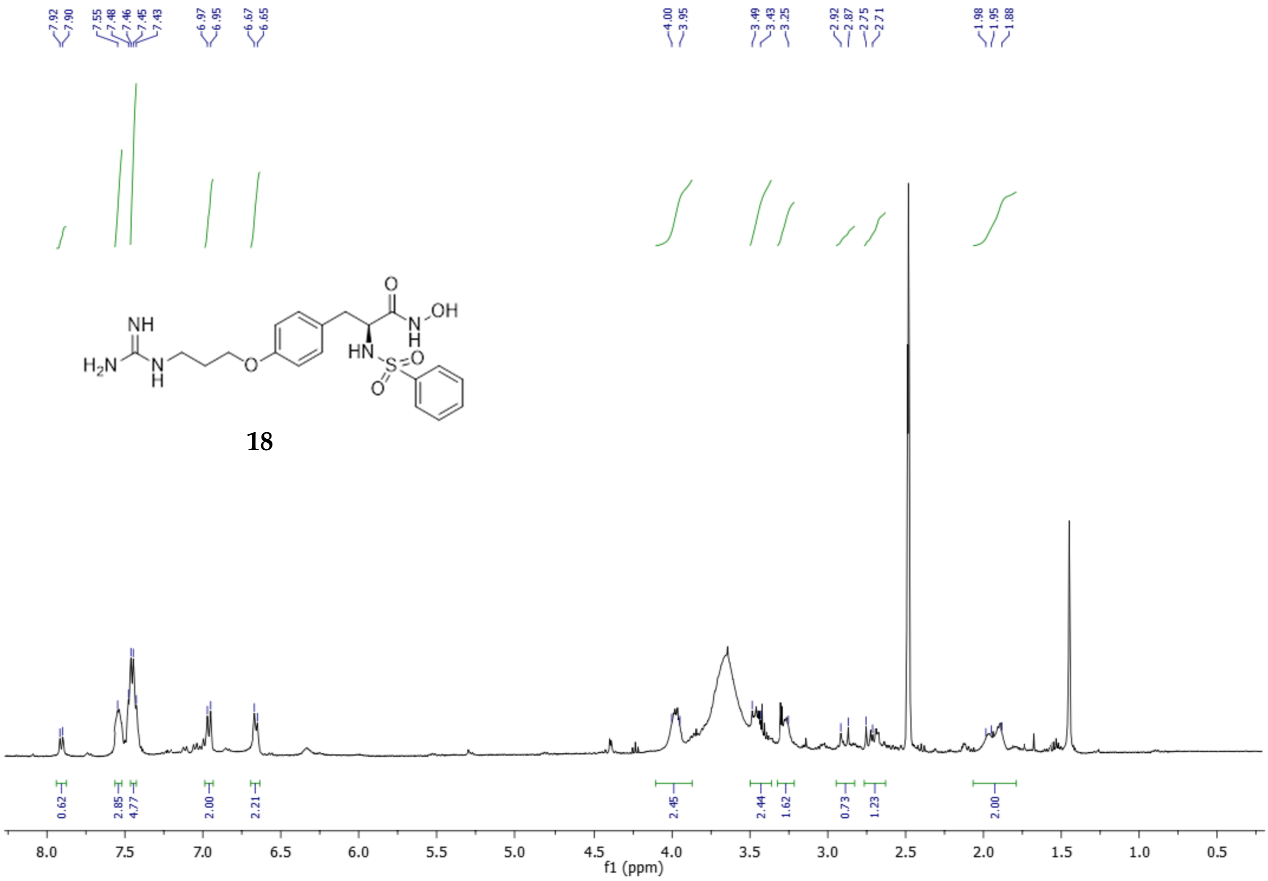
7.92
7.90
7.55
7.48
7.46
7.45
7.43
6.97
6.95
6.67
6.65
4.00
3.95
3.49
3.43
3.25
2.92
2.87
2.75
2.71
1.98
1.95
1.88
NH
H2N
N
H
O
HN
S
O
N
H
OH
18
0.62
2.85
4.77
2.00
2.21
2.45
2.44
1.62
0.73
1.23
2.00
8.0
7.5
7.0
6.5
6.0
5.5
5.0
4.5
4.0
3.5
3.0
2.5
2.0
1.5
1.0
0.5
f1 (ppm)

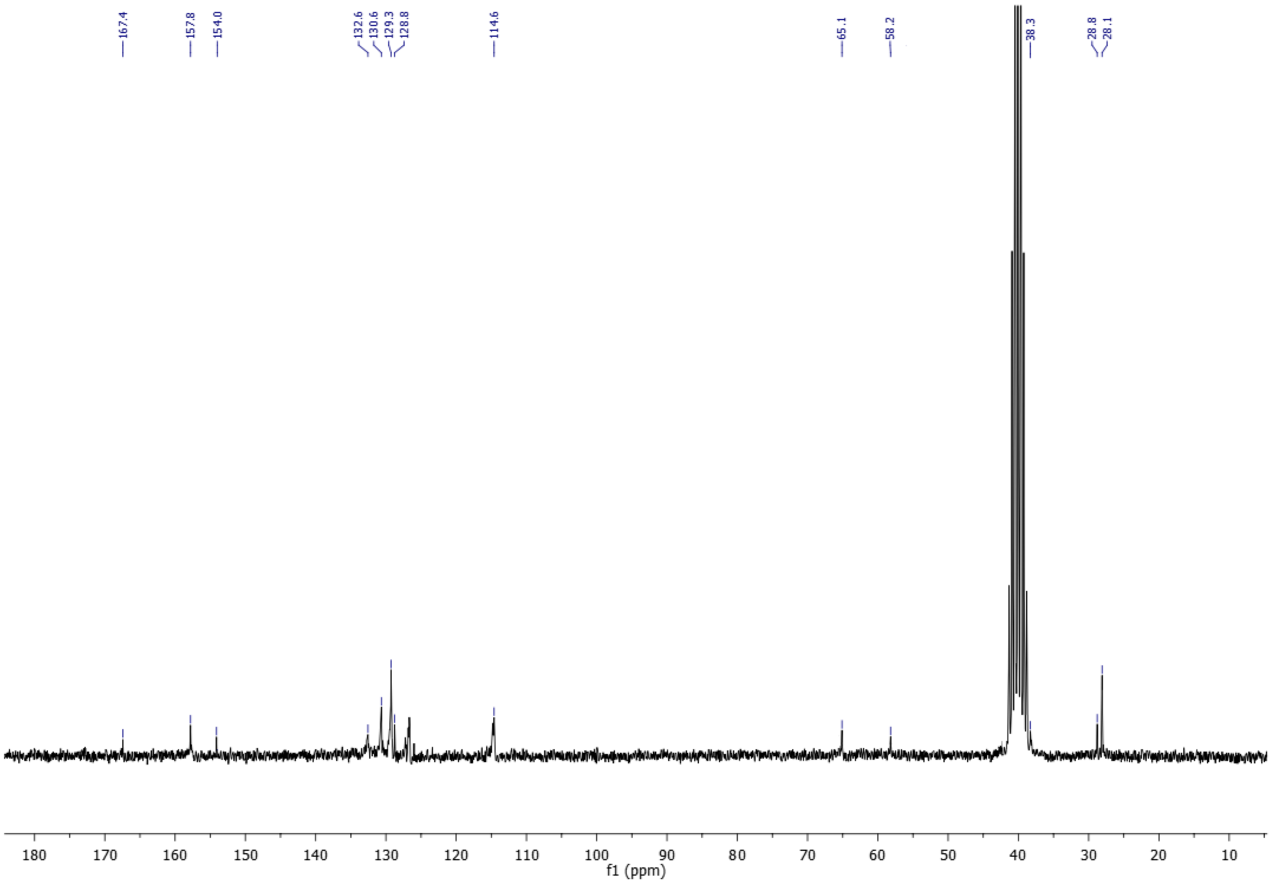
167.4
157.8
154.0
132.6
130.6
129.3
128.8
114.6
65.1
58.2
38.3
28.8
28.1
180
170
160
150
140
130
120
110
100
90
80
70
60
50
40
30
20
10
f1 (ppm)

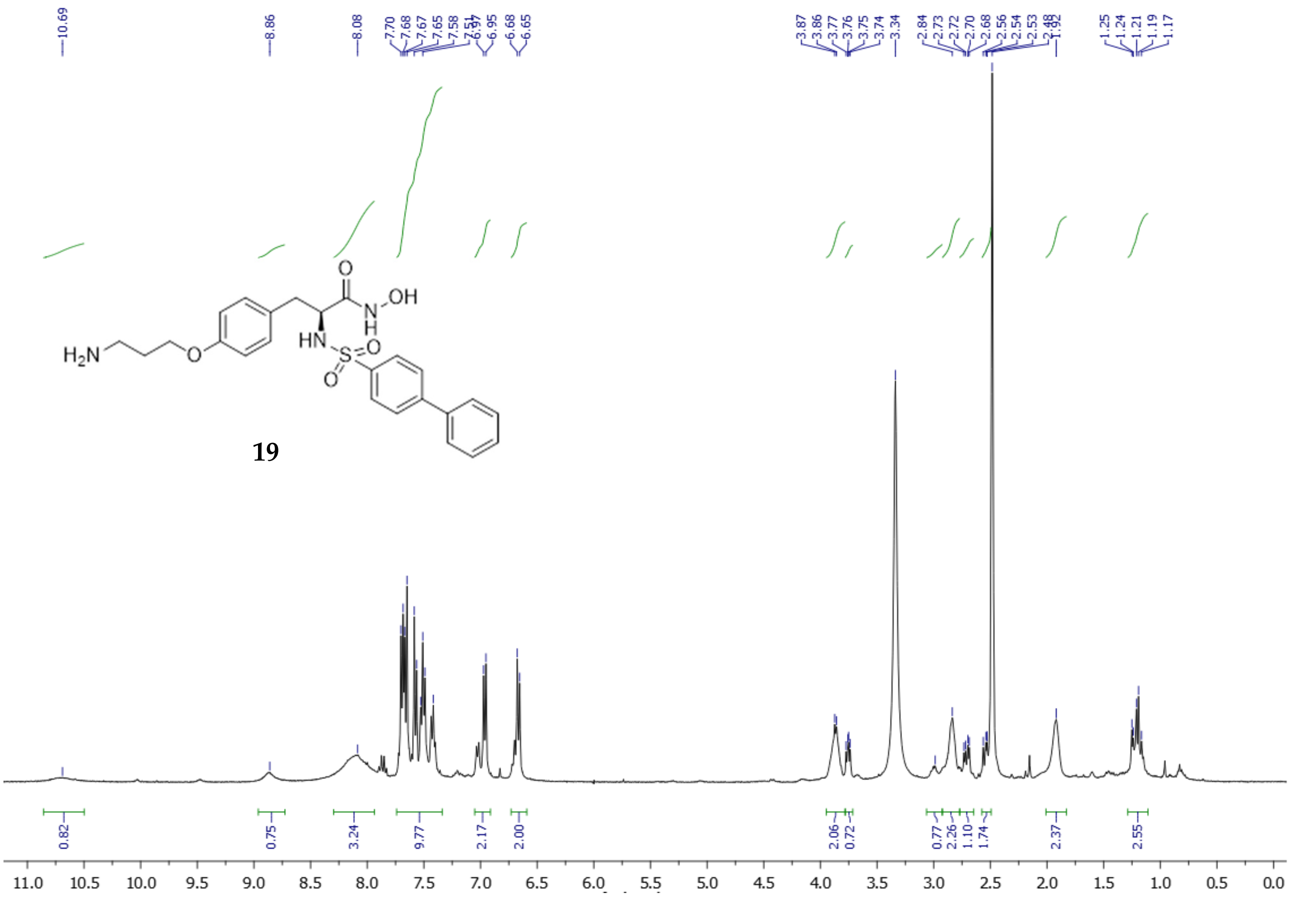
10.69
8.86
8.08
7.70
7.68
7.67
7.65
7.58
7.51
6.97
6.95
6.68
6.65
3.87
3.86
3.77
3.76
3.75
3.74
3.34
2.84
2.73
2.72
2.70
2.68
2.56
2.54
2.53
2.48
1.92
1.25
1.24
1.21
1.19
1.17
O
N
OH
H
H2N
O
HN
S
O
O
19
0.82
0.75
3.24
9.77
2.17
2.00
2.06
0.72
0.77
2.26
1.10
1.74
2.37
2.55
11.0 10.5 10.0 9.5 9.0 8.5 8.0 7.5 7.0 6.5 6.0 5.5 5.0 4.5 4.0 3.5 3.0 2.5 2.0 1.5 1.0 0.5 0.0

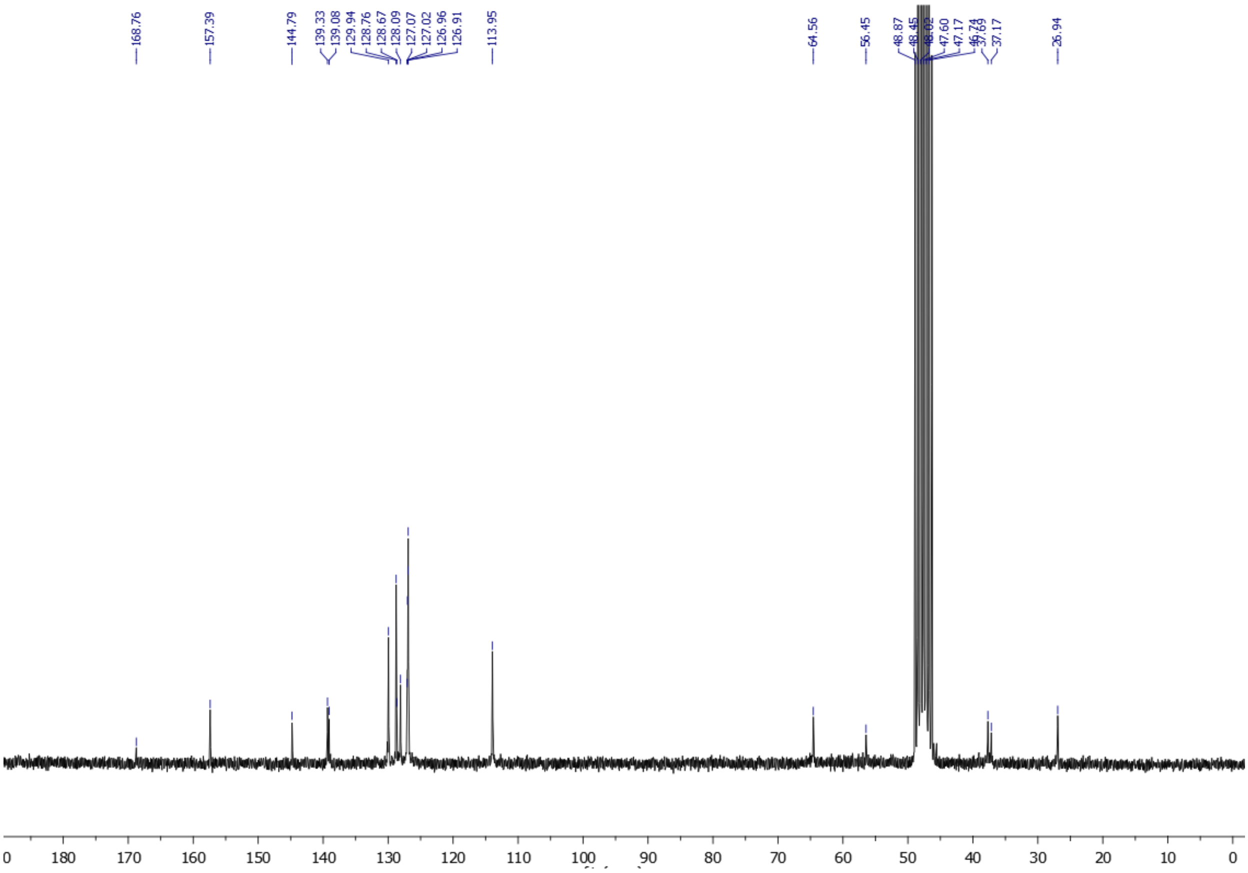
168.76
157.39
144.79
139.33
139.08
129.94
128.76
128.67
128.09
127.07
127.02
126.96
126.91
113.95
64.56
56.45
48.87
48.02
47.60
47.17
37.17
26.94
0 180 170 160 150 140 130 120 110 100 90 80 70 60 50 40 30 20 10 0

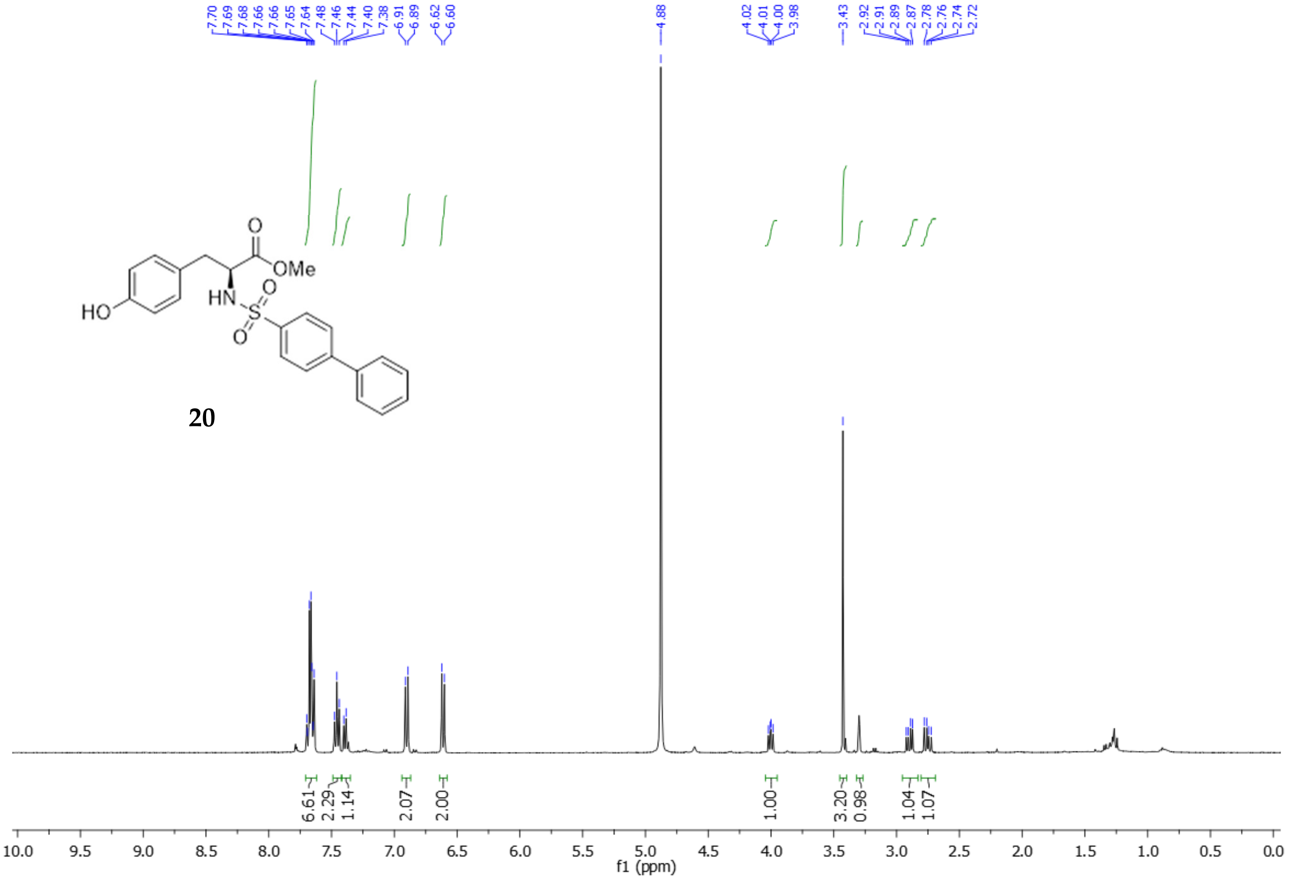
7.70
7.69
7.68
7.66
7.66
7.65
7.64
7.48
7.46
7.44
7.40
7.38
6.91
6.89
6.62
6.60
4.88
4.02
4.01
4.00
3.98
3.43
2.92
2.91
2.89
2.87
2.78
2.76
2.74
2.72
O
OMe
HN
S
O
O
HO
6.61
2.29
1.14
2.07
2.00
1.00
3.20
0.98
1.04
1.07
10.0 9.5 9.0 8.5 8.0 7.5 7.0 6.5 6.0 5.5 5.0 4.5 4.0 3.5 3.0 2.5 2.0 1.5 1.0 0.5 0.0
f1 (ppm)

20

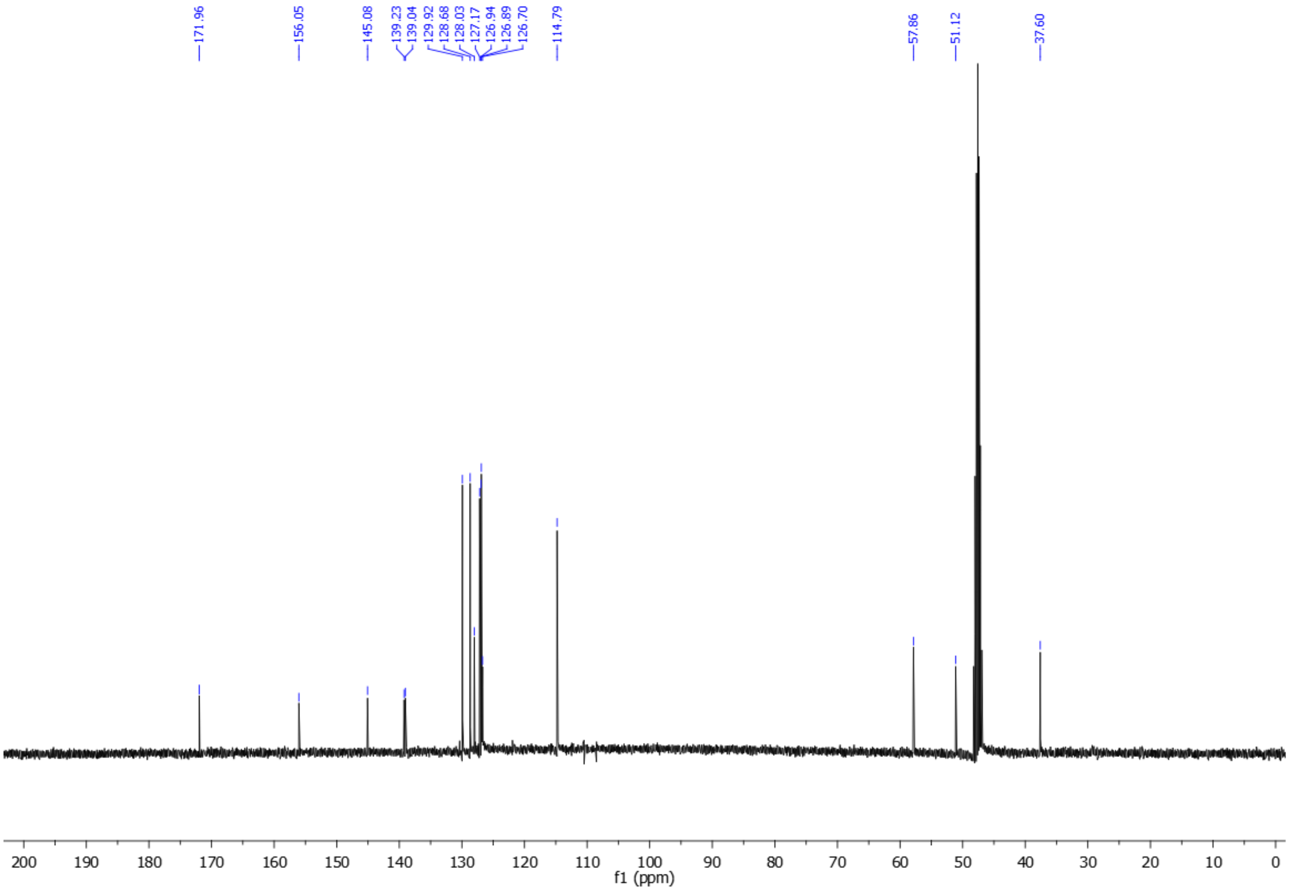
171.96
156.05
145.08
139.23
139.04
129.92
128.68
128.03
127.17
126.94
126.89
126.70
114.79
57.86
51.12
37.60
200 190 180 170 160 150 140 130 120 110 100 90 80 70 60 50 40 30 20 10 0
f1 (ppm)

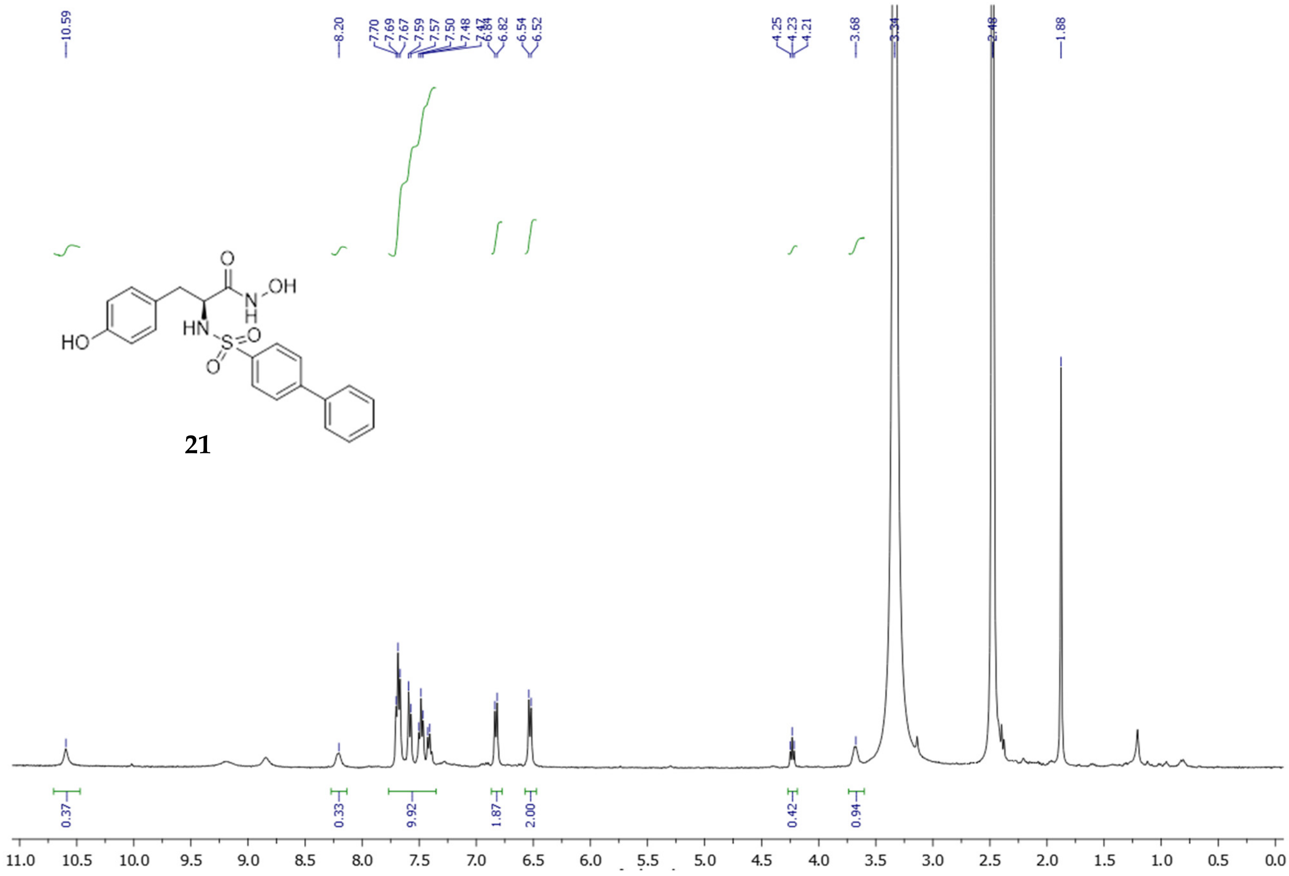
10.59
8.20
7.70
7.69
7.67
7.59
7.57
7.50
7.48
7.47
6.84
6.82
6.54
6.52
4.25
4.23
4.21
3.68
3.34
2.48
1.88
HO
O
N
OH
H
HN
S
O
O
21
0.37
0.33
9.92
1.87
2.00
0.42
0.94
11.0 10.5 10.0 9.5 9.0 8.5 8.0 7.5 7.0 6.5 6.0 5.5 5.0 4.5 4.0 3.5 3.0 2.5 2.0 1.5 1.0 0.5 0.0

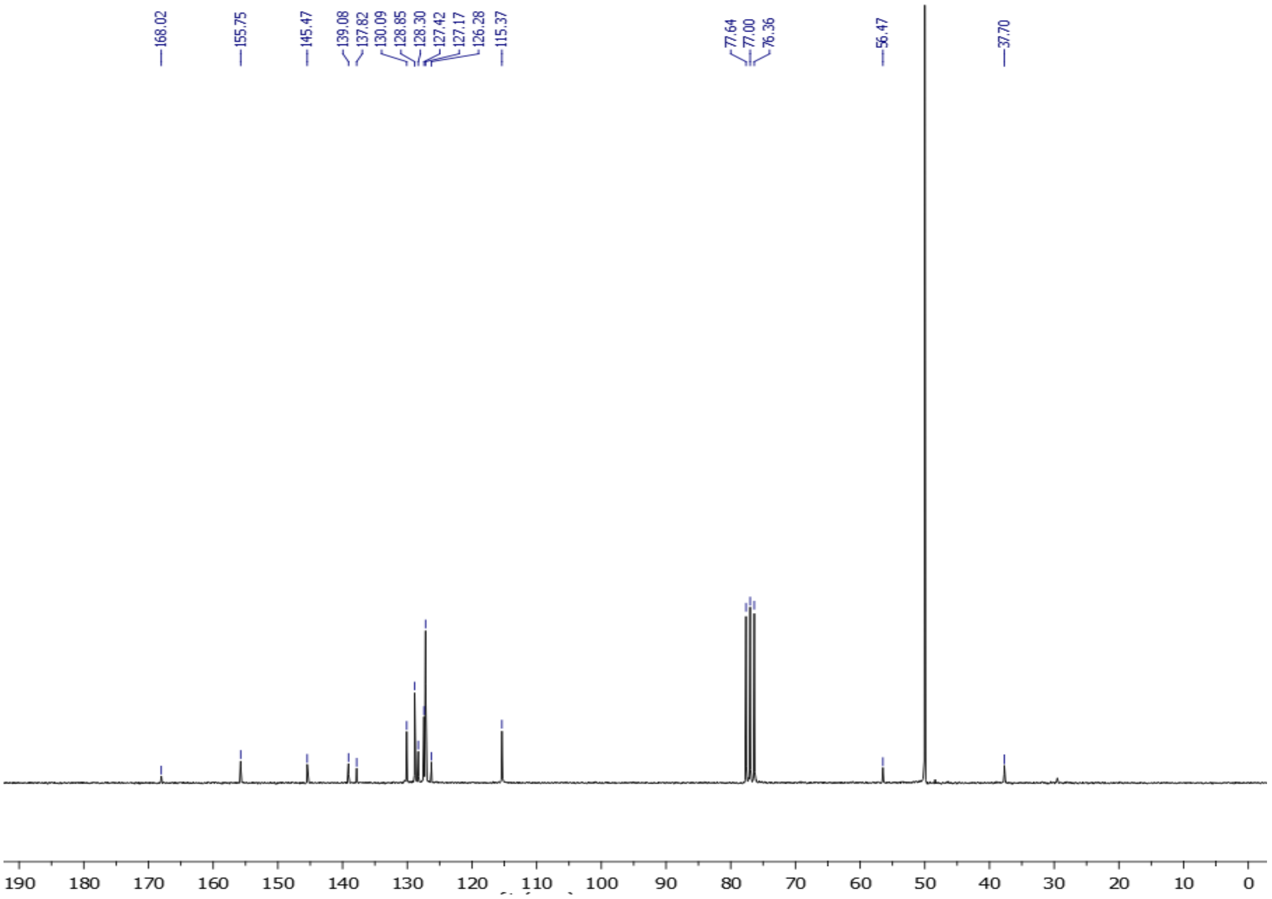
168.02
155.75
145.47
139.08
137.82
130.09
128.85
128.30
127.42
127.17
126.28
115.37
77.64
77.00
76.36
56.47
37.70
190 180 170 160 150 140 130 120 110 100 90 80 70 60 50 40 30 20 10 0
